# Effects of high-intensity interval and moderate-intensity continuous training on overweight or obese college students: A systematic review and meta-analysis

**DOI:** 10.1016/j.isci.2025.114361

**Published:** 2025-12-06

**Authors:** Changzhou Chen, Chuanwen Yu, Sen Li

**Affiliations:** 1 School of Physical Education, Shanghai University of Sport, Shanghai, China; 2 School of Physical Education and Health, Heze University, Heze, Shandong, China; 3 School of Physical Education and Health, Shanghai Lixin University of Accounting and Finance, Shanghai, China

**Keywords:** Human physiology, Human metabolism

## Abstract

Overweight and obesity are increasingly prevalent among college students, yet the comparative effects of high-intensity interval training (HIIT) and moderate-intensity continuous training (MICT) in this population remain unclear. We systematically reviewed randomized controlled trials evaluating the impacts of HIIT and MICT on body composition, lipid profiles, and cardiorespiratory fitness in overweight or obese college students. Twelve studies were included, and the certainty of evidence was assessed using the GRADE approach. Low-to-very low-certainty evidence suggests that both HIIT and MICT may improve body weight, BMI, body fat percentage, and fat mass, while HIIT may offer additional benefits for cardiorespiratory fitness and select lipid markers. However, head-to-head comparisons revealed no consistent statistically significant differences between the two training modalities. Given the overall low certainty of evidence and methodological limitations across studies, these findings should be interpreted cautiously. More rigorous, adequately powered trials are needed to clarify the relative effectiveness of HIIT and MICT for weight management and cardiometabolic health in young adults.

## Highlights

• Demonstrates that both HIIT and MICT improve adiposity-related outcomes in college students
• Shows that HIIT may yield greater gains in cardiorespiratory fitness and lipid metabolism
• Reveals no statistically significant differences between HIIT and MICT across major outcomes
• Applies RoB 2.0 and GRADE frameworks to evaluate evidence quality and result robustness

## Graphical abstract

**Figure:**
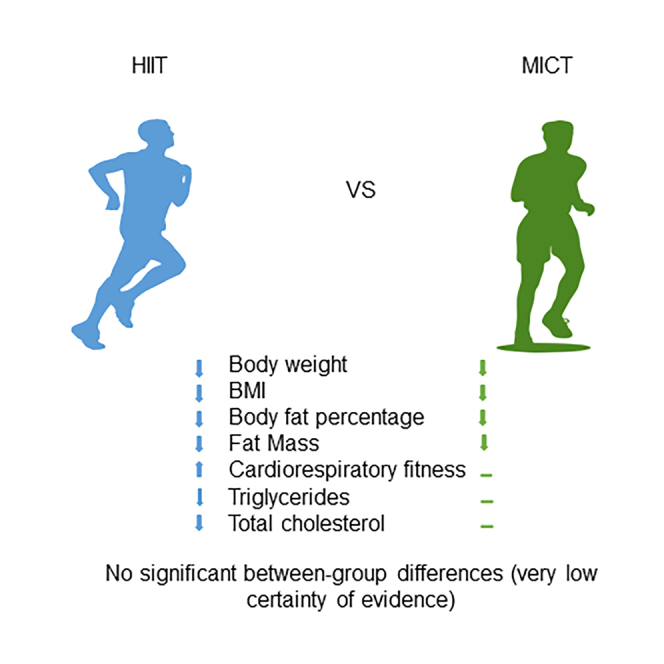

## Introduction

Obesity is a chronic metabolic disease caused by multiple factors, and it has become a significant global public health concern.^1^ Approximately 650 million adults and 340 million adolescents worldwide are estimated to have overweight or obesity, with these numbers steadily increasing.^2^ Excess body weight, particularly when accumulated in the abdominal and visceral areas, significantly burdens the cardiovascular and metabolic systems. This can lead to decreased cardiopulmonary endurance among college students and is strongly linked to hypertension, with the two conditions often occurring together and increasingly affecting younger populations.^3^ Individuals with overweight or obesity frequently exhibit metabolic abnormalities, which can cause organ damage and increase the risk of cardiovascular diseases and diabetes.^4^^,^^5^^,^^6^ Moreover, the economic burden of overweight and obesity is substantial.^7^ By 2050, the annual direct and indirect costs are projected to reach $13.62 and $490.2 billion, respectively.^8^

College students are in a transitional stage from adolescence to adulthood, making them particularly vulnerable to psychological challenges due to physical, emotional, and social changes. These issues can severely impact their academic performance and daily life, and in extreme cases, may even lead to suicidal behavior. However, the prevalence of overweight and obesity among college students has risen dramatically, primarily because of insufficient physical activity and unhealthy lifestyles. This has resulted in a significant decline in cardiopulmonary fitness and an increase in mental health disorders, posing a serious threat to students’ overall health. A study screening 729 college students found that the prevalence of obesity reached 31.9%.^9^ Another study focused on medical students reported an overweight and obesity rate of 24%.^10^ The unique characteristics of college students make this population a critical window for obesity prevention and control. First, individuals aged 18–26 years are in the phase of transition from adolescence to adulthood, during which endocrine function and body composition remain plastic; as growth and development settle toward homeostasis, the responsiveness of energy metabolism, insulin sensitivity, and circadian rhythms to lifestyle interventions may differ from that observed in middle-aged or older adults and school-age populations.^11^^,^^12^^,^^13^ Second, the university milieu-characterized by academic stress, irregular schedules, prolonged sedentary study, high-energy-density take-out diets, limited physical education class time, and fragmented opportunities for exercise leads to insufficient physical activity, alongside declines in cardiorespiratory fitness, thereby exacerbating weight gain and metabolic dysregulation.^14^^,^^15^ Third, weight or appearance concerns, together with mood and sleep problems,^16^ may undermine exercise adherence and also influence intervention effects by altering energy intake or expenditure and non-exercise physical activity.^17^ The convergence of these physiological, psychological, behavioral, and environmental factors positions college students as a priority group for exercise prescriptions that are time-efficient, adherence-friendly, and feasible within campus settings.

Exercise interventions constitute a first-line strategy for weight management and metabolic health improvement. Traditional moderate-intensity continuous training (MICT) has a robust evidence base, demonstrating reductions in adiposity and improvements in lipid and glucose metabolism; however, it often requires longer session durations and higher weekly volumes, which can limit real-world adherence and persistence.^18^^,^^19^ In contrast, high-intensity interval training (HIIT) characterized by brief bouts of vigorous exercise interspersed with low-intensity recovery offers superior time efficiency and can enhance cardiorespiratory fitness over relatively short intervention periods. By enhancing excess post-exercise oxygen consumption and fatty acid oxidation, HIIT may confer additional benefits for body composition and metabolic regulation.^20^^,^^21^^,^^22^

Several systematic reviews have examined the effects of HIIT and MICT on body weight and fat reduction in populations with overweight or obesity. For example, Su et al. compared the impacts of HIIT and MICT on cardiovascular risk factors in adults with overweight or obesity and found similar outcomes regarding the body composition; however, HIIT was more effective in enhancing cardiorespiratory fitness.^23^ Likewise, a systematic review by Wewege et al. demonstrated that both HIIT and MICT led to comparable improvements in the body composition among adults with overweight or obesity.^24^ Chen et al. concluded that HIIT and aerobic training were the most effective exercise modalities for fat loss.^25^ Conversely, some studies have reported that HIIT does not provide superior effects on body fat reduction.^21^ Furthermore, randomized controlled trials (RCTs) have explored the effects of HIIT and MICT in college students with overweight or obesity. Hu et al. reported similar intervention effects between HIIT and MICT in this population,^26^ whereas Zhu et al. found that HIIT was more effective than MICT.^27^ Systematic reviews and RCTs targeting populations with overweight or obesity generally indicate that HIIT and MICT yield comparable or only modestly different effects on the body composition, whereas HIIT more frequently shows advantages in cardiorespiratory fitness and certain metabolic parameters (e.g., triglycerides and fasting glucose). However, some studies suggest that HIIT is not consistently superior to MICT across all adiposity-related outcomes, highlighting methodological and population-level heterogeneities.

Existing evidence frequently pools disparate age groups (adolescents, young adults, middle-aged, and older adults), which “averages out” the true effects and prescription sensitivity of this transitional college population. Moreover, in university settings, the dose–response profile of interventions, including training frequency, total number of sessions, intervention duration, whether diet is monitored, and their moderating impacts on body weight and BMI, remain insufficiently defined. In addition, the comparative effectiveness of HIIT versus MICT in populations with overweight or obesity is still debated, and no studies have specifically examined their differential effects among college students with overweight or obesity.

Against this backdrop, our study involves a meta-analysis to systematically evaluate the intervention effects of HIIT and MICT in college students with overweight or obesity; reassess their comparative effectiveness; and investigate potential moderators of body weight and BMI for each exercise modality. The aim is to provide evidence-based guidance for developing scientifically rigorous and pragmatically effective exercise prescriptions for this population.

## Results

### Literature search results

**Figure 1:**
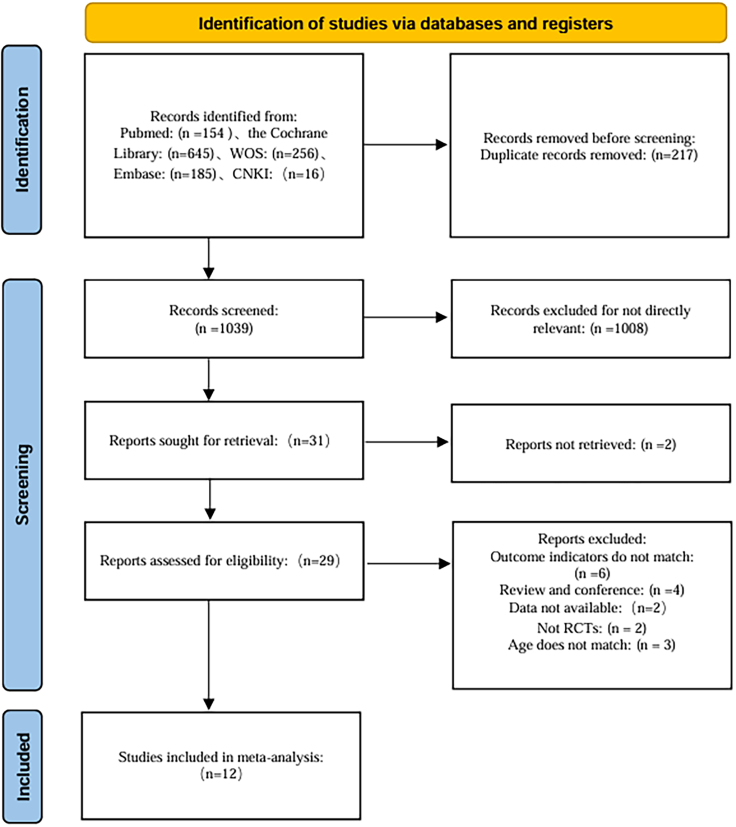
Literature screening flowchart This figure illustrates the study selection process following PRISMA guidelines, including the number of records identified, screened, excluded, and finally included in the meta-analysis.

A total of 1,256 studies were included in this research, with 154 from PubMed, 645 from the Cochrane Library, 256 from WoS, 185 from Embase, and 16 from CNKI. After removing 217 duplicate records, 1,039 studies were screened by reviewing the titles and abstracts, excluding 1,008 irrelevant studies. Full texts of the remaining 31 studies were downloaded, but those of two studies could not be retrieved. Thus, a full-text review was conducted on the 29 studies. Six studies were excluded due to irrelevant outcome measures, 2 studies could not provide accessible data, 4 studies were conference proceedings or reviews, 2 were not RCTs, and 3 enrolled populations that did not meet the eligibility criteria. Ultimately, 12 studies were included in the final analysis.^28^^,^^29^^,^^30^^,^^31^^,^^32^^,^^33^^,^^34^^,^^35^^,^^36^^,^^37^^,^^38^^,^^39^ See Figure 1.

### Basic information of the included studies

A total of 12 RCTs were included, comprising 7 three-arm studies^28^^,^^29^^,^^30^^,^^31^^,^^32^^,^^34^^,^^36^ and 5 two-arm studies,^33^^,^^35^^,^^37^^,^^38^^,^^39^ with a total of 476 college students with overweight or obesity. The mean age of the participants ranged from 19.42 to 22.22 years, and the mean BMI ranged from 25.43 to 31.14 kg/m^2^. Five studies did not report baseline cardiorespiratory fitness.^35^^,^^36^^,^^37^^,^^38^^,^^39^ Dietary monitoring and recording were implemented in five studies^28^^,^^31^^,^^32^^,^^37^^,^^38^; five studies instructed the participants to maintain their usual diets^29^^,^^30^^,^^33^^,^^35^^,^^36^; and two did not report dietary practices.^34^^,^^39^ Three studies enrolled mixed-sex samples,^36^^,^^37^^,^^39^ whereas the remainder included women-only samples. Intervention duration ranged from 8 weeks^28^^,^^36^ to 12 weeks.^29^^,^^30^^,^^31^^,^^32^^,^^33^^,^^34^^,^^35^^,^^37^^,^^38^^,^^39^ Exercise interventions included both HIIT and MICT. Training frequency ranged from three sessions^28^^,^^39^ and 3–4 sessions^31^^,^^32^^,^^36^ to four sessions^30^^,^^33^^,^^38^ and five sessions.^29^^,^^34^^,^^35^^,^^37^ Outcome measures included weight, BMI, percentage of body fat, fat mass, peak oxygen uptake, blood pressure, triglycerides, total cholesterol, high-density lipoprotein, and low-density lipoprotein. Refer to Tables 1 and 2.

**Table 1:** Basic characteristics of included RCTs

Study	Sample size	Age	BMI	Female%	Baseline cardiorespiratory fitness	Whether diet was monitored or controlled	Outcome measures
Eimarieskandari^28^	HIIT:7; MICT:7; C:6	22.22	29.48	100%	HIIT:20.4; MICT:19.65; C:19.35;VO_2_peak (mL/kg^−1^ min^−1^)	maintain usual diet with dietary recording	(1)(2)(3)(4)
Sijie^29^	HIIT:17; MICT:16; C:19	19.53	28.27	100%	HIIT:33.3; MICT:32.9; C:32.8;VO_2_max	maintain usual diet	(1)(2)(3)(6)(7)
Zhang^30^	HIIT:12; MICT:12; C:11	20.83	25.73	100%	HIIT:33.1; MICT:33; C:30.3;VO_2_max (mL·min^−1^·kg^−1^)	maintain usual diet	(1)(2)(3)(4)(6); (8)(9)
Zhang^31^	HIIT:15; MICT:15; C:13	21.07	25.90	100%	Unclear	maintain usual diet with dietary recording	(1)(3)
Zhang^32^	HIIT:12; MICT:11; C:13	20.63	25.43	100%	HIIT:28.7; MICT:28.9; C:28.9;VO_2_peak (mL/kg/min)	maintain usual diet with dietary recording	(1)(3)(8)(9)
Liu^33^	HIIT:20; MICT:20	21.5	28.79	100%	HIIT:32.27; MICT:32.01; VO_2_max (mL·min^−1^·kg^−1^)	maintain usual diet	(1)(2)(3)(6)(8)(9); (10)(11)
Qi^34^	HIIT:20; MICT:20; C:20	20	28.27	100%	HIIT:33.27; MICT:32.9; C:32.8; (mL·min^−1^·kg^−1^)	unclear	(1)(2)(3)(6)
Zhao^35^	HIIT:18; MICT:19	20	31.14	100%	Unclear	maintain usual diet	(1)(2)(4)
Shi^36^	HIIT:21; MICT:22; C:9	20	30.24	0%	Unclear	maintain usual diet	(1)(2)(7)(8)(9)(10); (11)
Gao^37^	HIIT:17; MICT:17	21.6	27.49	50%	Unclear	maintain usual diet with dietary recording	(1)(2)(3)(8)(9)(10); (11)
Wang^38^	HIIT:22; MICT:21	19.42	29.92	100%	Unclear	maintain usual diet with dietary recording	(1)(2)(4)(8)(9)(10); (11)
Wang^39^	HIIT:12; MICT:12	20.8	28.75	58.33%	Unclear	unclear	(2)(7)

Note: (1) for weight; (2) for BMI; (3) for percentage of body fat; (4) for fat mass; (5) for peak oxygen uptake; (6) for VO_2_max; (7) for blood pressure; (8) for triglyceride; (9) for total cholesterol; (10) for high-density lipoprotein; (11) for low-density lipoprotein. HIIT, high-intensity interval training; MICT, moderate-intensity continuous training; C, control.

**Table 2:** Intervention characteristics of the included studies

Study	Intervention	Intervention duration/frequency
Eimarieskandari^28^	HIIT: 4 × 4 min running at 80–90% VO_2_peak with 3 × 3 min at 50–60% VO_2_peak. Total: 25 min; MICT: 41 min running at 50–70% VO_2_peak. Total: 41 min; C: Daily activity	8 weeks/3 days a week
Sijie^29^	HIIT: 5 × 3 min running at 85% VO_2_max with 4 × 3 min at 50–60% VO_2_max. Total: 27 min; MICT: 40 min running at 50% VO_2_max. Total: 40 min; C: Daily activity	12 weeks/5 days a week
Zhang^30^	HIIT: 4 × 4 min running at 85–95% HRpeak with 3 × 3 min walking at 50–60% HRpeak. Total: 25 min; MICT: 33 min running at 60–70% HRpeak. Total: 33 min; C: Daily activity	12 weeks/4 days a week
Zhang^31^	HIIT: 4 min cycling at 90% VO_2_max with 3 min passive recovery until 200–300 kJ of work is achieved. Total: Unclear; MICT: Cycling at 60% VO_2_max until 200–300 kJ of work is achieved. Total: Unclear.; C: Daily activity	12 weeks/3–4 days a week
Zhang^32^	HIIT: 4 min cycling at 90% VO_2_max with 3 min passive recovery until 200 kJ of work is achieved. Total: Unclear; MICT: Cycling at 60% VO_2_max until 200 kJ of work is achieved. Total: Unclear; C: Daily activity	12 weeks/3–4 days a week
Liu^33^	HIIT: 15 × 1 min cycling at 90% VO_2_max with 1 min cycling at 20% VO_2_max. Total: 30 min; MICT: 30 min cycling at 50% VO_2_max. Total: 30 min	12 weeks/4 days a week
Qi^34^	HIIT: 5 × 3 min running at 85% VO_2_max with 2 min running. Total: 25 min; MICT: 50 min running at 40–60% VO_2_max. Total: 50 min; C: Daily activity	12 weeks/5 days a week
Zhao^35^	HIIT: 5 × 5 min cycling at 90–95% HRmax with 5 × 3 min cycling at 50–60% HRmax. Total: 40 min; MICT: 40 min cycling at 60–70% HRmax. Total: 40 min	12 weeks/5 days a week
Shi^36^	HIIT: 3 × 40–60 s exercise at 80–95% HRmax with 3 × 20 s rest. Total: 8–28 min; MICT: 45 min exercise at 60–70% HRmax. Total:45 min; C: Daily activity	8 weeks/3–4 days a week
Gao^37^	HIIT: 5 × 4 min running at 85% VO_2_max with 5 × 2 min running at 50% VO_2_max. Total: 30 min; MICT: 40 min running at 60% VO_2_max. Total: 40 min	12 weeks/5 days a week
Wang^38^	HIIT: 5–6 × 2 min running at 90–95% HRmax with 2 min recovery. Total: 20–24 min.; MICT: 30–60 min running at 60–70% HRmax. Total: 30–60 min	12 weeks/4 days a week
Wang^39^	HIIT: 2 min running at 90–95% HRmax with 2 min recovery. Total: 20 min; MICT: 30–40 min running at 60–70% HRmax. Total: 20 min	12 weeks/3 days a week

Note: HIIT, high-intensity interval training; MICT, moderate-intensity continuous training; C, control; VO_2_peak, peak oxygen uptake; VO_2_max, maximal oxygen consumption; HRpeak, peak heart rate; HRmax, maximum heart rate.

### Risk-of-bias assessment of the included studies

**Figure 2:**
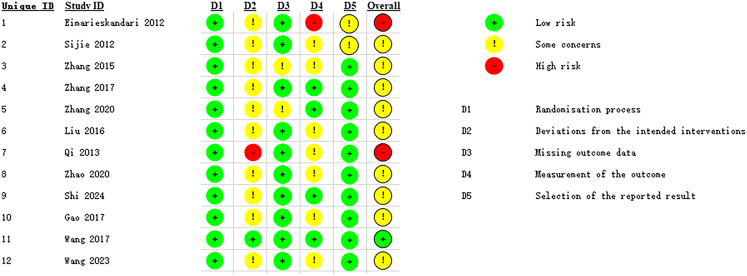
Summary of risk of bias for included randomized controlled trials This figure summarizes the RoB 2.0 assessment across five domains for each included study, indicating judgments of low risk, some concerns, or high risk.

Among the included studies, twelve were rated as low risk in the “randomization process” domain. Ten studies showed some concerns in the “deviations from the intended interventions” domain. Ten studies were assessed as having low risk in the “missing outcome data” domain. Four studies were assessed as having low risk in the “measurement of the outcome” domain. Ten studies were rated as low risk in the “selection of the reported result” domain. In the overall risk-of-bias judgment, two studies were identified as having a high risk of bias,^28^^,^^34^ nine studies were categorized as having some concerns,^29^^,^^30^^,^^31^^,^^32^^,^^33^^,^^35^^,^^36^^,^^37^^,^^38^ and one study was assessed as having a low risk of bias.^39^ See Figure 2.

### Meta-analysis results

#### Intervention effects of HIIT in college students with overweight or obesity

There was no heterogeneity in body weight (I^2^ = 0.00%, *p* = 0.77), BMI (I^2^ = 0.00%, *p* = 0.64), body fat percentage (I^2^ = 0.00%, *p* = 0.49), fat mass (I^2^ = 0.00%, *p* = 0.85), triglycerides (I^2^ = 0.00%, *p* = 0.85), and total cholesterol (I^2^ = 0.00%, *p* = 0.92); therefore, a fixed-effects model was applied for the synthesis of effect sizes in these outcomes. In contrast, there was significant heterogeneity in cardiorespiratory fitness (I^2^ = 71.31%, *p* = 0.05), and thus a random-effects model was used. In addition, to examine whether the heterogeneity in cardiorespiratory fitness was driven by any single study, we performed sensitivity analyses. Leave-one-out analyses showed that, after sequentially excluding each study, the I^2^ value for cardiorespiratory fitness remained high and the effect direction was unchanged, indicating that the heterogeneity was not attributable to a single study.

Due to low certainty of evidence, HIIT was associated with significant reductions in body weight (g = −0.63, 95% CI: −0.92 to −0.35, *p* < 0.01, *n* = 195), BMI (g = −0.71, 95% CI: −1.05 to −0.37, *p* < 0.01, *n* = 142), body fat percentage (g = −0.86, 95% CI: −1.18 to −0.55, *p* < 0.01, *n* = 165), fat mass (g = −0.63, 95% CI: −1.08 to −0.18, *p* = 0.01, *n* = 76), triglycerides (g = −0.65, 95% CI: −1.12 to −0.19, *p* = 0.01, *n* = 77), and total cholesterol (g = −0.66, 95% CI: −1.13 to −0.20, *p* < 0.01, *n* = 77).

With very low certainty of evidence, HIIT may improve cardiorespiratory fitness (g = 0.89, 95% CI: 0.11–1.67, *p* = 0.03, *n* = 99). These results should be interpreted cautiously given the overall low certainty of evidence and warrant confirmation in future high-quality trials. See Table 3, Figures 3, 4, and S1–S13.

**Table 3:** Intervention effects of HIIT in college students with overweight or obesity

Outcome	K, N	Heterogeneity	Effect model	g, 95%CI	P	Publication bias(Egger)	GRADE
Body weight	7, 195	I^2^ = 0.00%, *p* = 0.77	Fixed	−0.63, (−0.92, −0.35)	<0.01	t = 0.87, *p* = 0.42	Low
BMI	5, 142	I^2^ = 0.00%, *p* = 0.64	Fixed	−0.71, (−1.05, −0.37)	<0.01	t = 0.62, *p* = 0.58	Low
Body fat percentage	6, 165	I^2^ = 0.00%, *p* = 0.49	Fixed	−0.86, (−1.18, −0.55)	<0.01	t = 1.67, *p* = 0.17	Low
Fat mass	3, 76	I^2^ = 0.00%, *p* = 0.85	Fixed	−0.63, (−1.08, −0.18)	0.01	t = 0.36, *p* = 0.78	Low
Cardiorespiratory fitness	3, 99	I^2^ = 71.31%, *p* = 0.05	Random	0.89, (0.11, 1.67)	0.03	t = 2.44, *p* = 0.25	Very Low
TG	3, 77	I^2^ = 0.00%, *p* = 0.85	Fixed	−0.65, (−1.12, −0.19)	0.01	t = −0.48, *p* = 0.72	Low
TC	3, 77	I^2^ = 0.00%, *p* = 0.92	Fixed	−0.66, (−1.13, −0.20)	<0.01	t = −0.26, *p* = 0.84	Low

Note: BMI, body mass index; TG, triglycerides; TC, total cholesterol; K is the number of included studies; and N is sample size.

**Figure 3:**
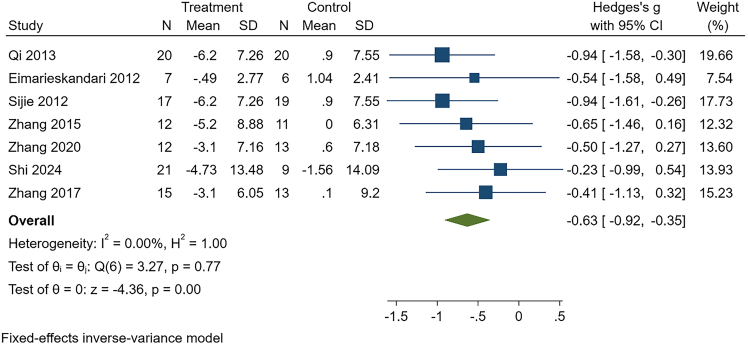
Effect of HIIT on body weight in overweight or obese college students This forest plot displays standardized mean differences (Hedges’ g) with 95% confidence intervals for each study, along with the pooled fixed-effects estimate. Negative values indicate greater reductions in body weight in the HIIT group compared with controls.

**Figure 4:**
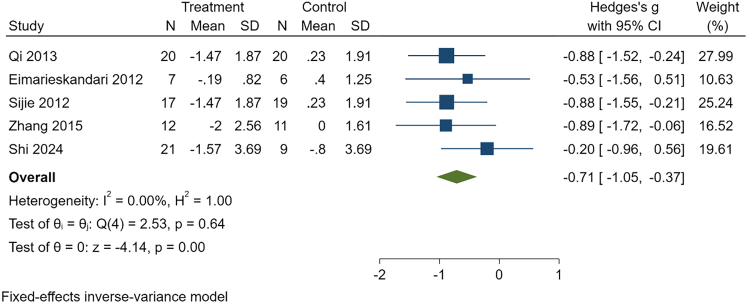
Effect of HIIT on BMI in overweight or obese college students This forest plot shows standardized mean differences (Hedges’ g) with 95% confidence intervals for individual studies and the pooled fixed-effects estimate. Negative values indicate greater reductions in BMI following HIIT compared with controls.

We conducted subgroup analyses and tests of between-group differences for the effects of HIIT on body weight and BMI. Sex, dietary monitoring, intervention duration, and training frequency were not identified as moderators (all *p* > 0.05). For body weight, significant effects were observed in the women-only subgroup (g = −0.76, *p* < 0.01), in both strata of dietary monitoring (yes: g = −0.56, *p* = 0.03; no/unclear: g = −0.67, *p* < 0.01), in the 12-week subgroup (g = −0.71, *p* < 0.01), and across both training-frequency strata (≤4 sessions per week: g = −0.45, *p* = 0.01; >4 sessions per week: g = −0.94, *p* < 0.01). For BMI, significant effects were likewise found in the women-only subgroup (g = −0.84, *p* < 0.01), in both dietary monitoring strata (yes: g = −0.75, *p* = 0.02; no/unclear: g = −0.70, *p* < 0.01), in the 12-week subgroup (g = −0.88, *p* < 0.01), and across both training-frequency strata (≤4 sessions per week: g = −0.52, *p* = 0.04; >4 sessions per week: g = −0.88, *p* < 0.01). See Table 4.

**Table 4:** Moderator analyses of the effects of HIIT on body weight and BMI in college students with overweight or obesity

Outcome	Subgroup	K	g, 95CI	P	I^2^	Tests of group differences
Body weight	Sex					Q = 1.91, *p* = 0.17
	Women-only	5	−0.76, (−1.09, −0.42)	<0.01	0.00%	
	Mixed	2	−0.32, (−0.85, 0.21)	0.23	0.00%	
	Dietary monitoring					Q = 0.11, *p* = 0.74
	Yes	3	−0.56, (−1.06, −0.07)	0.03	0.00%	
	No/unclear	4	−0.67, (−1.02, −0.32)	<0.01	2.84%	
	Intervention duration					Q = 1.13, *p* = 0.29
	8 weeks	2	−0.34, (−0.95, 0.28)	0.28	0.00%	
	12 weeks	5	−0.71, (−1.03, −0.39)	<0.01	0.00%	
	Training frequency					Q = 2.65, *p* = 0.10
	≤4 sessions/week	5	−0.45, (−0.81, −0.09)	0.01	0.00%	
	>4 sessions/week	2	−0.94, (−1.40, −0.47)	<0.01	0.00%	
BMI	Sex					Q = 2.14, *p* = 0.14
	Women-only	4	−0.84, (−1.21, −0.46)	<0.01	0.00%	
	Mixed	1	−0.20, (−0.97, 0.55)	0.60	100%	
	Dietary monitoring					Q = 0.02, *p* = 0.89
	Yes	2	−0.75, (−1.40, −0.10)	0.02	0.00%	
	No/unclear	3	−0.70, (−1.09, −0.30)	<0.01	9.93%	
	Intervention duration					Q = 2.28, *p* = 0.13
	8 weeks	2	−0.32, (−0.93, 0.30)	0.31	0.00%	
	12 weeks	3	−0.88, (−1.29, −0.48)	<0.01	0.00%	
	Training frequency					Q = 1.09, *p* = 0.30
	≤4 sessions/week	3	−0.52, (−1.01, −0.03)	0.04	0.00%	
	>4 sessions/week	2	−0.88, (−1.34, −0.42)	<0.01	0.00%	

Note: K is the number of included studies.

In linear meta-regression models using age, total number of exercise sessions, baseline BMI, and their interaction terms as predictors, none of the regression coefficients were statistically significant for either body weight or BMI (all *p* > 0.10), and all corresponding 95% CIs crossed zero. See Table 5.

**Table 5:** Meta-regression of HIIT effects on body weight and BMI in college students with overweight or obesity

Outcome	Moderators	Coefficient	SE	t	*p*	95% CI
Body weight	Age	0.11	0.21	0.50	0.64	−0.44, 0.65
	inter	−2.77	4.27	−0.65	0.55	−13.74, 8.21
	Total number of sessions	−0.02	0.01	−1.66	0.16	−0.05, 0.01
	inter	0.21	0.53	0.39	0.71	−1.14, 1.56
	Baseline BMI	−0.00	0.08	−0.04	0.97	−0.22, 0.22
	inter	−0.54	2.35	−0.23	0.83	−6.58, 5.49
BMI	Age	0.06	0.22	0.28	0.70	−0.63, 0.75
	inter	−1.95	4.40	−0.44	0.69	−15.95, 12.05
	Total number of sessions	−0.02	0.01	−1.40	0.26	−0.05, 0.02
	inter	0.07	0.59	0.12	0.91	−1.79, 1.94
	Baseline BMI	0.15	0.12	1.22	0.31	−0.24, 0.54
	inter	−4.96	3.48	−1.43	0.25	−16.03, 6.11

Publication bias was assessed for all outcomes. Egger’s tests indicated no significant bias for body weight (t = 0.87, *p* = 0.42), BMI (t = 0.62, *p* = 0.58), body fat percentage (t = 1.67, *p* = 0.17), fat mass (t = 0.36, *p* = 0.78), cardiorespiratory fitness (t = 2.44, *p* = 0.25), triglycerides (t = −0.48, *p* = 0.72), and total cholesterol (t = −0.26, *p* = 0.84). However, funnel plots for body weight, BMI, and body fat percentage showed mild asymmetry. Trim-and-fill analyses yielded only minor adjustments to the pooled effects, with the effect sizes (g) for body weight, BMI, and body fat percentage changing from −0.63 to −0.82, −0.71 to −0.83, and −0.86 to −0.81, respectively. The direction and significance of the results remained unchanged, indicating that publication bias did not materially affect the overall conclusions.

#### Intervention effects of MICT in college students with overweight or obesity

There was no heterogeneity in body weight (I^2^ = 0.00%, *p* = 0.89), BMI (I^2^ = 6.82%, *p* = 0.37), body fat percentage (I^2^ = 0.00%, *p* = 0.49), fat mass (I^2^ = 0.00%, *p* = 0.73), triglycerides (I^2^ = 0.00%, *p* = 0.47), and total cholesterol (I^2^ = 0.00%, *p* = 0.64); therefore, a fixed-effects model was applied to pool the effect sizes. There was significant heterogeneity in cardiorespiratory fitness (I^2^ = 80.27%, *p* = 0.02), so a random-effects model was employed for the effect size synthesis. Additionally, to examine whether heterogeneity in cardiorespiratory fitness was driven by any single study, we performed sensitivity analyses. Leave-one-out analyses indicated that, after sequentially excluding each study, I^2^ for cardiorespiratory fitness remained high and the direction of effect was unchanged, suggesting that the heterogeneity was not attributable to a single study.

Based on low certainty of evidence, MICT was associated with significant reductions in body weight (g = −0.45, 95% CI [−0.74 to −0.17], *p* < 0.01, *n* = 194), BMI (g = −0.74, 95% CI [−1.08 to −0.40], *p* < 0.01, *n* = 142), body fat percentage (g = −0.72, 95% CI [−1.03 to −0.41], *p* < 0.01, *n* = 163), and fat mass (g = −0.48, 95% CI [−0.93 to −0.03], *p* = 0.04, *n* = 75).

With very low certainty of evidence, MICT did not show statistically significant improvement in cardiorespiratory fitness (g = 0.64, 95% CI [−0.29 to 1.57], *p* = 0.18, *n* = 98), total cholesterol (g = −0.36, 95% CI [−0.82 to 0.09], *p* = 0.12, *n* = 77), and triglycerides (g = −0.41, 95% CI [−0.86 to 0.05], *p* = 0.08, *n* = 77).

These findings should be interpreted with caution given the overall low certainty of evidence and warrant confirmation in additional high-quality trials. See Table 6, Figures 5, 6, and S14–S26.

**Table 6:** Intervention effects of MICT in college students with overweight or obesity

Outcome	K, N	Heterogeneity	Effect model	g, 95% CI	*p*	Publication bias (Egger)	GRADE
Body weight	7, 194	I^2^ = 0.00%, *p* = 0.89	Fixed	−0.45, (−0.74, −0.17)	<0.01	t = 0.47, *p* = 0.66	Low
BMI	5, 142	I^2^ = 6.82%, *p* = 0.37	Fixed	−0.74, (−1.08, −0.40)	<0.01	t = 0.72, *p* = 0.52	Low
Body fat percentage	6, 163	I^2^ = 0.00%, *p* = 0.49	Fixed	−0.72, (−1.03, −0.41)	<0.01	t = −1.27, *p* = 0.27	Low
Fat mass	3, 75	I^2^ = 0.00%, *p* = 0.73	Fixed	−0.48, (−0.93, −0.03)	0.04	t = 0.35, *p* = 0.79	Low
Cardiorespiratory fitness	3, 98	I^2^ = 80.27%, *p* = 0.02	Random	0.64, (−0.29, 1.57)	0.18	t = 2.85, *p* = 0.21	Very Low
TG	3, 77	I^2^ = 0.00%, *p* = 0.47	Fixed	−0.36, (−0.82, 0.09)	0.12	t = −1.28, *p* = 0.42	Very Low
TC	3, 77	I^2^ = 0.00%, *p* = 0.64	Fixed	−0.41, (−0.86, 0.05)	0.08	t = −0.84, *p* = 0.55	Very Low

BMI, body mass index; TG, triglycerides; TC, total cholesterol. K is the number of included studies, and N is sample size.

**Figure 5:**
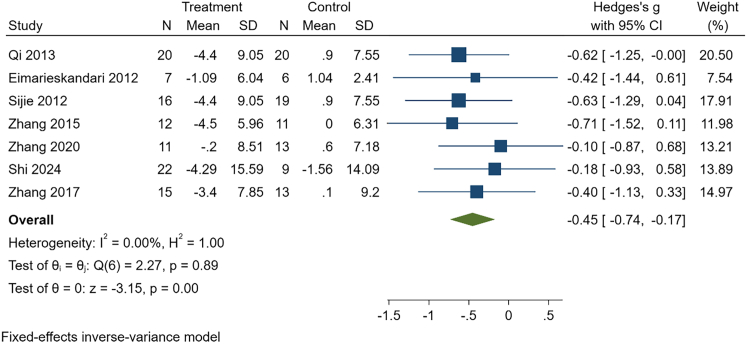
Effect of MICT on body weight in overweight or obese college students This forest plot presents standardized mean differences (Hedges’ g) with 95% confidence intervals for each study, together with the pooled fixed-effects estimate. Negative values indicate greater reduction in body weight in the MICT group relative to controls.

**Figure 6:**
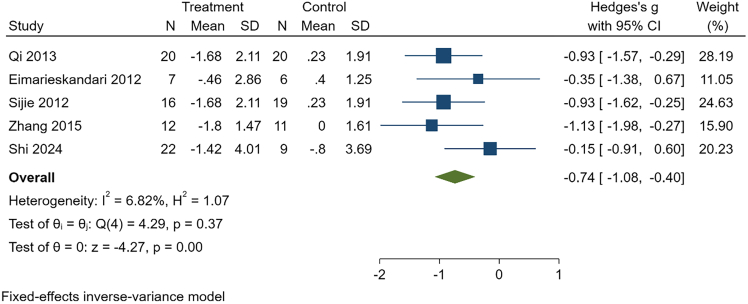
Effect of MICT on BMI in overweight or obese college students This forest plot displays standardized mean differences (Hedges’ g) with 95% confidence intervals for each study and the pooled fixed-effects estimate. Negative values indicate greater BMI reduction in the MICT group compared with controls.

We conducted subgroup analyses and the tests of between-group differences for the effects of MICT on body weight and BMI. For body weight, the sex, dietary monitoring, intervention duration, and training frequency were not identified as moderators (all *p* > 0.05). Significant effects were observed in the women-only subgroup (g = −0.52, *p* < 0.01), no/unclear dietary monitoring subgroup (g = −0.44, *p* < 0.01), 12-week subgroup (g = −0.51, *p* < 0.01), and >4 sessions per week subgroup (g = −0.63, *p* = 0.01). For BMI, the intervention duration emerged as a significant moderator: the women-only subgroup (g = −0.89, *p* < 0.01) outperformed the mixed-sex subgroup, and the 12-week subgroup (g = −0.98, *p* < 0.01) outperformed the 8-week subgroup. Significant effects were also present across both dietary monitoring strata (yes: g = −0.81, *p* = 0.02; no/unclear: g = −0.65, *p* < 0.01) and both training-frequency strata (≤4 sessions per week: g = −0.46, *p* = 0.04; >4 sessions per week: g = −0.93, *p* = 0.01). See Table 7.

**Table 7:** Moderator analyses of the effects of MICT on body weight and BMI in college students with overweight or obesity

Outcome	Subgroup	K	g, 95% CI	*p*	I^2^	Tests of group differences
Body weight	Sex					Q = 0.51, *p* = 0.47
	Women-only	5	−0.52, (−0.85, −0.19)	<0.01	0.00%	
	Mixed	2	−0.29, (−0.82, 0.23)	0.28	0.00%	
	Dietary monitoring					Q = 0.08, *p* = 0.78
	Yes	3	−0.40, (−0.89, 0.10)	0.12	0.00%	
	No/unclear	4	−0.48, (−0.83, −0.14)	0.01	0.00%	
	Intervention duration					Q = 0.49, *p* = 0.48
	8 weeks	2	−0.26, (−0.87, 0.35)	0.40	0.00%	
	12 weeks	5	−0.51, (−0.82, −0.19)	<0.01	0.00%	
	Training frequency					Q = 0.89, *p* = 0.35
	≤4 sessions/week	5	−0.35, (−0.71, 0.01)	0.06	0.00%	
	>4 sessions/week	2	−0.63, (−1.01, −0.17)	0.01	0.00%	
BMI	Sex					Q = 2.90, *p* = 0.09
	Women-only	4	−0.89, (−1.27, −0.51)	<0.01	0.00%	
	Mixed	1	−0.15, (−0.91, 0.60)	0.69	100%	
	Dietary monitoring					Q = 0.06, *p* = 0.81
	Yes	2	−0.81, (−1.47, −0.15)	0.02	23.27%	
	No/unclear	3	−0.72, (−1.11, −0.32)	<0.01	31.78%	
	Intervention duration					Q = 4.04, *p* = 0.04
	8 weeks	2	−0.22, (−0.83, 0.38)	0.47	0.00%	
	12 weeks	3	−0.98, (−1.39, −0.57)	<0.01	0.00%	
	Training frequency					Q = 1.34, *p* = 0.25
	≤4 sessions/week	3	−0.53, (−1.02, −0.03)	0.04	32.26%	
	>4 sessions/week	2	−0.93, (−1.40, −0.46)	0.01	0.00%	

Note: K is the number of included studies.

In linear meta-regression models using age, total number of exercise sessions, baseline BMI, and their interaction terms as predictors, none of the regression coefficients were statistically significant for either body weight or BMI (all *p* > 0.10), and all 95% CIs crossed zero. See Table 8.

**Table 8:** Meta-regression of MICT effects on body weight and BMI in college students with overweight or obesity

Outcome	Moderators	Coefficient	SE	t	*p*	95% CI
Body weight	Age	0.05	0.21	0.25	0.81	−0.48, 0.59
	inter	−1.51	4.23	−0.36	0.74	−12.39, 9.36
	Total number of sessions	0.00	0.08	0.02	0.99	−0.22, 0.22
	inter	−0.50	2.34	−0.21	0.84	−6.52, 5.53
	Baseline BMI	−0.01	0.01	−1.01	0.36	−0.04, 0.02
	inter	0.05	0.52	0.10	0.92	−1.29, 1.39
BMI	Age	0.10	0.22	0.48	0.67	−0.59, 0.79
	inter	−2.85	4.40	−0.65	0.56	−16.84, 11.15
	Total number of sessions	−0.02	0.01	−1.79	0.17	−0.06, 0.02
	inter	0.25	0.58	0.44	0.69	−1.60, 2.11
	Baseline BMI	0.23	0.12	1.82	0.17	−0.17, 0.62
	inter	−7.16	3.53	−2.03	0.14	−18.39, 4.07

Publication bias was assessed for all outcomes. Egger’s tests indicated no significant bias for body weight (t = 0.47, *p* = 0.66), BMI (t = 0.72, *p* = 0.52), body fat percentage (t = −1.27, *p* = 0.27), fat mass (t = 0.35, *p* = 0.79), cardiorespiratory fitness (t = 2.85, *p* = 0.21), triglycerides (t = −1.28, *p* = 0.42), and total cholesterol (t = −0.84, *p* = 0.55). However, funnel plots for body weight and body fat percentage showed mild asymmetry. Trim-and-fill analyses produced only minor adjustments to the pooled effects, with g changing from −0.45 to −0.51 for body weight and from −0.72 to −0.68 for body fat percentage. The direction and significance of the results remained unchanged, indicating that publication bias did not materially affect the overall conclusions.

#### Comparative intervention effects of HIIT versus MICT in college students with overweight or obesity

There was no heterogeneity in body weight (I^2^ = 0.00%, *p* = 1.00), BMI (I^2^ = 0.00%, *p* = 0.99), body fat percentage (I^2^ = 32.30%, *p* = 0.17), fat mass (I^2^ = 0.00%, *p* = 0.81), cardiorespiratory fitness (I^2^ = 0.00%, *p* = 0.53), systolic blood pressure (I^2^ = 0.00%, *p* = 0.69), diastolic blood pressure (I^2^ = 0.00%, *p* = 0.83), triglycerides (I^2^ = 32.60%, *p* = 0.19), total cholesterol (I^2^ = 0.00%, *p* = 0.93), high-density lipoprotein (I^2^ = 0.00%, *p* = 0.88), and low-density lipoprotein (I^2^ = 0.00%, *p* = 0.89); therefore, a fixed-effects model was used to pool the effect sizes.

With very low certainty of evidence, no statistically significant between-group differences were observed between HIIT and MICT for body weight (g = −0.10, 95% CI [−0.30 to 0.10], *p* = 0.32, *n* = 361), BMI (g = −0.00, 95% CI [−0.21 to 0.21], *p* = 1.00, *n* = 332), body fat percentage (g = −0.18, 95% CI [−0.43 to 0.07], *p* = 0.16, *n* = 238), fat mass (g = −0.30, 95% CI [−0.61 to 0.01], *p* = 0.06, *n* = 157), cardiorespiratory fitness (g = 0.24, 95% CI [−0.07 to 0.54], *p* = 0.12, *n* = 161), systolic blood pressure (g = 0.00, 95% CI [−0.38 to 0.39], *p* = 0.99, *n* = 100), diastolic blood pressure (g = 0.21, 95% CI [−0.17 to 0.60], *p* = 0.28, *n* = 100), triglycerides (g = −0.18, 95% CI [−0.45 to 0.09], *p* = 0.18, *n* = 207), total cholesterol (g = −0.11, 95% CI [−0.37 to 0.16], *p* = 0.43, *n* = 207), high-density lipoprotein (g = 0.05, 95% CI [−0.25 to 0.36], *p* = 0.74, *n* = 160), and low-density lipoprotein (g = −0.09, 95% CI [−0.39 to 0.22], *p* = 0.58, *n* = 160).

Given the very low certainty of evidence, these “no-difference” findings should not be interpreted as equivalence and warrant re-evaluation as higher-quality studies accumulate. See Table 9, Figures 7, 8, and S27–S46.

**Table 9:** Comparative effects of HIIT and MICT in college students with overweight or obesity

Outcome	K, N	Heterogeneity	Effect model	G, 95% CI	*p*	Publication bias(Egger)	GRADE
Body weight	11, 361	I^2^ = 0.00%, *p* = 1.00	Fixed	−0.10, (−0.30, 0.10)	0.32	t = 0.24, *p* = 0.82	Very low
BMI	10, 332	I^2^ = 0.00%, *p* = 0.99	Fixed	0.00, (−0.21, 0.21)	1.00	t = 0.26, *p* = 0.80	Very low
Body fat percentage	8, 238	I^2^ = 32.30%, *p* = 0.17	Fixed	−0.18, (−0.43, 0.07)	0.16	t = 2.49, *p* = 0.047	Very low
Fat mass	5, 157	I^2^ = 0.00%, *p* = 0.81	Fixed	−0.30, (−0.61, 0.01)	0.06	t = 0.59, *p* = 0.55	Very low
Cardiorespiratory fitness	5, 161	I^2^ = 0.00%, *p* = 0.53	Fixed	0.24, (−0.07, 0.54)	0.12	t = 0.87, *p* = 0.45	Very low
Systolic blood pressure	3, 100	I^2^ = 0.00%, *p* = 0.69	Fixed	0.00, (−0.38, 0.39)	0.99	t = −0.87, *p* = 0.54	Very low
Diastolic blood pressure	3, 100	I^2^ = 0.00%, *p* = 0.83	Fixed	0.21 (−0.17, 0.60)	0.28	t = −0.61, *p* = 0.65	Very low
TG	6, 207	I^2^ = 32.62%, *p* = 0.19	Fixed	−0.18, (−0.45, 0.09)	0.18	t = −1.16, *p* = 0.31	Very low
TC	6, 207	I^2^ = 0.00%, *p* = 0.93	Fixed	−0.11, (−0.37, 0.16)	0.43	t = −0.06, *p* = 0.95	Very low
High-density lipoprotein	4, 160	I^2^ = 0.00%, *p* = 0.88	Fixed	0.05, (−0.25, 0.36)	0.74	t = −0.66, *p* = 0.57	Very low
Low-density lipoprotein	4, 160	I^2^ = 0.00%, *p* = 0.89	Fixed	−0.09, (−0.39, 0.22)	0.58	t = 0.47, *p* = 0.69	Very low

BMI, body mass index; TG, triglycerides; TC, total cholesterol. K is the number of included studies, and N is sample size.

**Figure 7:**
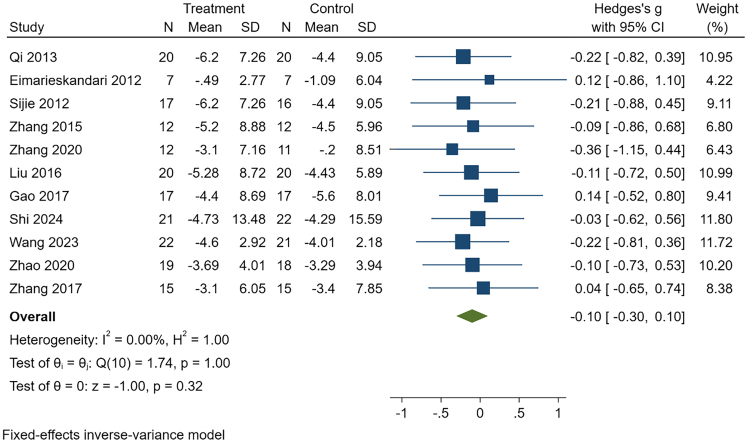
Comparative effects of HIIT versus MICT on body weight This forest plot shows standardized mean differences (Hedges’ g) with 95% confidence intervals for each study and the pooled fixed-effects estimate. Negative values indicate greater reductions in body weight with HIIT compared with MICT.

**Figure 8:**
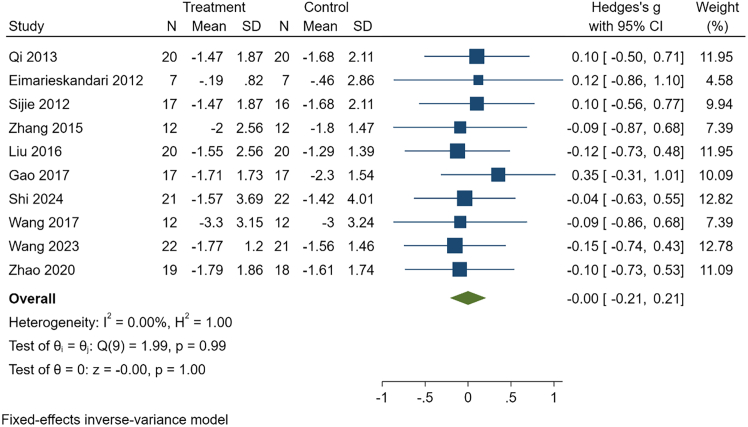
Comparative effects of HIIT versus MICT on BMI This forest plot displays standardized mean differences (Hedges’ g) with 95% confidence intervals and the pooled fixed-effects estimate. Effect sizes near zero suggest no significant difference between HIIT and MICT in reducing BMI.

We conducted subgroup analyses and the tests of between-group differences for a comparative analysis of the effects of HIIT and MICT on body weight and BMI. For body weight, the sex, dietary monitoring, intervention duration, and training frequency were not identified as moderators (all *p* > 0.05), and no statistically significant effects were detected within any subgroup (all *p* > 0.05). See Table 10.

**Table 10:** Moderator analyses of between-group differences (HIIT vs. MICT) in intervention effects on body weight and BMI among college students with overweight or obesity

Outcome	Subgroup	K	g, 95% CI	*p*	I^2^	Tests of group differences
Body weight	Sex					Q = 0.56, *p* = 0.46
	Women-only	9	−0.14, (−0.37, 0.09)	0.22	0.00	
	Mixed	2	0.0, (−0.39, 0.48)	0.84	0.00	
	Dietary monitoring					Q = 0.08, *p* = 0.78
	Yes	6	−0.07, (−0.37, 0.22)	0.63	0.00	
	No/unclear	5	−0.13, (−0.41, 0.15)	0.36	0.00	
	Intervention duration					Q = 0.23, *p* = 0.63
	8 weeks	2	0.01, (−0.49, 0.51)	0.97	0.00	
	12 weeks	9	−0.12, (−0.34, 0.10)	0.27	0.00	
	Training frequency					Q = 0.00, *p* = 0.98
	≤4 sessions/week	7	−0.10, (−0.36, 0.16)	0.43	0.00	
	>4 sessions/week	4	−0.10, (−0.42, 0.22)	0.54	0.00	
BMI	Sex					Q = 0.24, *p* = 0.63
	Women-only	7	−0.04, (−0.29, 0.22)	0.79	0.00	
	Mixed	3	0.08, (−0.30, 0.46)	0.69	0.00	
	Dietary monitoring					Q = 0.08, *p* = 0.78
	Yes	4	0.04, (−0.32, 0.40)	0.82	0.00	
	No/unclear	6	−0.02, (−0.28, 0.24)	0.87	0.00	
	Intervention duration					Q = 0.00, *p* = 0.99
	8 weeks	2	0.00, (−0.50, 0.51)	0.99	0.00	
	12 weeks	8	−0.00, (−0.23, 0.23)	0.99	0.00	
	Training frequency					Q = 0.79, *p* = 0.37
	≤4 sessions/week	6	−0.08, (−0.36, 0.20)	0.56	0.00	
	>4 sessions/week	4	0.11, (−0.21, 0.43)	0.50	0.00	

Note: K is the number of included studies.

In linear meta-regression models using age, total number of exercise sessions, baseline BMI, and their interaction terms as predictors, none of the regression coefficients were statistically significant for either body weight or BMI (all *p* > 0.10), and all 95% CIs crossed zero. See Table 11.

**Table 11:** Meta-regression of between-group differences (HIIT vs. MICT) in intervention effects on body weight and BMI among college students with overweight or obesity

Outcome	Moderators	Coefficient	SE	t	*p*	95% CI
Body weight	Age	0.10	0.12	0.85	0.42	−0.18, 0.39
	inter	−1.66	2.50	−0.89	0.40	−7.99, 3.50
	Total number of sessions	−0.00	0.01	−0.38	0.71	−0.02, 0.02
	inter	0.05	0.43	0.13	0.90	−0.91, 1.03
	Baseline BMI	0.00	0.06	0.02	0.99	−0.13, 0.13
	inter	−0.13	1.65	−0.08	0.94	−3.87, 3.60
BMI	Age	0.06	0.13	0.47	0.65	−0.23, 0.35
	inter	−1.21	2.58	−0.47	0.65	−7.17, 4.75
	Total number of sessions	0.00	0.01	0.47	0.65	−0.02, 0.02
	inter	−0.20	0.44	−0.45	0.66	−1.21, 0.81
	Baseline BMI	−0.04	0.08	−0.58	0.58	−0.22, 0.13
	inter	1.29	2.22	0.58	0.58	−3.82, 6.40

Publication bias was assessed for all outcomes. Egger’s tests indicated no significant bias for body weight (t = 0.24, *p* = 0.82), BMI (t = 0.26, *p* = 0.80), fat mass (t = 0.59, *p* = 0.55), cardiorespiratory fitness (t = 0.87, *p* = 0.45), systolic blood pressure (t = 0.87, *p* = 0.54), diastolic blood pressure (t = −0.61, *p* = 0.65), triglycerides (t = −1.16, *p* = 0.31), total cholesterol (t = −0.06, *p* = 0.95), high-density lipoprotein (t = −0.66, *p* = 0.57), and low-density lipoprotein (t = 0.47, *p* = 0.69). However, a significant bias was detected for body fat percentage (t = 2.49, *p* = 0.047). Funnel plots for fat mass, systolic blood pressure, triglycerides, and low-density lipoprotein also showed mild asymmetry. Trim-and-fill analyses produced slight adjustments to the pooled effects, with g changing from −0.30 (95% CI [−0.61, 0.01]) to −0.37 (95% CI [−0.65, −0.09]) for fat mass, from −0.19 to 0.03 for triglycerides (indicating a change in direction), from 0.00 to 0.16 for systolic blood pressure, and from −0.09 to −0.12 for low-density lipoprotein. Although the effect for fat mass became statistically significant and the direction for TG slightly reversed after adjustment, these changes were small in magnitude and do not alter the overall interpretation. The direction and significance of the other results remained unchanged, suggesting that publication bias did not materially affect the conclusions.

## Discussion

This meta-analysis systematically evaluated the intervention effects and comparative efficacy of HIIT and MICT in college students with overweight or obesity. HIIT was associated with significant improvements in body weight, BMI, body fat percentage, fat mass, cardiorespiratory fitness, triglycerides, and total cholesterol. In contrast, MICT showed significant benefits for body weight, BMI, body fat percentage, and fat mass, but did not yield significant improvements in cardiorespiratory fitness, triglycerides, or total cholesterol. Moreover, no significant between-group differences were detected between HIIT and MICT for body weight, BMI, body fat percentage, fat mass, cardiorespiratory fitness, systolic blood pressure, diastolic blood pressure, triglycerides, total cholesterol, high-density lipoprotein, or low-density lipoprotein. Nevertheless, the certainty of evidence for all outcomes was rated low to very low, primarily due to the limited number and size of the included trials and the predominance of studies judged as having some concerns or a high risk of bias. Accordingly, these findings should be interpreted with caution.

### Intervention effects of HIIT in college students with overweight or obesity

This study shows that HIIT effectively reduces body weight, BMI, body fat percentage, fat mass, triglyceride, and total cholesterol, in addition to improving cardiorespiratory fitness in college students with overweight or obesity. These findings align with prior evidence in broader populations. For example, Gawel et al. synthesized 13 studies in adults with overweight or obesity and likewise reported that HIIT reduces adiposity, enhances aerobic capacity, and improves lipid metabolism.^40^ Su et al. included 22 trials and found that HIIT significantly reduced body weight (standardized mean deviation [SMD] = −0.31, 95% CI [−0.49, −0.12], *p* < 0.01), BMI (SMD = −0.59, 95% CI [−1.04, −0.14], *p* < 0.01), body fat percentage (SMD = −0.61, 95% CI [−0.97, −0.25], *p* < 0.01), and triglyceride levels (SMD = −0.47, 95% CI [−0.78, −0.16], *p* < 0.01), while also increasing cardiorespiratory fitness (SMD = 0.97, 95% CI [0.64, 1.29], *p* < 0.01), but not the triglyceride level (SMD = −0.15, 95% CI [−0.39, 0.10], *p* = 0.24).^23^ Likewise, Wewege et al. reported that HIIT significantly reduced body fat (SMD = −0.44, 95% CI [−0.75, −0.13], *p* = 0.01) but did not lower body mass (SMD = −0.17, 95% CI [−0.36, 0.03], *p* = 0.09).^24^ Quantitatively, our pooled estimates for body weight (g = −0.63) and BMI (g = −0.71) are directionally consistent and slightly larger in magnitude than those of Wewege et al. and Su et al., indicating broadly similar intervention effects across populations. Similarly, Deng et al. included 18 studies in adolescents and found that HIIT effectively increases cardiorespiratory fitness (SMD = 0.91, 95% CI [0.66, 1.15], *p* < 0.01).^41^ Unlike these reviews, which pooled wider age ranges (Gawel: 18–50 years; Deng: 7–17 years), the present work focuses specifically on college students, a transitional group whose campus context may shape the realized effects of HIIT. Compared with non-campus populations, college students often face time constraints (course/exam cycles), social pressures, weight or appearance concerns, sleep variability, and psychological load. These factors can influence training adherence, recovery quality, and energy intake or compensation, thereby modifying both the true effect of HIIT and its detectable magnitude.^42^^,^^43^ The hallmark time efficiency of HIIT (short sessions and intermittent structure) better fits the fragmented time typical of campus life,^44^ which may partly explain the multi-outcome improvements observed in our student sample. In addition, immediate affective and self-efficacy feedback from vigorous work bouts may bolster motivation and short-term adherence.^44^ Conversely, insufficient sleep and academic stress may depress training quality, increase compensatory intake, and erode net energy deficits,^45^ underscoring the value of pairing HIIT with basic sleep or mental health support and dietary education in college settings.^18^

Regarding moderators, we found that age, baseline BMI, total number of sessions, training frequency, intervention duration, dietary control, and sex did not significantly modify HIIT’s effects on the body weight or BMI. This null pattern may reflect the restricted age or BMI range inherent to college samples and the limited number of eligible studies, which together reduced the power to detect interaction effects. Our results are not fully consistent with those of prior work. Zheng et al. reported that population characteristics (overweight vs. obesity status) did not moderate HIIT’s effect on body weight, yet it did so for cardiorespiratory fitness.^46^ A key difference is analytic: we modeled baseline BMI continuously in meta-regression, whereas Zheng et al. dichotomized BMI into the overweight and obesity categories. We also observed that significant HIIT effects on body weight and BMI were detected only in the women-only samples, likely reflecting study distribution (five female-only trials versus two mixed-sex trials). By duration, only 12-week HIIT programs reduced body weight and BMI, whereas 8-week programs did not, consistent with prior evidence. Khodadadi et al. found that interventions of >8 weeks yielded greater benefits for body fat percentage and fat mass.^47^ This pattern suggests that weight and BMI, as “slow variables,” require longer durations and higher doses to produce stable reductions; short-term energy compensation may more readily offset exercise-induced deficits, making <12-week trials less likely to detect change.^48^ Finally, we found significant improvements with both ≤4 and >4 sessions per week, consistent with evidence that ≥3 sessions per week is generally more favorable for body composition than 2 sessions per week.^47^ Notably, our ≤4 sessions per week subgroup combined trials prescribing 3, 4, or 3–4 sessions per week, which may blur finer frequency gradients. Given the limited number of trials, additional RCTs are needed to map the dose–response relation between HIIT and weight or BMI in college students with overweight or obesity.

### Intervention effects of MICT in college students with overweight or obesity

This study shows that MICT effectively reduces body weight, BMI, body fat percentage, and fat mass in college students with overweight or obesity, while not producing significant improvements in cardiorespiratory fitness and triglyceride or total cholesterol levels. These findings are not fully consistent with prior reviews. Jayedi et al. reported that MICT significantly lowers body weight (mean difference [MD] = −0.52, 95% CI [−0.61, −0.44]), waist circumference (MD = −0.56, 95% CI [−0.67, −0.45]), and body fat percentage (MD = −0.37, 95% CI [−0.43, −0.31]).^48^ Su et al., in adults with overweight or obesity, found that MICT improves body weight (SMD = 0.32, 95% CI [0.13, 0.51], *p* < 0.01), BMI (SMD = 0.73, 95% CI [0.27, 0.18], *p* < 0.01), adiposity (SMD = 0.65, 95% CI [0.32, 0.98], *p* < 0.01), and cardiorespiratory fitness (SMD = −0.69, 95% CI [−1.04, −0.34], *p* < 0.01), with a significant reduction in triglyceride levels (SMD = −0.49, 95% CI [−0.80, −0.18], *p* = 0.02).^23^ Quantitatively, our pooled estimates for body weight (g = −0.45) and BMI (g = −0.74) are directionally consistent but slightly smaller in magnitude than those reported by Jayedi et al. and Su et al., indicating that MICT produces modest yet comparable benefits across populations. Although Wewege et al. primarily examined HIIT, their results showing a reduction in body fat (SMD = −0.50, 95% CI [−0.77, −0.22], *p* < 0.01) but no significant change in body mass (SMD = −0.18, 95% CI [−0.37, 0.02], *p* = 0.08) highlight that moderate-intensity training may yield similar trends in adiposity but weaker overall weight effects.^24^ Several factors may account for the discrepancy: college samples tend to be healthier at baseline; prescriptions may involve lower intensity or dose and shorter durations; and both cardiorespiratory fitness responsiveness and lipid changes are modest and heterogeneous by nature.^49^ In addition, semester-related fluctuations (course per exam cycle) can destabilize training and sleep schedules, promoting energy compensation and adherence variability that may dilute the average effects of MICT on lipid profile and cardiorespiratory fitness. Dose–response syntheses suggest that ≥150 min per week and longer program durations are more favorable for body composition,^48^ whereas lipid improvements often require higher doses, longer interventions, or co-interventions (diet or resistance training) to manifest reliably.^48^ The phenomenon of low or no response in cardiorespiratory fitness also appears more common with moderate-intensity prescriptions, potentially reducing detectable group-level effects.^48^

Regarding moderators, age, baseline BMI, total number of sessions, training frequency, dietary control, and sex did not significantly modify MICT’s effects on body weight or BMI in the present analysis. Intervention duration emerged as a significant moderator for BMI, with effects evident only at 12 weeks. This pattern generally accords with adult-focused, dose–response evidence where the weekly volume and duration drive the magnitude of weight or BMI reductions, yet it diverges from some reports suggesting that population characteristics can modify weight-loss responses.^23^^,^^50^ For example, Zheng et al. reported moderation of MICT’s effect on body weight by population features (e.g., overweight vs. obesity status),^46^ whereas Su et al.’s broader adult samples may have encompassed greater variability in the baseline risk and dosing.^23^ We speculate that younger age, healthier baselines, insufficient dose or duration, and the small, heterogeneous effects on lipids and cardiorespiratory fitness together yield a setting in which duration shows a consistent moderating role, while other variables do not reach significance in college populations with overweight or obesity. Finally, we observed significant MICT’s effects on body weight and BMI in women-only samples; the results across training frequency and dietary monitoring strata were not uniform, likely reflecting the limited number of trials. Additional well-designed RCTs are needed to clarify the dose–response relation of MICT with weight and BMI in college students with overweight or obesity.

### Comparative effects of HIIT and MICT in college students with overweight or obesity

This study reports that HIIT and MICT do not differ significantly in their effects on body weight, BMI, body fat percentage, fat mass, cardiorespiratory fitness, systolic and diastolic blood pressure, triglycerides, total cholesterol, high-density lipoprotein, and low-density lipoprotein among college students with overweight or obesity. These findings are not entirely consistent with those reported in the literature in broader populations with overweight or obesity. Kramer et al. reported that HIIT was not uniformly superior to MICT for adiposity reduction (MD = −0.55, 95%CI [−1.42, 0.31], *p* = 0.21) yet more often shows advantages for cardiorespiratory fitness (SMD = 0.30, 95% CI [0.09, 0.52], *p* = 0.01) and selected metabolic indices such as total cholesterol (SMD = −0.42, 95% CI [−0.78, −0.05], *p* = 0.03), fasting glucose (SMD = −0.67, 95%CI [−1.07, −0.27], *p* < 0.01).^51^ Quantitatively, our pooled estimates for body weight (g = −0.10, 95% CI [−0.30, 0.10]) and TC (g = −0.11, 95% CI [−0.37, 0.16]) are smaller in magnitude than those reported by Kramer et al., suggesting that college-specific dosing and context may attenuate the advantages of HIIT observed in broader populations. Conversely, some work aligns with our results; for example, Wewege et al. reported comparable effects of HIIT and MICT in adults aged 18–45 years (body weight: SMD = 0.03, 95% CI [−0.18, 0.24], *p* = 0.79; body mass: SMD = 0.09, 95% CI [−0.10, 0.28], *p* = 0.36; and BMI: SMD = 0.09, 95% CI [−0.15, 0.32], *p* = 0.46).^24^ Our pooled comparisons for body weight (g = −0.10) and BMI (g = 0.00) closely align with these findings, further supporting the notion of comparable HIIT and MICT effects under dose-equated conditions. Importantly, the university context—characterized by time constraints, social or appearance pressures, psychological load, sleep variability, and reliance on high-energy-density takeaway foods—can jointly shape adherence and energy balance. Under dose-equated and similarly timed programs, these contextual factors may produce an “apparent overall equivalence” in practice between the two prescriptions.^42^ Accordingly, our “no difference” conclusion should be interpreted as conditional on the college setting, the doses/durations used, and the very low certainty of evidence, and must not be conflated with clinical equivalence.

We likewise found that age, baseline BMI, total number of sessions, training frequency, intervention duration, dietary control, and sex did not significantly moderate the between-group (HIIT vs. MICT) effects on body weight and BMI. This is not fully concordant with previous evidence. For example, Zheng et al. reported moderation by sex (for diastolic blood pressure, high-density lipoprotein, low-density lipoprotein), training frequency (for BMI and fat mass), age (for high-density lipoprotein), and baseline BMI (for diastolic blood pressure).^46^ These discrepancies likely reflect differences in the population composition (college students versus broader adolescent or adult samples with higher baseline risk and wider room for improvement), measurement approaches and follow-up lengths (e.g., methods for cardiorespiratory fitness assessment, limited short-term detectability, and phenotypic variability of lipid outcomes), and statistical power or heterogeneity (narrower variance and fewer trials in college samples limit subgroup or interaction detection). Thus, our conclusion of “no significant moderators” should be viewed as context bound to the prescriptions and samples in this meta-analysis, rather than generalized to all populations and dosing scenarios.

HIIT appears to offer potential benefits for reducing body weight, BMI, body fat percentage, and fat mass, as well as lowering triglycerides and total cholesterol and improving cardiorespiratory fitness in college students with overweight or obesity. MICT shows potential benefits for reducing body weight, BMI, body fat percentage, and fat mass. It should be emphasized that these conclusions may be influenced by population heterogeneity (e.g., baseline fitness levels and co-occurring psychological or metabolic characteristics) and that most studies lacked follow-up, limiting inferences about long-term maintenance and relapse risk. Accordingly, future RCTs should be larger and more rigorously designed with extended follow-up (≥6–12 months) and must systematically collect and test potential moderators, including, but not limited, to baseline cardiorespiratory fitness and habitual physical activity, dietary intake and energy balance, sleep and sedentary behavior, and academic or psychological stress. In parallel, we recommend standardizing prescription dosing and reporting adherence and adverse events to enhance comparability and external validity of the evidence base.

### Strengths and limitations

This study has several strengths: (1) This meta-analysis synthesizes current evidence on the effects of HIIT and MICT in overweight or obese college students and assesses potential differences between the two exercise approaches. (2) All studies included in this meta-analysis were RCTs, recognized as high-quality evidence, which enhances the reliability of the results. (3) The RCTs included in this meta-analysis featured at least two experimental groups (HIIT and MICT) and compared the effects of both interventions in college students with overweight or obesity, thereby minimizing methodological outliers and improving result precision.

However, this study also has certain limitations: (1) This review searched only Chinese- and English-language databases, and among English databases, only four were queried; important sources such as Scopus were not searched, which might have introduced language and publication biases. (2) This study aimed to provide a comprehensive assessment of the intervention effects of HIIT and MICT, resulting in a broad range of outcome measures. However, for specific outcomes, such as blood pressure, high-density lipoprotein, and low-density lipoprotein, the number of effect sizes was limited, and effect size pooling was not performed, which may have constrained the results. (3) The certainty of evidence across outcomes was rated low to very low; therefore, the results should be interpreted with caution.

### Future research directions

Future research should not be restricted by publication language and must include additional studies to thoroughly evaluate the intervention effects of HIIT and MICT in college students with overweight or obesity. Moreover, larger sample sizes should be utilized, and stricter, more standardized research methods (e.g., pre-registered protocols, blinded outcome assessment, and consistent dosing/reporting) need to be implemented to assess the impacts of HIIT and MICT.

## Resource availability

### Lead contact

Further information and requests for resources should be directed to and will be fulfilled by the lead contact, Sen Li (lisen@lixin.edu.cn).

### Materials availability

This study is a meta-analysis, which does not involve any reagents or new materials.

### Data and code availability

The data used for our meta-analysis were obtained from published studies, and there is no new data or codes used. The raw data in this study can be obtained on request from the corresponding author.

## Acknowledgments

This research was supported by the General Project of the National Social Science Fund of China (Grant No. 23BTY078, 2023) and the Shanghai Education Science Research Project (Grant No. C2023112).

## Author contributions

C.Z.C. wrote the manuscript. C.W.Y., S.L., and C.X.W. extracted data, analyzed data, and produced pictures. C.X.W. constructed the framework of the study and revised the manuscript.

## Declaration of interests

The authors declare no competing interests.

## STAR★Methods

### Key resources table

**Table:** 

REAGENT or RESOURCE	SOURCE	IDENTIFIER
**Software and algorithms**		

Software: EndNote X9	Clarivate Analytics	https://endnote.com/downloads
Software: Stata 17	StataCorp	https://www.stata.com/products

**Deposited data**		

Database: PROSPERO	PROSPERO	https://www.crd.york.ac.uk/prospero
Database: PubMed	Published literature	https://www.ncbi.nlm.nih.gov/pubmed
Database: Cochrane Library	Published literature	https://www.cochranelibrary.com
Database: Embase	Published literature	https://www.embase.com
Database: Web of Science	Published literature	https://www.webofscience.com
Database: China National Knowledge Infrastructure (CNKI)	Published literature	https://www.cnki.net
Data and Code Availability	This study	No new data or code generated

### Experimental model and study participant details

Not applicable. This study is a systematic review and meta-analysis and does not involve human participants, animals, or in-vitro experimental models.

### Method details

#### Protocol and registration

This study was conducted strictly following the Preferred Reporting Items for Systematic Reviews and Meta-Analyses (PRISMA) guidelines and steps.^52^ The study protocol has been registered with the International Prospective Register of Systematic Reviews (PROSPERO) under the registration number CRD420250655528. As shown in supplemental information, the PRISMA 2020 checklist is included.

#### Inclusion and exclusion criteria

The inclusion and exclusion criteria for this study were based on the population (P), intervention (I), comparison (C), outcome (O), and study design (S). P: College students aged 18 to 26 years with a BMI of 24 kg/m^2^ or higher. A BMI of 24 kg/m^2^ to less than 28 kg/m^2^ is classified as overweight, while a BMI of 28 kg/m^2^ or higher is classified as obesity.^53^ I: Trials including at least two intervention arms-one performing HIIT and the other MICT-were eligible. Studies had to permit extraction of key exercise variables (e.g., exercise intensity, intervention duration, and training frequency), with a minimum intervention period of ≥ 6 weeks.^54^ C: If a control group is included, no form of exercise will be administered to the control group. O: Primary outcomes were body weight and BMI. Secondary outcomes included body fat percentage, fat mass, cardiorespiratory fitness, blood pressure, high-density lipoprotein, low-density lipoprotein, total cholesterol (TC), and triglycerides (TG). All outcomes had to be assessed using comparable measurement tools, and at least one of the above outcomes had to be reported in a form permitting effect-size computation. Each study arm was required to report pre- and post-intervention means and standard deviations (or equivalent statistics allowing derivation). Effect sizes were computed from change scores (post − pre). S: Only RCTs were included in this study.Exclusion Criteria: Studies published in languages other than Chinese or English. Non-RCTs and acute, single-bout exercise studies. Trials combining exercise with other interventions when the exercise effect could not be isolated. Studies in which data could not be obtained despite efforts to email the original authors. Studies whose designs did not satisfy the inclusion criteria, such as those consisting of only one type of exercise in the experimental group.

#### Search strategy

A computer-based search was conducted across five bilingual databases: China National Knowledge Infrastructure (CNKI), PubMed, Web of Science (WOS), Embase, and the Cochrane Library, focusing on RCTs involving HIIT and MICT interventions for college students with overweight or obesity. The search was conducted up to February 21, 2025. The search strategy employed both subject and free terms, combined using AND and OR. Search terms included “high-intensity interval training,” “aerobic interval training,” “high-intensity intermittent training,” “moderate intensity continuous training,” “college students,” adolescents, obesity, overweight, among others. Additionally, we screened the reference lists of relevant systematic reviews and meta-analyses to minimize the risk of omission, as detailed in Table S1 of the supplemental information.

#### Literature screening process

Two researchers imported the retrieved literature into EndNote X9 and eliminated duplicates. They initially screened the articles by reading the titles and abstracts to exclude irrelevant ones. The full-text versions of the remaining articles were downloaded, and full-text screening was performed based on the inclusion and exclusion criteria. If there was a disagreement between the two researchers, a third researcher was consulted until a consensus was reached.

#### Data extraction and coding

Two researchers utilized a pre-designed table to gather information from the selected studies. The collected data included fundamental details (authors, year, sample size), characteristics of the interventions (type of exercise, intervention duration), and outcome measures. In cases of disagreement, a third researcher was included in discussions to reach a consensus.

To maintain inferential clarity and minimize multiplicity, we restricted prespecified subgroup analyses and meta-regressions to body weight and BMI. Dietary monitoring was coded as yes versus no/unclear; intervention duration as 8 weeks versus 12 weeks; training frequency as ≤4 sessions/week versus >4 sessions/week; and sex as women-only versus mixed. In addition, we performed meta-regression on mean age and the total number of exercise sessions.

#### Risk of bias and evidence level evaluation

Since all the studies included were randomized controlled trials, the risk of bias was assessed using the Cochrane Risk of Bias Assessment Tool for Randomized Controlled Trials (RoB 2.0).^55^ RoB 2.0 evaluates bias across five domains: “Randomization process,” “Deviations from the intended interventions,” “Missing outcome data,” “Measurement of the outcome,” and “Selection of the reported result.” Risks are categorized as “Low risk of bias,” “Some concerns,” and “High risk of bias.”

To help explain sources of inconsistency, we operationalized decision rules for two domains—deviations from the intended interventions and selection of the reported result: Deviations from the intended interventions. We focused on: (a) Adherence and dose fidelity: attendance ≥80% with intensity compliance ≥70% was considered adequate; attendance 60–79% indicated some concerns; attendance <60% was judged high risk. (b) Co-interventions and contamination: for example, high risk was assigned when ≥20% of the control group engaged in regular exercise (or when there were clear imbalances between groups in diet or medication). (c) Analysis set and attrition: low risk required intention-to-treat analysis with <10% loss to follow-up; high risk was assigned when only per-protocol analyses were reported or when differential attrition reached ≥20%. Selection of the reported result. We examined:(1) whether outcome assessors were blinded; (2) the comparability and validity of measurement tools/protocols (e.g., mixing cardiopulmonary exercise testing with field tests for cardiorespiratory fitness, or mixing Dual-Energy X-ray Absorptiometry with Bioelectrical Impedance Analysis for body composition without stratification or sensitivity analyses was judged high risk); (3) the consistency of assessment time points (e.g., evaluations conducted >4 weeks after intervention completion or systematically different assessment timings between groups were judged some concerns or high risk, as appropriate).

The evidence level was assessed using GRADEpro, which considers five aspects: study limitations, inconsistencies, indirectness, imprecision, and publication bias.^56^ For imprecision, we adopted a minimally contextualized approach: we downgraded by one level when the 95% confidence interval (CI) around the pooled effect crossed the line of no effect (0 for continuous outcomes); we downgraded by two levels when the 95% CI was very wide such that the direction of effect was uncertain, or when the optimal information size was not met owing to few studies and a small total sample. When fewer than 10 studies were available, funnel plots and Egger’s test were deemed underpowered and were not treated as mandatory grounds for downgrading, unless trial registries or grey literature provided credible indications of missing studies. See Table S2.

### Quantification and statistical analysis

The data synthesis was conducted using Stata 17, which facilitated effect size pooling, forest plot creation, and publication bias tests. Effect size was represented by Hedges’ g (g), with statistical significance at P < 0.05, and 95%CI were calculated. Heterogeneity was assessed using the Q-test P-value and I^2^. A fixed-effects model was employed when I^2^ < 50% and P < 0.1; otherwise, a random-effects model was utilized. When heterogeneity was substantial, we conducted sensitivity analyses (leave-one-out) to examine whether any single study disproportionately influenced the results. Publication bias was probed by funnel plots and Egger’s regression test; when asymmetry suggested potential bias, we applied the trim-and-fill procedure to estimate the impact of missing studies.

## References

[1] Rajaram S., Wojcik R., Moore C., Ortiz de Lejarazu R., de Lusignan S., Montomoli E., Rossi A., Pérez-Rubio A., Trilla A., Baldo V. (2020). The impact of candidate influenza virus and egg-based manufacture on vaccine effectiveness: Literature review and expert consensus. Vaccine.

[2] Obirikorang C., Adu E.A., Anto E.O., Afum-Adjei Awuah A., Fynn A.N.B., Osei-Somuah G., Ansong P.N., Boakye A.O., Ofori-Boadu I., Obirikorang Y. (2024). Prevalence and risk factors of obesity among undergraduate student population in Ghana: an evaluation study of body composition indices. BMC Public Health.

[3] Poobalan A., Aucott L. (2016). Obesity Among Young Adults in Developing Countries: A Systematic Overview. Curr. Obes. Rep..

[4] Ortega R., Grandes G., Sanchez A., Montoya I., Torcal J., PEPAF group (2019). Cardiorespiratory fitness and development of abdominal obesity. Prev. Med..

[5] Racil G., Chelly M.S., Coquart J., Padulo J., Teodor D.F., Russo L. (2023). Long- and Short-Term High-Intensity Interval Training on Lipid Profile and Cardiovascular Disorders in Obese Male Adolescents. Children.

[6] Sánchez-Delgado A., Pérez-Bey A., Izquierdo-Gómez R., Jimenez-Iglesias J., Marcos A., Gómez-Martínez S., Girela-Rejón M.J., Veiga O.L., Castro-Piñero J. (2023). Fitness, body composition, and metabolic risk scores in children and adolescents: the UP&DOWN study. Eur. J. Pediatr..

[7] Ng M., Fleming T., Robinson M., Thomson B., Graetz N., Margono C., Mullany E.C., Biryukov S., Abbafati C., Abera S.F. (2014). Global, regional, and national prevalence of overweight and obesity in children and adults during 1980-2013: a systematic analysis for the Global Burden of Disease Study 2013. Lancet.

[8] Ling J., Chen S., Zahry N.R., Kao T.S.A. (2023). Economic burden of childhood overweight and obesity: A systematic review and meta-analysis. Obes. Rev..

[9] Gawlik K., Melnyk B.M., Tan A., Amaya M. (2019). Heart checks in college-aged students link poor sleep to cardiovascular risk. J. Am. Coll. Health.

[10] Shafiee A., Nakhaee Z., Bahri R.A., Amini M.J., Salehi A., Jafarabady K., Seighali N., Rashidian P., Fathi H., Esmaeilpur Abianeh F. (2024). Global prevalence of obesity and overweight among medical students: a systematic review and meta-analysis. BMC Public Health.

[11] Kelsey M.M., Zeitler P.S. (2016). Insulin Resistance of Puberty. Curr. Diab. Rep..

[12] Moran A., Jacobs D.R., Steinberger J., Steffen L.M., Pankow J.S., Hong C.-P., Sinaiko A.R. (2008). Changes in Insulin Resistance and Cardiovascular Risk During Adolescence. Circulation.

[13] Hagenauer M.H., Perryman J.I., Lee T.M., Carskadon M.A. (2009). Adolescent changes in the homeostatic and circadian regulation of sleep. Dev. Neurosci..

[14] Snedden T.R., Scerpella J., Kliethermes S.A., Norman R.S., Blyholder L., Sanfilippo J., McGuine T.A., Heiderscheit B. (2019). Sport and Physical Activity Level Impacts Health-Related Quality of Life Among Collegiate Students. Am. J. Health Promot..

[15] Brown C.E.B., Richardson K., Halil-Pizzirani B., Atkins L., Yücel M., Segrave R.A. (2024). Key influences on university students’ physical activity: a systematic review using the Theoretical Domains Framework and the COM-B model of human behaviour. BMC Public Health.

[16] Gardani M., Bradford D.R.R., Russell K., Allan S., Beattie L., Ellis J.G., Akram U. (2022). A systematic review and meta-analysis of poor sleep, insomnia symptoms and stress in undergraduate students. Sleep Med. Rev..

[17] Levine J.A. (2004). Nonexercise activity thermogenesis (NEAT): environment and biology. Am. J. Physiol. Endocrinol. Metab..

[18] Donnelly J.E., Blair S.N., Jakicic J.M., Manore M.M., Rankin J.W., Smith B.K., American College of Sports Medicine (2009). American College of Sports Medicine Position Stand. Appropriate physical activity intervention strategies for weight loss and prevention of weight regain for adults. Med. Sci. Sports Exerc..

[19] Vella C.A., Taylor K., Drummer D. (2017). High-intensity interval and moderate-intensity continuous training elicit similar enjoyment and adherence levels in overweight and obese adults. Eur. J. Sport Sci..

[20] Després J.P., Lemieux I. (2006). Abdominal obesity and metabolic syndrome. Nature.

[21] Higgins S., Fedewa M.V., Hathaway E.D., Schmidt M.D., Evans E.M. (2016). Sprint interval and moderate-intensity cycling training differentially affect adiposity and aerobic capacity in overweight young-adult women. Appl. Physiol. Nutr. Metab..

[22] Maillard F., Pereira B., Boisseau N. (2018). Effect of High-Intensity Interval Training on Total, Abdominal and Visceral Fat Mass: A Meta-Analysis. Sports Med..

[23] Su L., Fu J., Sun S., Zhao G., Cheng W., Dou C., Quan M. (2019). Effects of HIIT and MICT on cardiovascular risk factors in adults with overweight and/or obesity: A meta-analysis. PLoS One.

[24] Wewege M., van den Berg R., Ward R.E., Keech A. (2017). The effects of high-intensity interval training vs. moderate-intensity continuous training on body composition in overweight and obese adults: a systematic review and meta-analysis. Obes. Rev..

[25] Chen X., He H., Xie K., Zhang L., Cao C. (2024). Effects of various exercise types on visceral adipose tissue in individuals with overweight and obesity: A systematic review and network meta-analysis of 84 randomized controlled trials. Obes. Rev..

[26] Hu M., Kong Z., Sun S., Zou L., Shi Q., Chow B.C., Nie J. (2021). Interval training causes the same exercise enjoyment as moderate-intensity training to improve cardiorespiratory fitness and body composition in young Chinese women with elevated BMI. J. Sports Sci..

[27] Zhu X., Jiao J., Liu Y., Li H., Zhang H. (2024). The Release of Lipolytic Hormones during Various High-Intensity Interval and Moderate-Intensity Continuous Training Regimens and Their Effects on Fat Loss. J. Sports Sci. Med..

[28] Eimarieskandari R., Zilaeibouri S., Zilaeibouri M., Ahangarpour A. (2012). Comparing two modes of exercise training with different intensity on body composition in obese young girls. Sci. Mov. Health.

[29] Sijie T., Hainai Y., Fengying Y., Jianxiong W. (2012). High intensity interval exercise training in overweight young women. J. Sports Med. Phys. Fitness.

[30] Zhang H., Tong T.K., Qiu W., Wang J., He Y. (2015). Effect of High-Intensity Interval Training Protocol on Abdominal Fat Reduction in Overweight Chinese Women: A Randomized Controlled Trial. Kinesiology.

[31] Zhang H., Tong T.K., Qiu W., Zhang X., Zhou S., Liu Y., He Y. (2017). Comparable effects of high-intensity interval training and prolonged continuous exercise training on abdominal visceral fat reduction in obese young women. J. Diabetes Res..

[32] Zhang H., Tong T.K., Kong Z., Shi Q., Liu Y., Nie J. (2021). Exercise training-induced visceral fat loss in obese women: The role of training intensity and modality. Scand. J. Med. Sci. Sports.

[33] Liu H., Liu Z., Wang C. (2016). Effect of high intensity interval training on lose weight in obese young women. J. Shandong Sport Univ..

[34] Qi Y., Huang J., Tan S. (2013). Comparison of Weight Loss Effects Carried out by HIIT and Continuous Aerobic Exercise of Female Obese College Students. China Sport Sci. Technol..

[35] Zhao J., Liang J., Hao L. (2020). Impact of Moderate-to-High Intensity Exercise on Body Composition and Cardiovascular Parameters in Obese Female College Students. Chin. J. School Health.

[36] Shi W., Xie J., He Y., Li X., Tang D. (2024). Effects of High-Intensity Interval Training and Moderate-Intensity Continuous Training on Vascular Endothelial Function and Wall Shear Stress in Obese Male College Students. China Sport Sci. Technol..

[37] Gao Y., Wang G., Yang W., Qiao X. (2017). Effects of High-Intensity Interval Training and Aerobic Exercise on Lipid Metabolism and Chronic Inflammation in Obese Young Adults. Chin. J. Sports Med..

[38] Wang P., Liu B., Liu Y., Jiang D. (2023). Effects of Exercise Combined with Dietary Intervention on Body Composition, Lipid Metabolism, and Gut Microbiota in Obese Female College Students with Non-Alcoholic Fatty Liver Disease. Chin. J. School Health.

[39] Wang H., Yan F., Zhang L. (2017). Effects of High-Intensity Interval Training and Moderate-Intensity Continuous Training on Body Mass Index, Blood Pressure, and Cardiorespiratory Fitness in Obese College Students. Chin. J. Appl. Physiol..

[40] Gaweł E., Hall B., Siatkowski S., Grabowska A., Zwierzchowska A. (2024). The Combined Effects of High-Intensity Interval Exercise Training and Dietary Supplementation on Reduction of Body Fat in Adults with Overweight and Obesity: A Systematic Review. Nutrients.

[41] Deng Y., Wang X. (2024). Effect of high-intensity interval training on cardiorespiratory in children and adolescents with overweight or obesity: a meta-analysis of randomized controlled trials. Front. Public Health.

[42] Brown C.E.B., Richardson K., Halil-Pizzirani B., Atkins L., Yücel M., Segrave R.A. (2024). Key influences on university students' physical activity: a systematic review using the Theoretical Domains Framework and the COM-B model of human behaviour. BMC Public Health.

[43] Ferreira Silva R.M., Mendonça C.R., Azevedo V.D., Raoof Memon A., Noll P.R.E.S., Noll M. (2022). Barriers to high school and university students' physical activity: A systematic review. PLoS One.

[44] Thum J.S., Parsons G., Whittle T., Astorino T.A. (2017). High-Intensity Interval Training Elicits Higher Enjoyment than Moderate Intensity Continuous Exercise. PLoS One.

[45] Fullagar H.H.K., Skorski S., Duffield R., Hammes D., Coutts A.J., Meyer T. (2015). Sleep and athletic performance: the effects of sleep loss on exercise performance, and physiological and cognitive responses to exercise. Sports Med..

[46] Zheng W., Yin M., Guo Y., Liu H., Sun J., Zhu A., Zhong Y., Xu K., Li H., Piao S. (2025). Effects and moderator of high-intensity interval training and moderate-intensity continuous training among children and adolescents with overweight or obese: a systematic review and meta-analysis. Front. Physiol..

[47] Khodadadi F., Bagheri R., Negaresh R., Moradi S., Nordvall M., Camera D.M., Wong A., Suzuki K. (2023). The Effect of High-Intensity Interval Training Type on Body Fat Percentage, Fat and Fat-Free Mass: A Systematic Review and Meta-Analysis of Randomized Clinical Trials. J. Clin. Med..

[48] Jayedi A., Soltani S., Emadi A., Zargar M.S., Najafi A. (2024). Aerobic Exercise and Weight Loss in Adults: A Systematic Review and Dose-Response Meta-Analysis. JAMA Netw. Open.

[49] Smart N.A., Downes D., van der Touw T., Hada S., Dieberg G., Pearson M.J., Wolden M., King N., Goodman S.P.J. (2025). The Effect of Exercise Training on Blood Lipids: A Systematic Review and Meta-analysis. Sports Med..

[50] Jayedi A., Soltani S., Emadi A., Zargar M.-S., Najafi A. (2024). Aerobic Exercise and Weight Loss in Adults: A Systematic Review and Dose-Response Meta-Analysis. JAMA Netw. Open.

[51] Kramer A.M., Martins J.B., de Oliveira P.C., Lehnen A.M., Waclawovsky G. (2023). High-intensity interval training is not superior to continuous aerobic training in reducing body fat: A systematic review and meta-analysis of randomized clinical trials. J. Exerc. Sci. Fit..

[52] Page M.J., McKenzie J.E., Bossuyt P.M., Boutron I., Hoffmann T.C., Mulrow C.D., Shamseer L., Tetzlaff J.M., Akl E.A., Brennan S.E. (2021). The PRISMA 2020 statement: an updated guideline for reporting systematic reviews. BMJ.

[53] Cao M., Yang B., Tang Y., Wang C., Yin L. (2024). Effects of low-volume functional and running high-intensity interval training on physical fitness in young adults with overweight/obesity. Front. Physiol..

[54] Tataryn N., Simas V., Catterall T., Furness J., Keogh J.W.L. (2021). Posterior-Chain Resistance Training Compared to General Exercise and Walking Programmes for the Treatment of Chronic Low Back Pain in the General Population: A Systematic Review and Meta-Analysis. Sports Med. Open.

[55] Crocker T.F., Lam N., Jordão M., Brundle C., Prescott M., Forster A., Ensor J., Gladman J., Clegg A. (2023). Risk-of-bias assessment using Cochrane's revised tool for randomized trials (RoB 2) was useful but challenging and resource-intensive: observations from a systematic review. J. Clin. Epidemiol..

[56] Marino L., Lancellotta V., Franco P., Meattini I., Meduri B., Bernini M., Fabi A., Corvò R., Magrini S.M., Pappagallo G.L. (2021). Loco-regional adjuvant radiation therapy in breast cancer patients with positive axillary lymph-nodes at diagnosis (CN2) undergoing preoperative chemotherapy and with complete pathological lymph-nodes response. Development of GRADE (Grades of recommendation, assessment, Development and Evaluation) recommendation by the Italian Association of radiation therapy and Clinical Oncology (AIRO). Breast.

