# Supplementary material for: Effects of high-intensity interval and moderate-intensity continuous training on overweight or obese college students: A systematic review and meta-analysis
Source: iScience. 2025 Dec 6;29(1):114361. doi: 10.1016/j.isci.2025.114361 (PMC12775873; doi:10.1016/j.isci.2025.114361)
Supplement: Document S1. Figures S1–S46 and Tables S1 and S2 [file mmc1.pdf]

iScience, Volume *29*

Supplemental information

Effects of high-intensity interval and moderate-intensity continuous training on overweight or obese college students: A systematic review and meta-analysis

Changzhou Chen, Chuanwen Yu, and Sen Li

HIIT vs Control

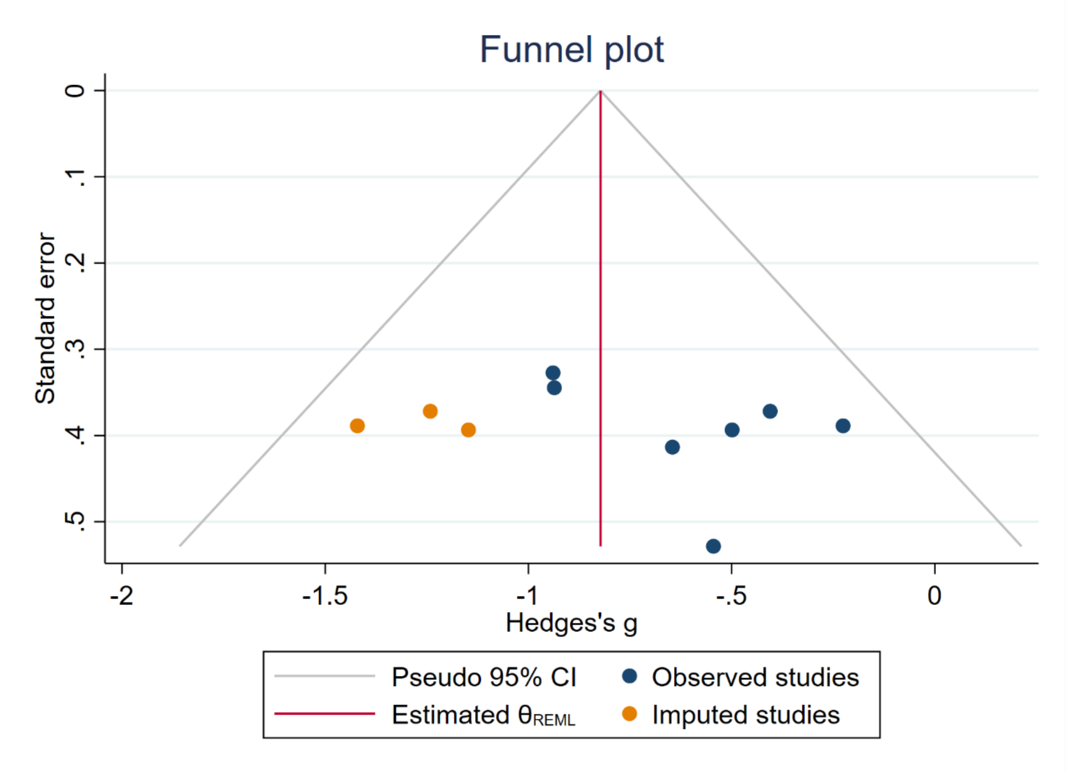

Figure S1. Funnel plot of the effect of HIIT on body weight in college students with overweight or obesity.

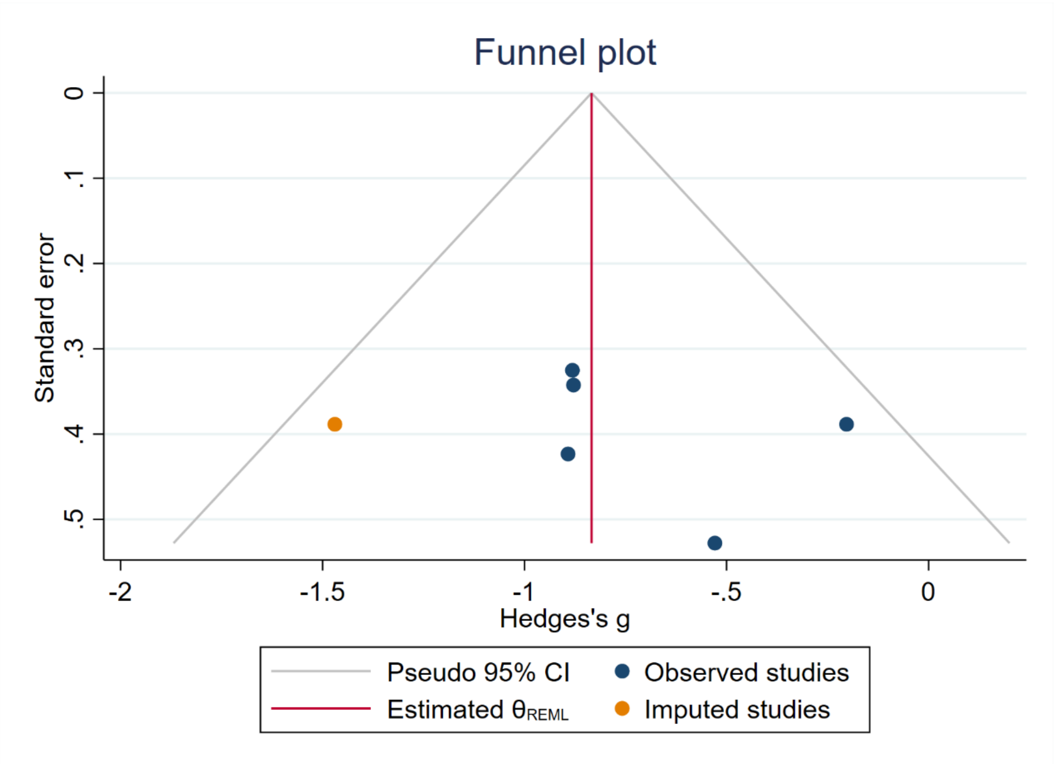

Figure S2. Funnel plot of the effect of HIIT on BMI in college students with overweight or obesity.

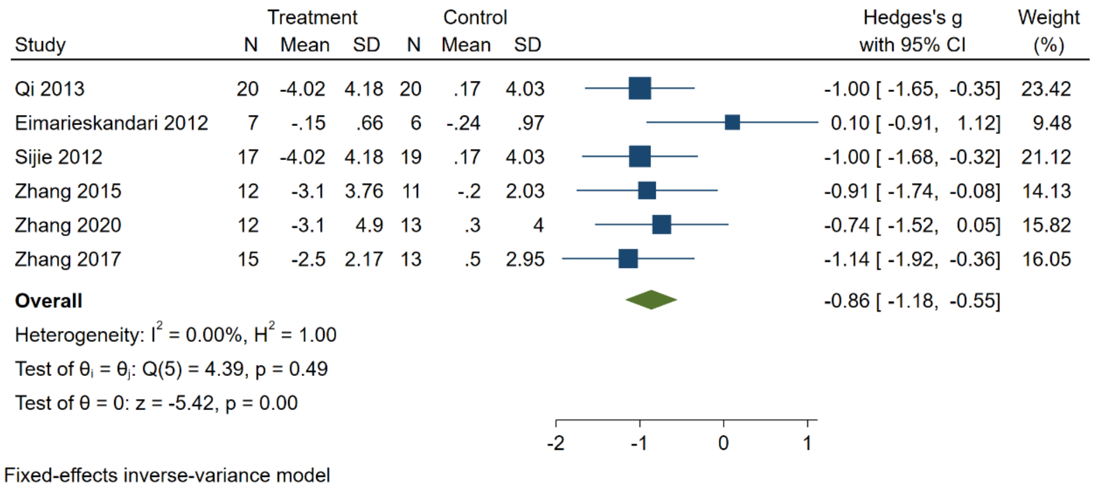

Figure S3. Forest plot of the effect of HIIT on body fat percentage in college students with overweight or obesity.

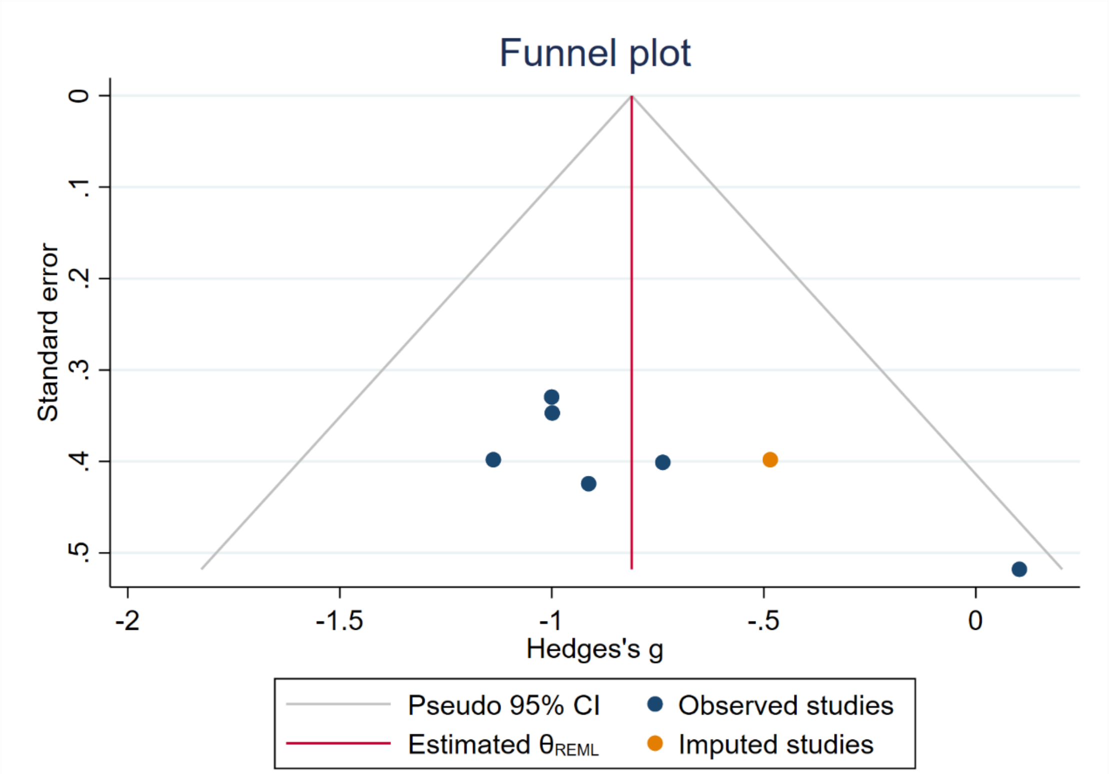

Figure S4. Funnel plot of the effect of HIIT on body fat percentage in college students with overweight or obesity

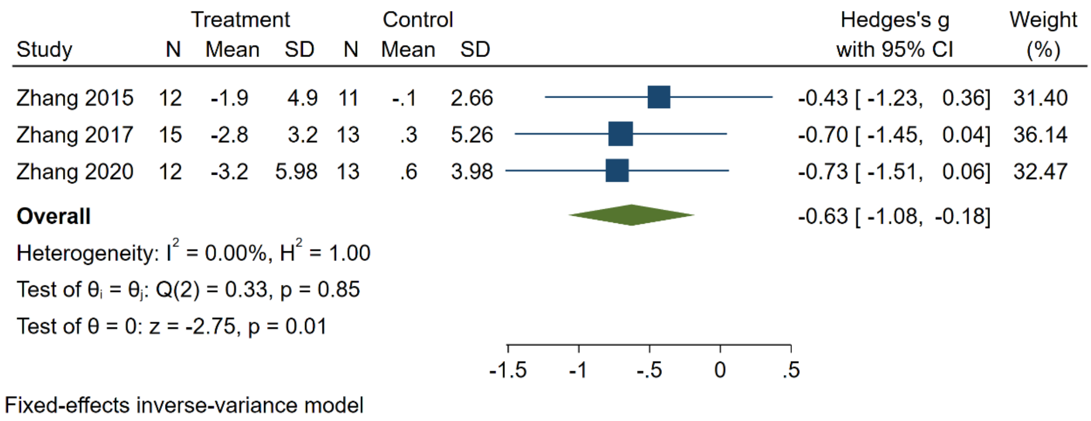

Figure S5. Forest plot of the effect of HIIT on fat mass in college students with overweight or obesity.

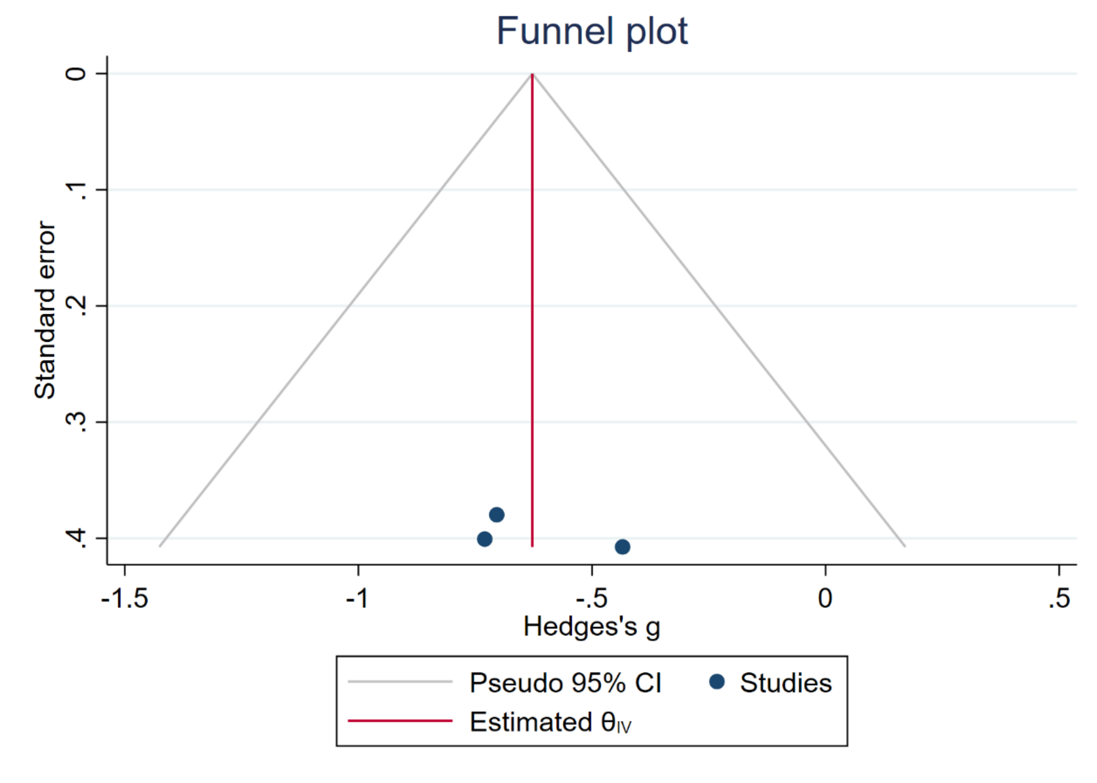

Figure S6. Funnel plot of the effect of HIIT on fat mass in college students with overweight or obesity.

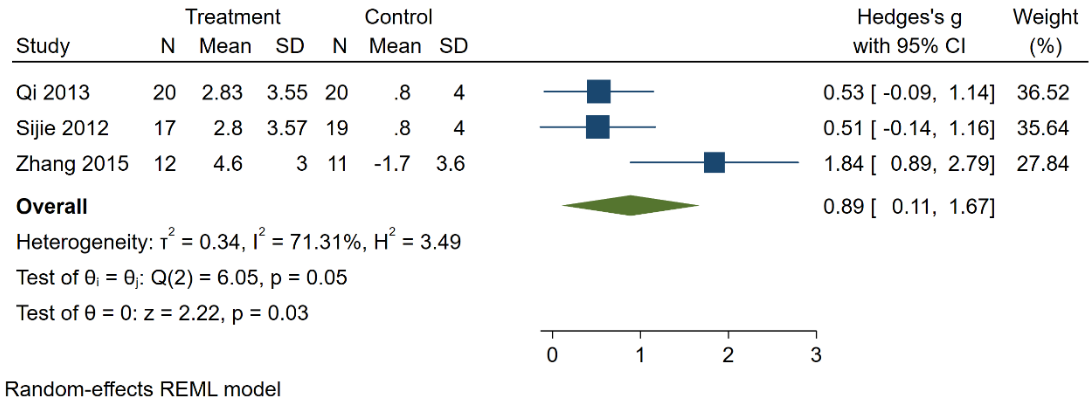

Figure S7. Forest plot of the effect of HIIT on cardiorespiratory fitness in college students with overweight or obesity.

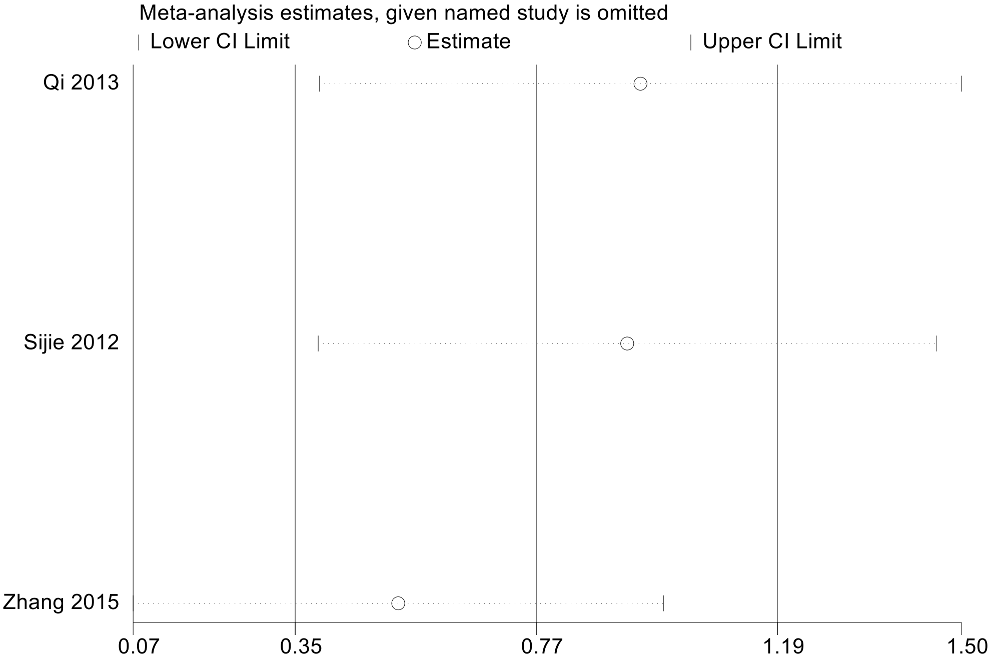

Figure S8. Sensitivity analysis of the effect of HIIT on cardiorespiratory fitness in college students with overweight or obesity.

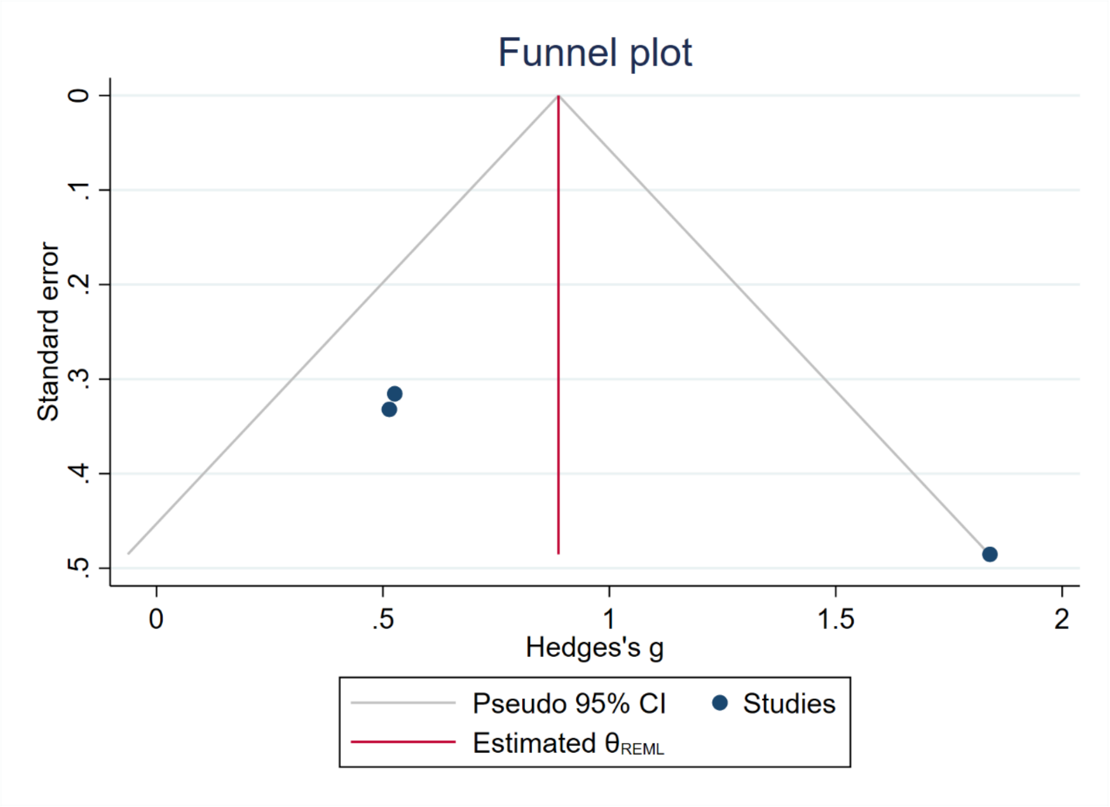

Figure S9. Funnel plot of the effect of HIIT on cardiorespiratory fitness in college students with overweight or obesity.

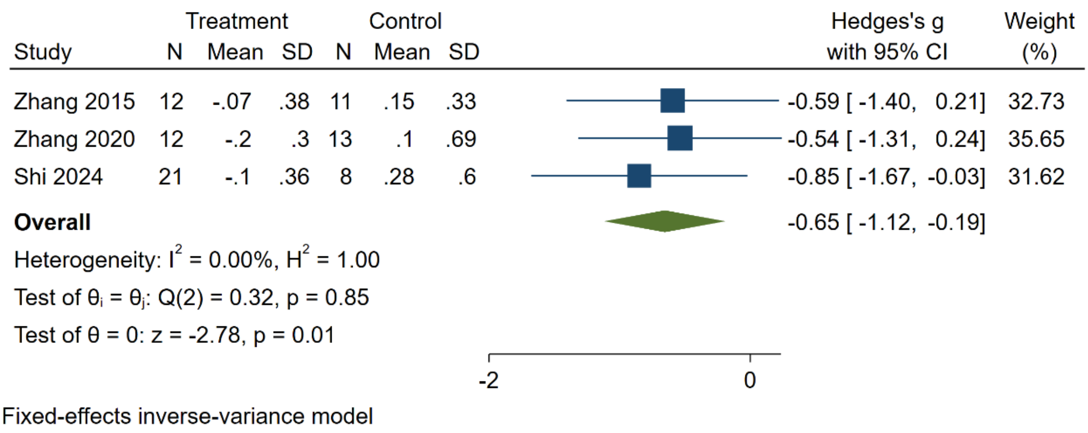

Figure S10. Forest plot of the effect of HIIT on TG in college students with overweight or obesity.

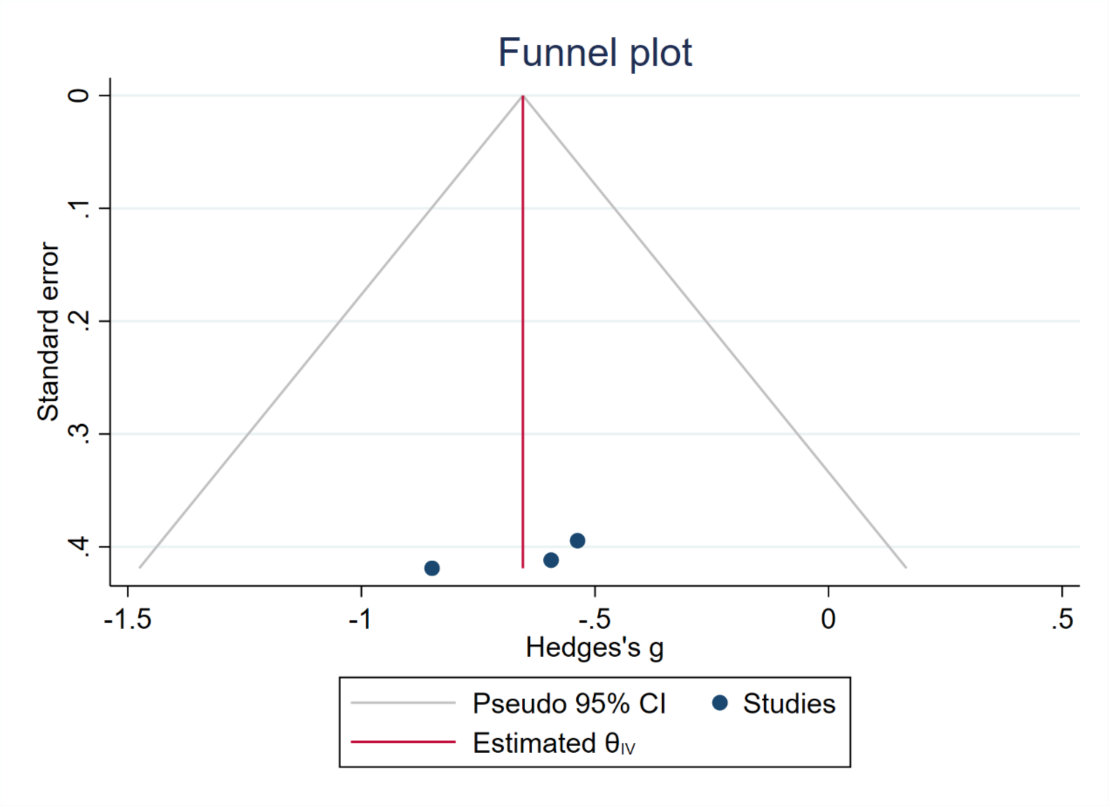

Figure S11. Funnel plot of the effect of HIIT on TG in college students with overweight or obesity.

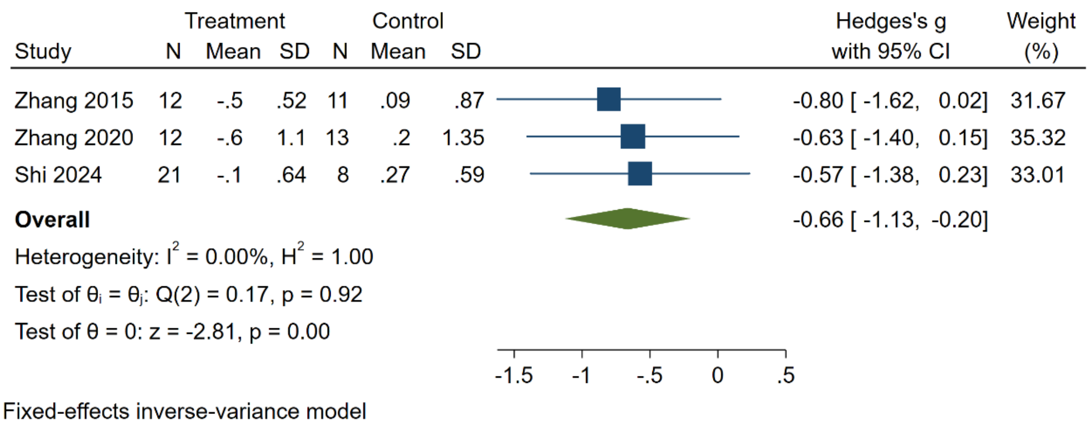

Figure S12. Forest plot of the effect of HIIT on TC in college students with overweight or obesity.

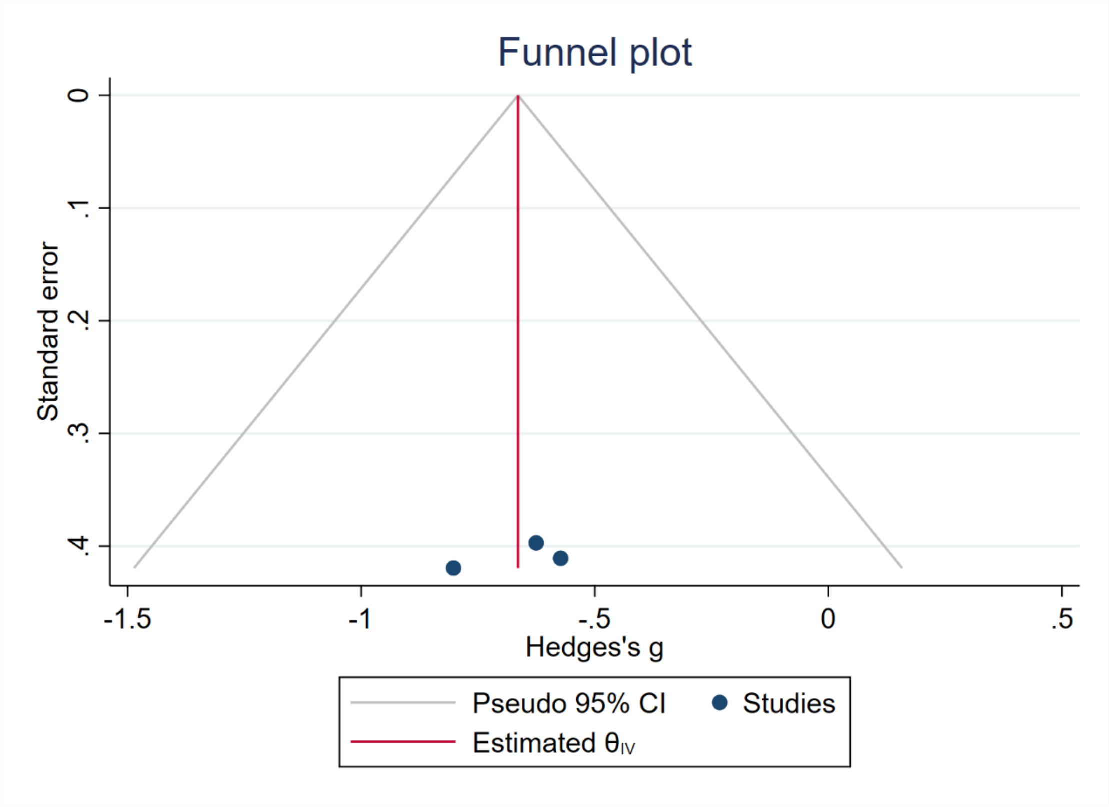

Figure S13. Funnel plot of the effect of HIIT on TC in college students with overweight or obesity.

MICT vs Control

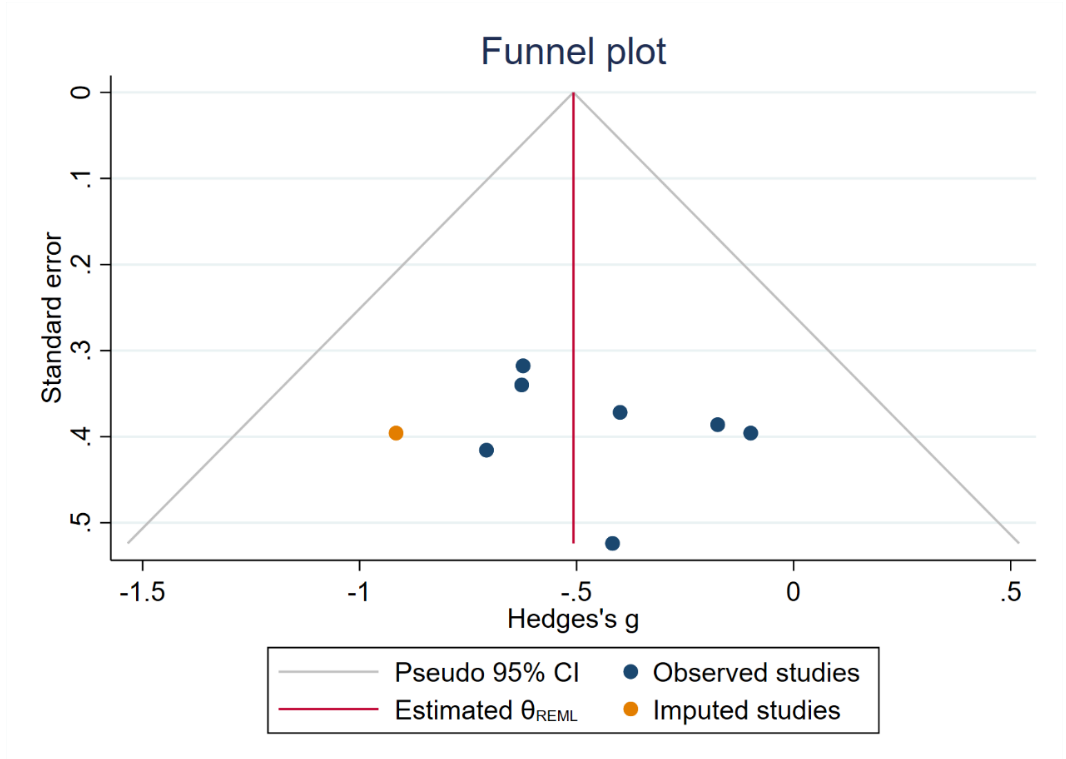

Figure S14. Funnel plot of the effect of MICT on body weight in college students with overweight or obesity.

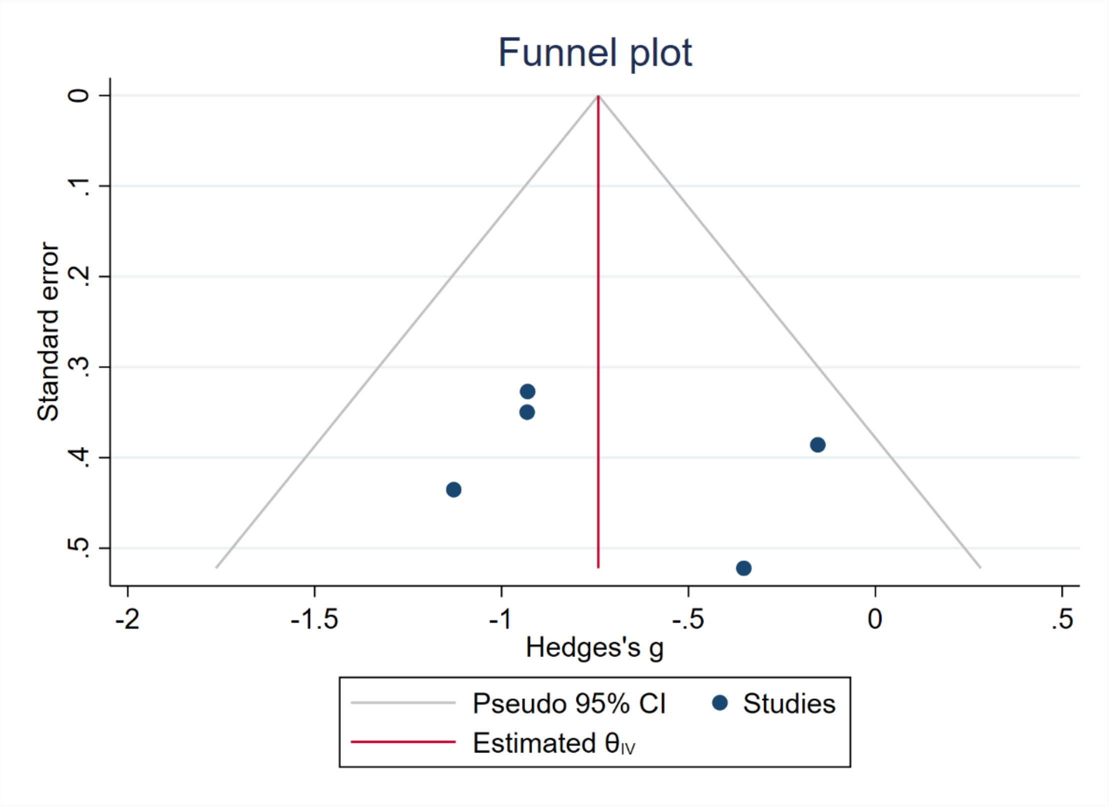

Figure S15. Funnel plot of the effect of MICT on BMI in college students with overweight and obesity.

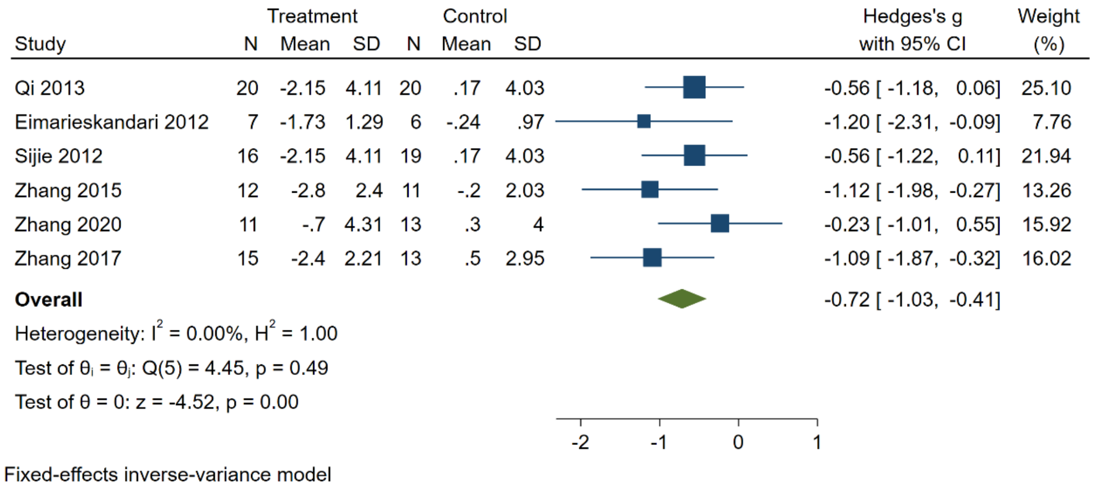

Figure S16. Forest plot of the effect of MICT on body fat percentage in college students with overweight and obesity.

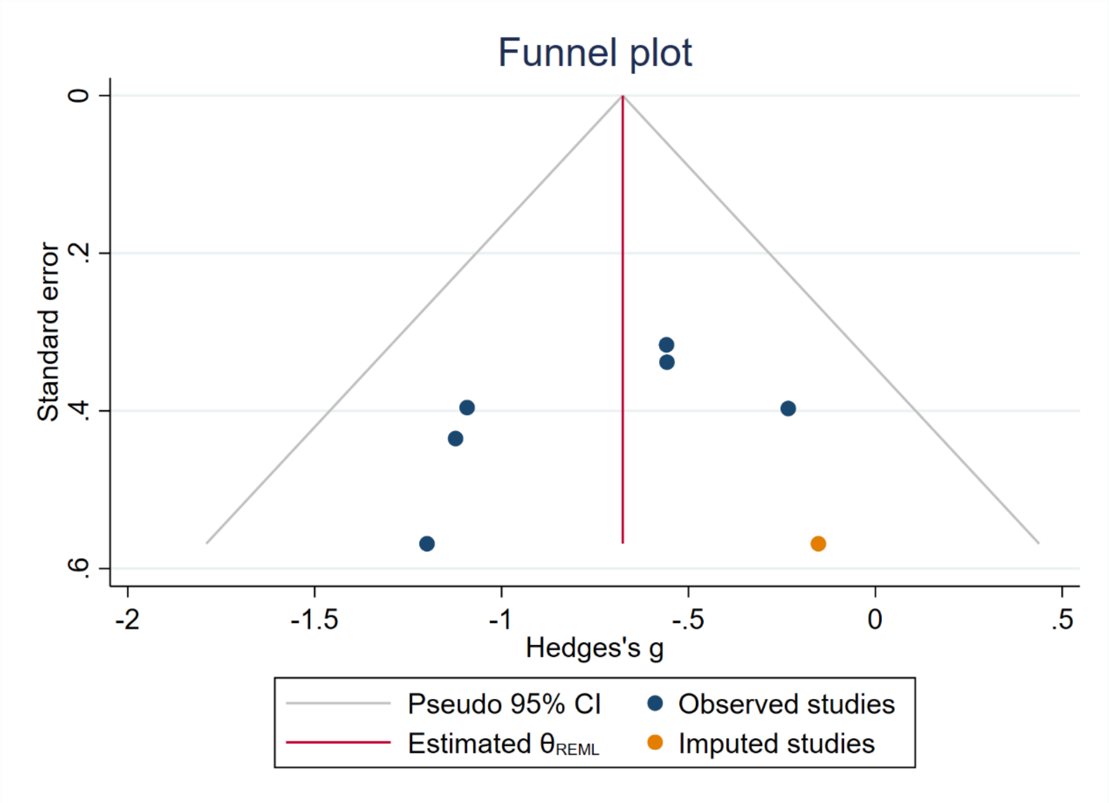

Figure S17. Funnel plot of the effect of MICT on body fat percentage in college students with overweight and obesity.

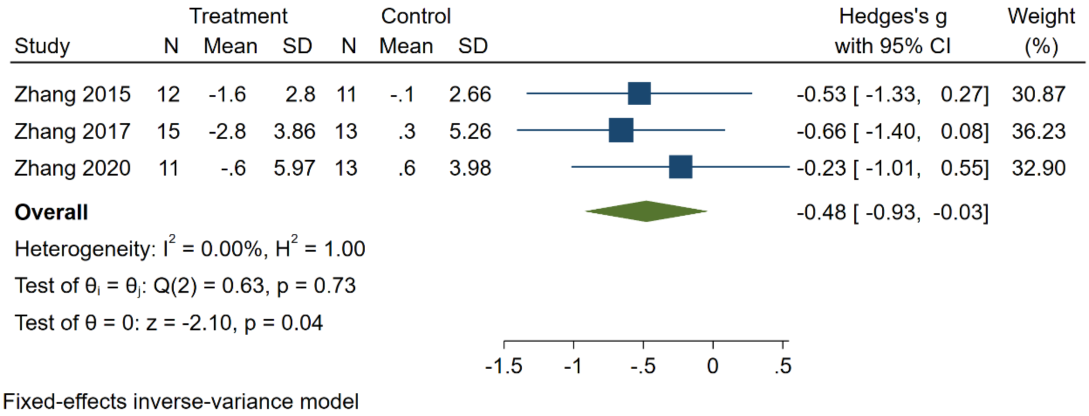

Figure S18. Forest plot of the effect of MICT on fat mass in college students with overweight and obesity.

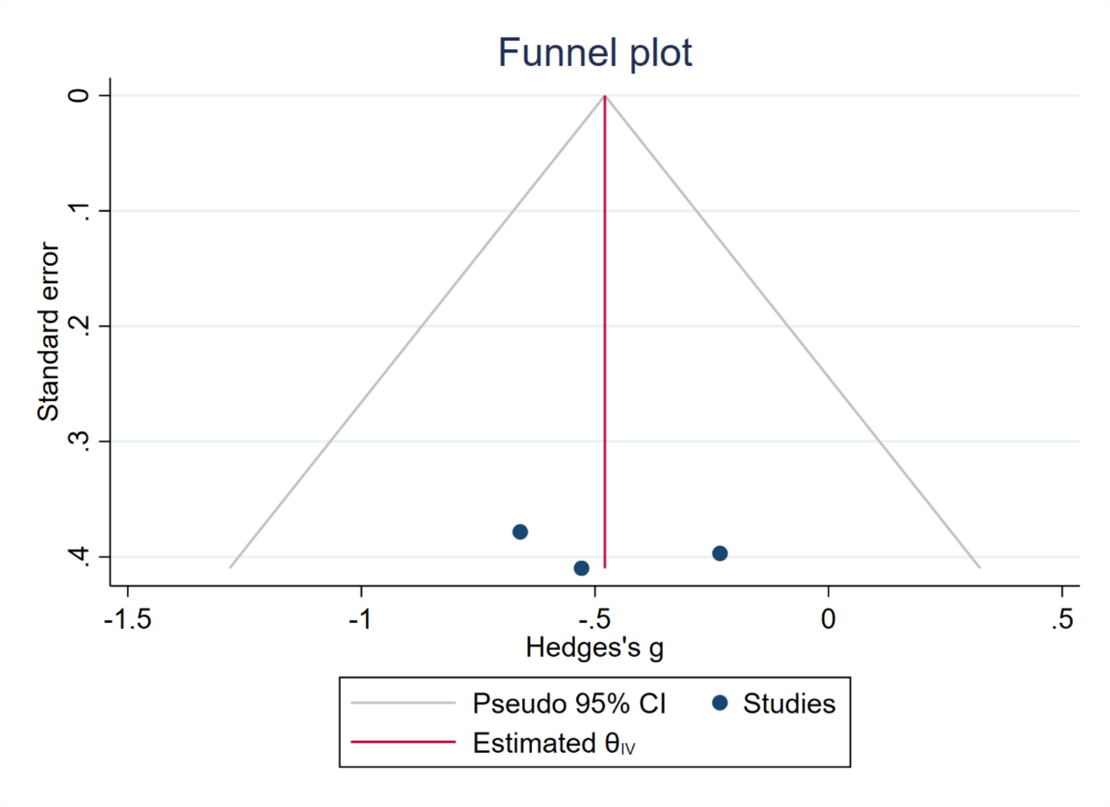

Figure S19. Funnel plot of the effect of MICT on fat mass in college students with overweight and obesity.

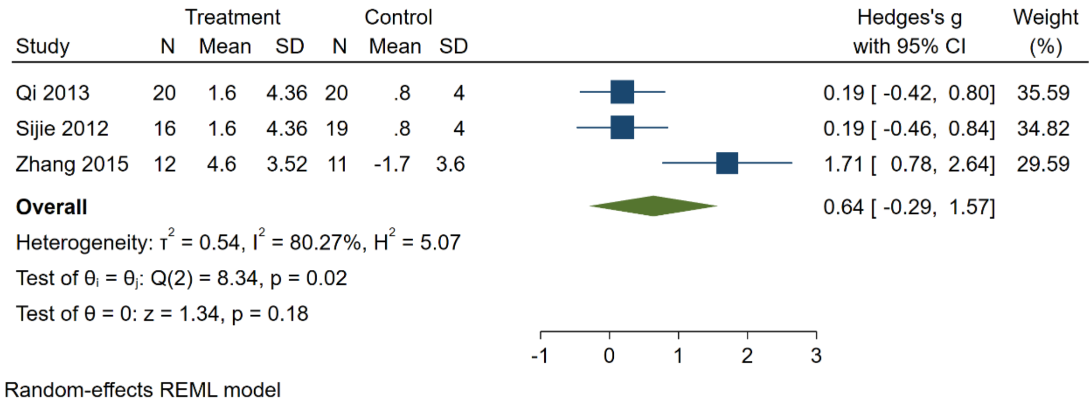

Figure S20. Forest plot of the effect of MICT on cardiorespiratory fitness in college students with overweight and obesity.

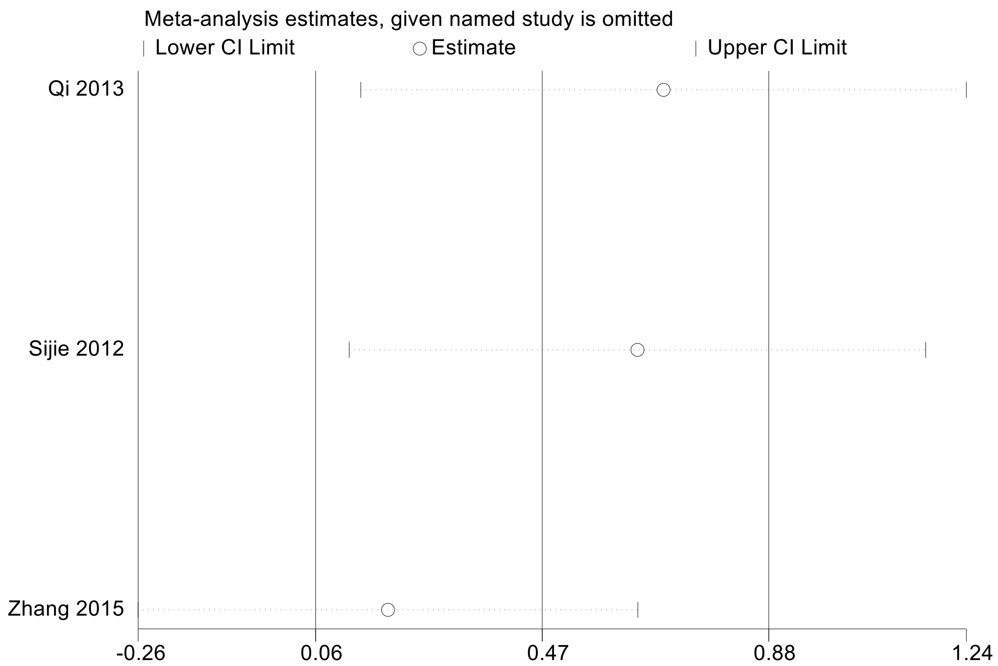

Figure S21. Sensitivity analysis of the effect of MICT on cardiorespiratory fitness in college students with overweight and obesity.

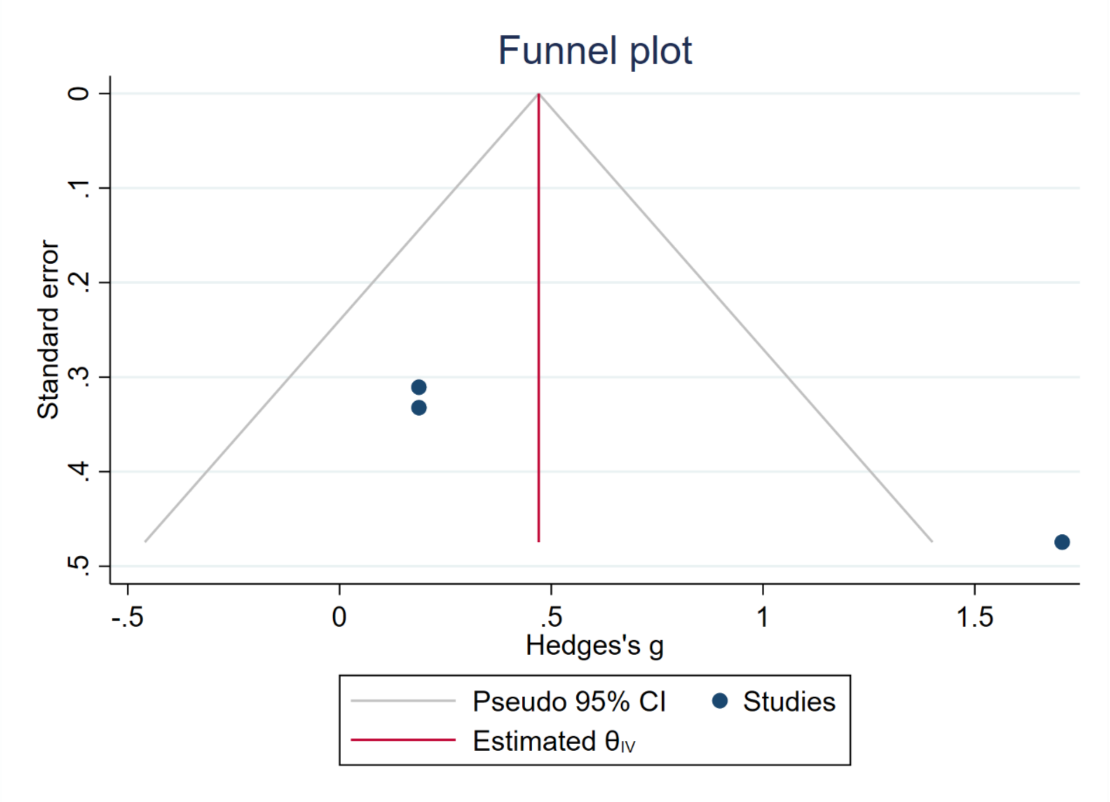

Figure S22. Funnel plot of the effect of MICT on cardiorespiratory fitness in college students with overweight and obesity.

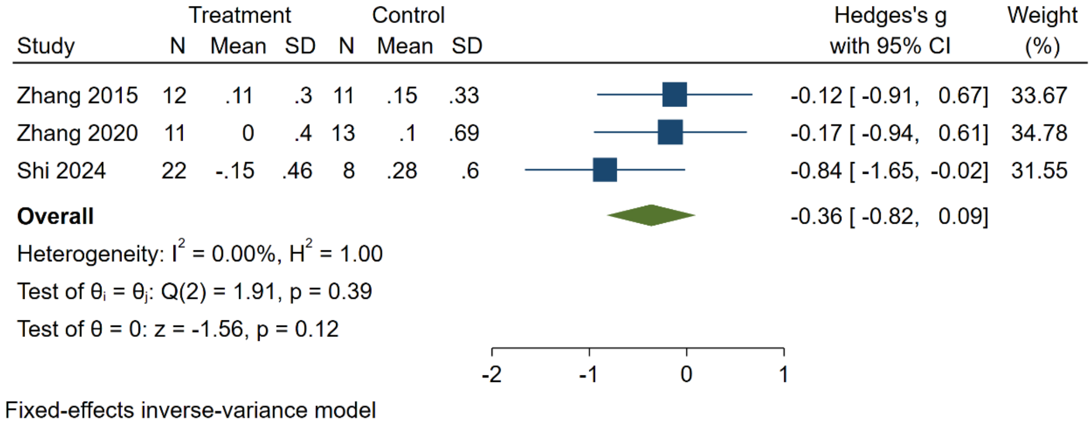

Figure S23. Forest plot of the effect of MICT on triglycerides (TG) in college students with overweight and obesity.

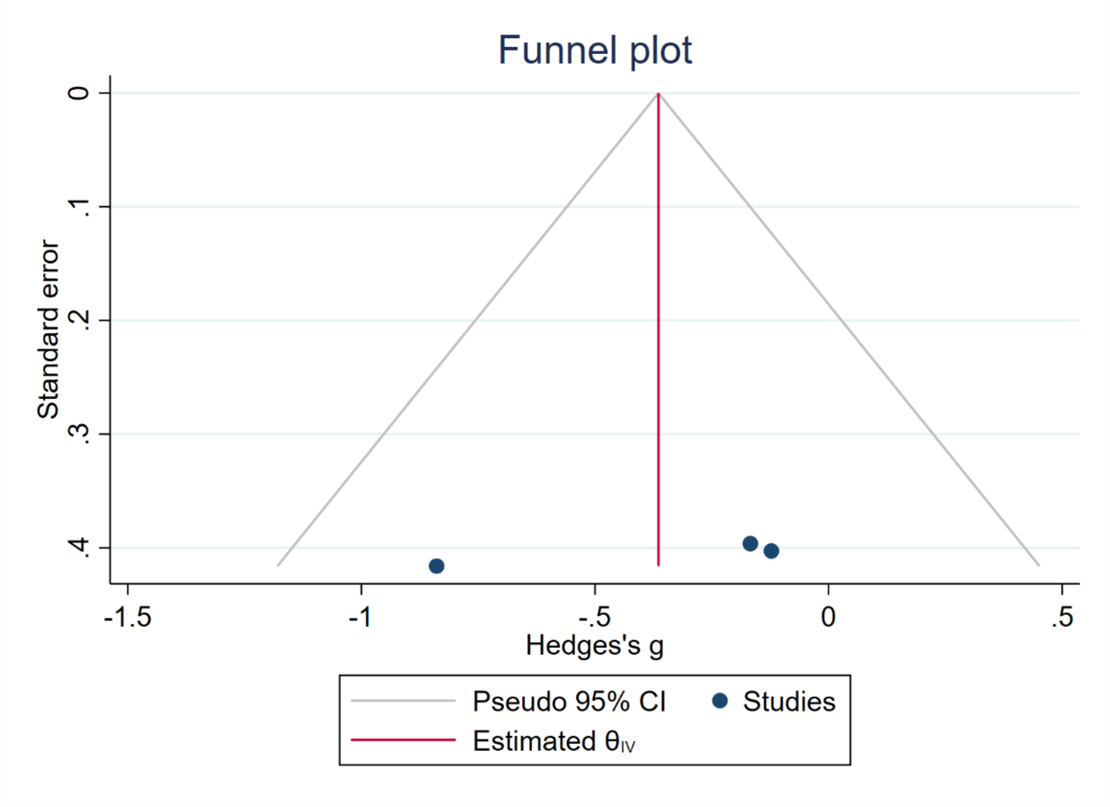

Figure S24. Funnel plot of the effect of MICT on TG in college students with overweight and obesity.

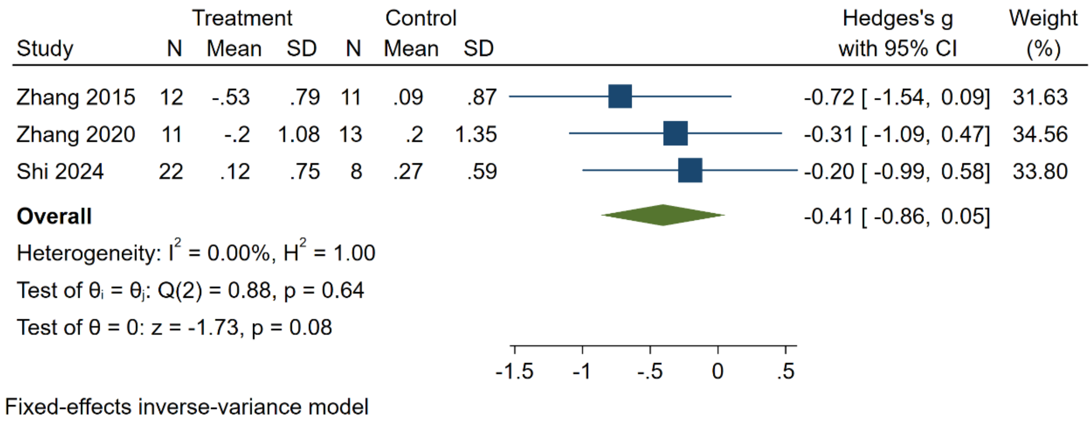

Figure S25. Forest plot of the effect of MICT on TC in college students with overweight and obesity.

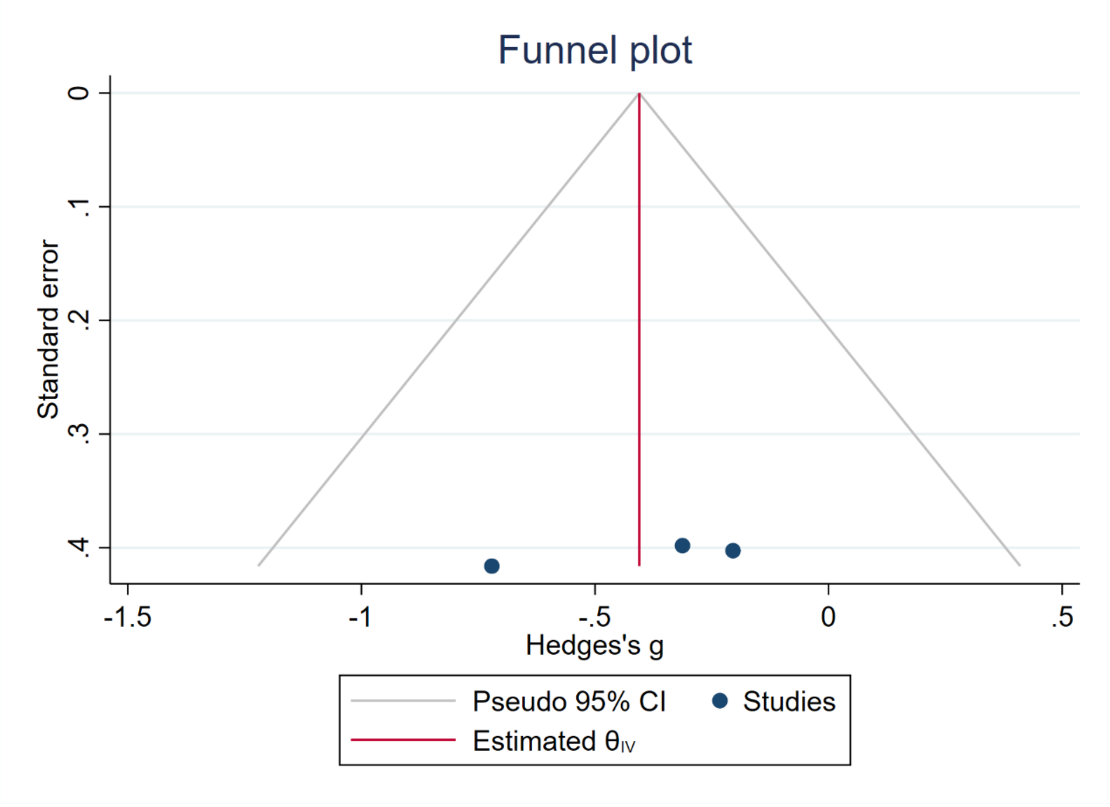

Figure S26. Funnel plot of the effect of MICT on TC in college students with overweight and obesity.

HIIT vs MICT

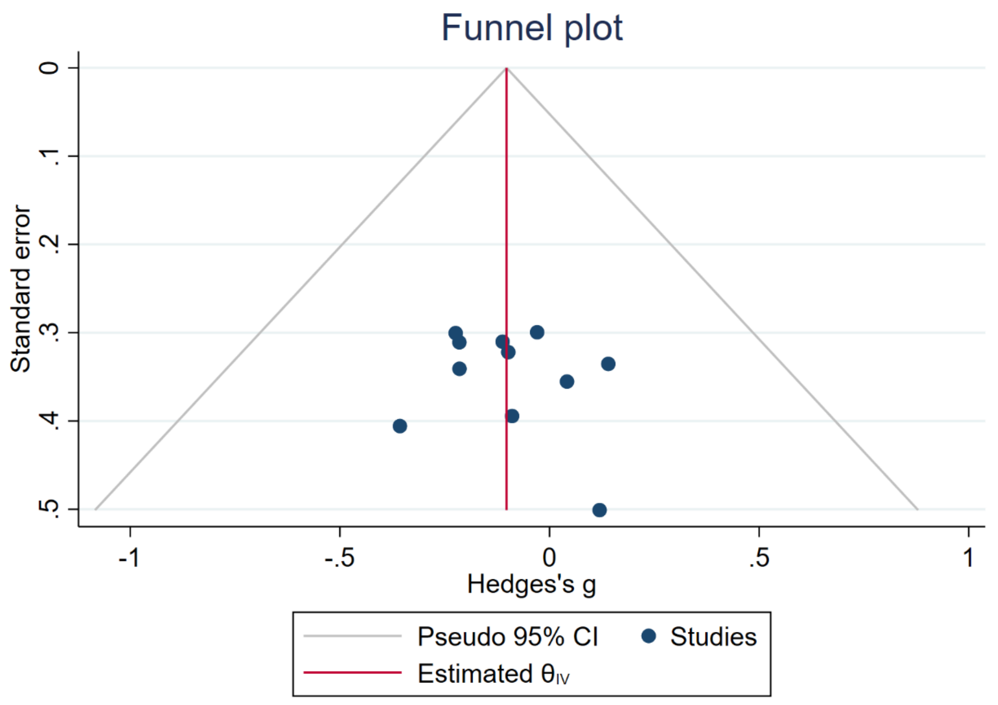

Figure S27. Funnel plot comparing the effects of HIIT and MICT on body weight in college students with overweight and obesity.

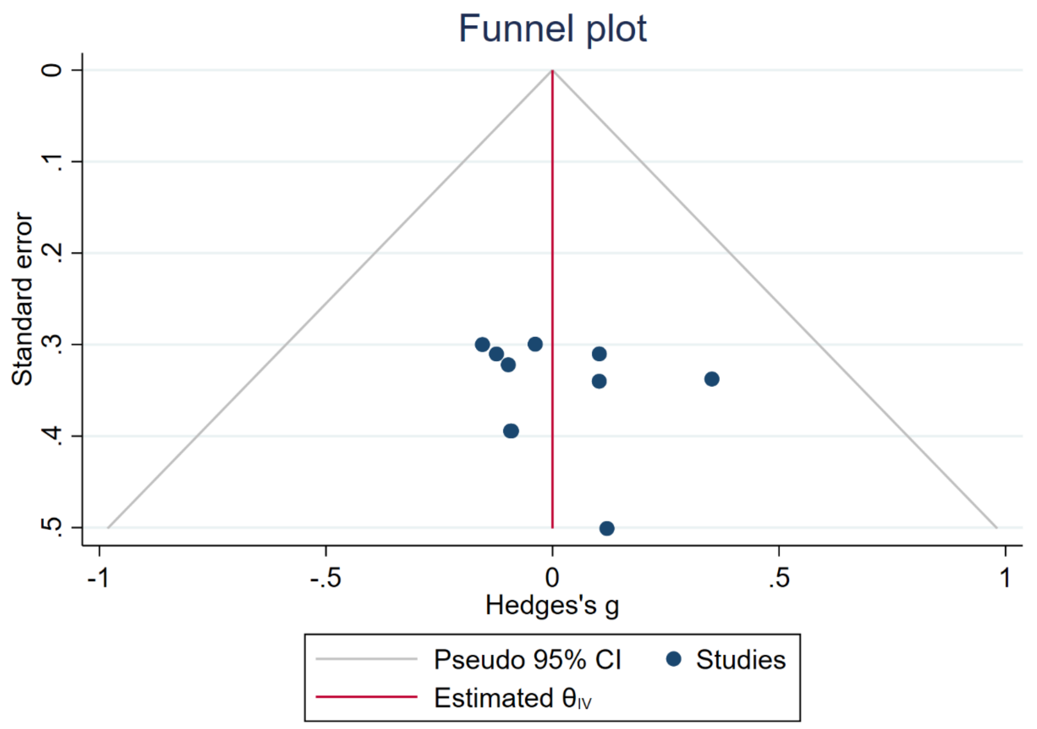

Figure S28. Funnel plot comparing the effects of HIIT and MICT on BMI in college students with overweight and obesity.

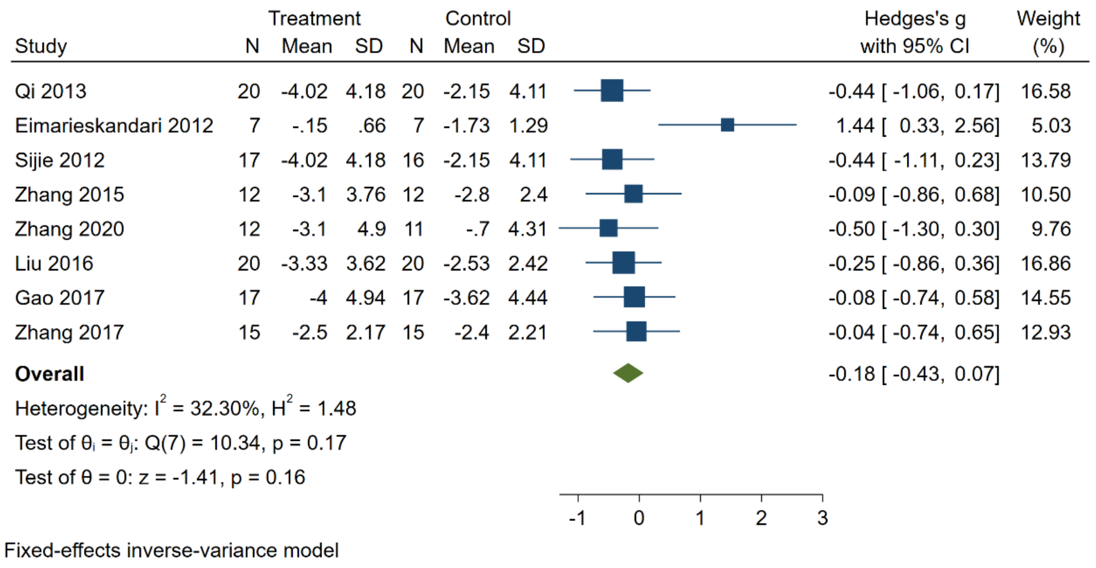

Figure S29. Forest plot comparing the effects of HIIT and MICT on body fat percentage in college students with overweight and obesity.

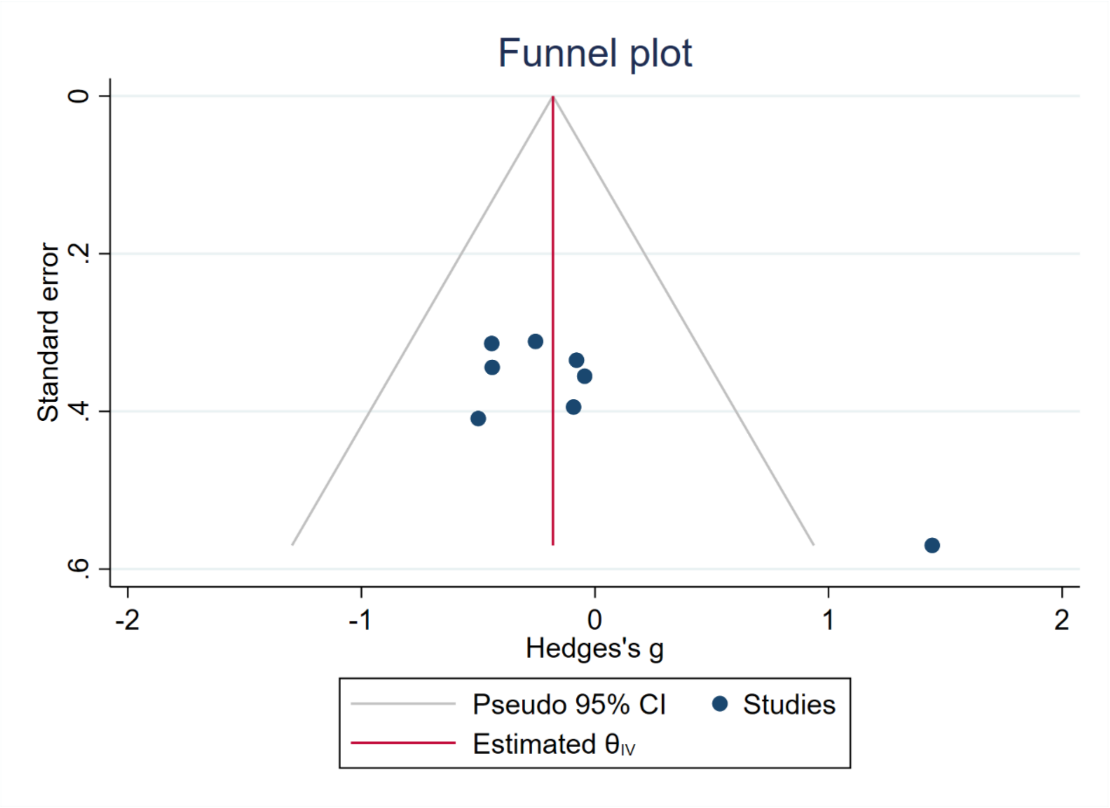

Figure S30. Funnel plot comparing the effects of HIIT and MICT on body fat percentage in college students with overweight and obesity.

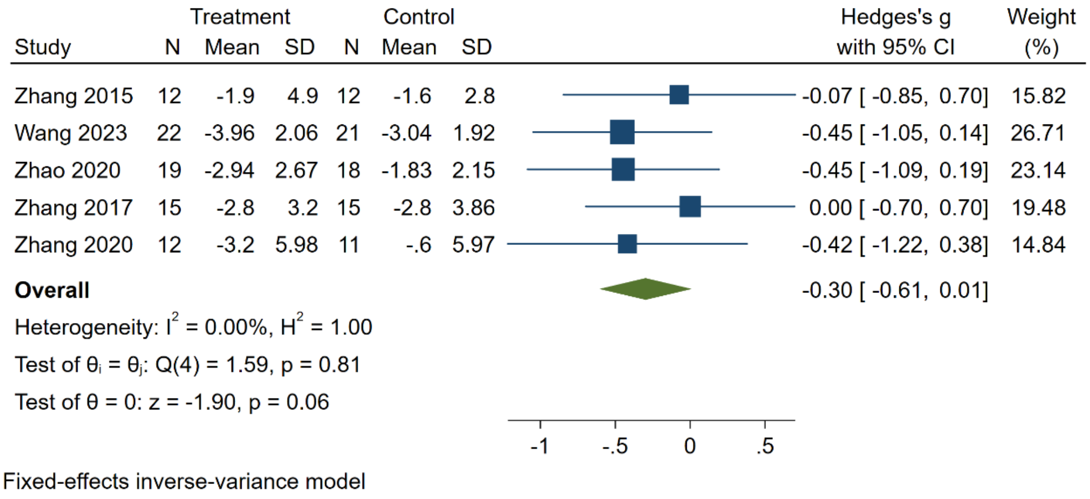

Figure S31. Forest plot comparing the effects of HIIT and MICT on fat mass in college students with overweight and obesity.

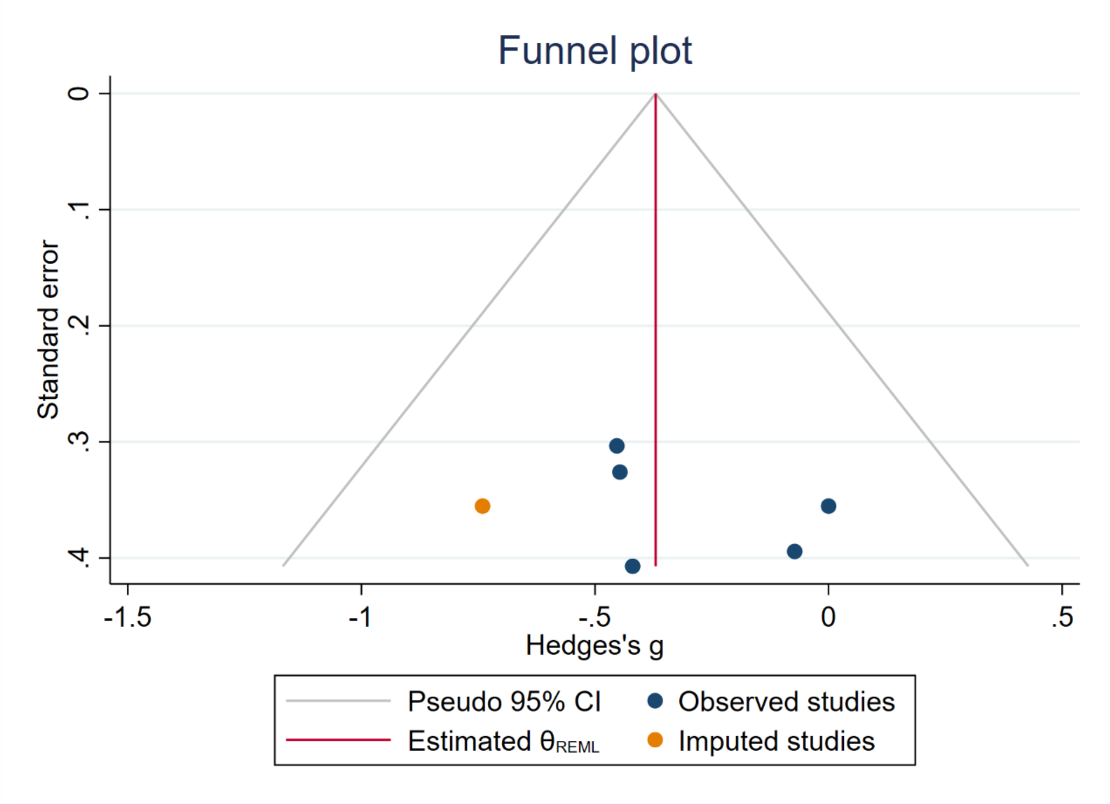

Figure S32. Funnel plot comparing the effects of HIIT and MICT on fat mass in college students with overweight and obesity.

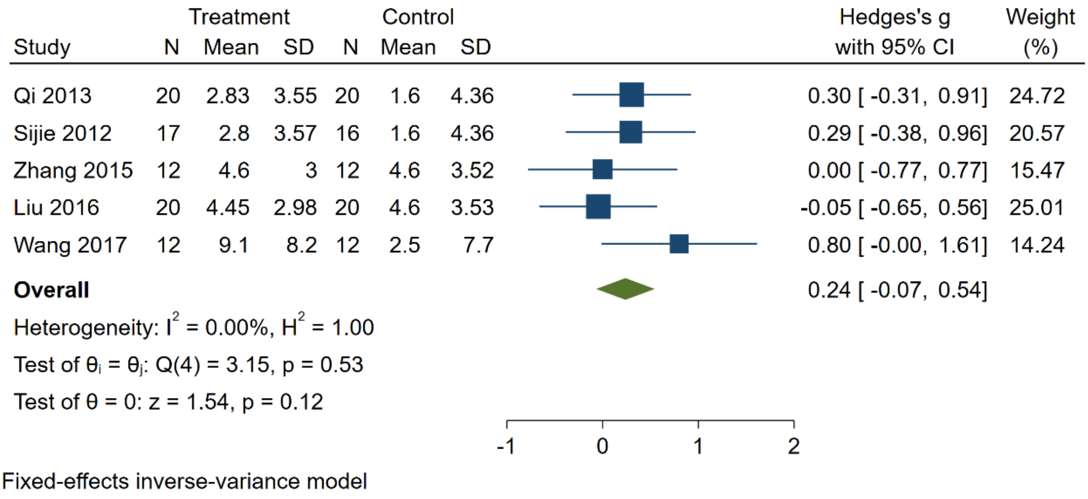

Figure S33. Forest plot comparing the effects of HIIT and MICT on cardiorespiratory fitness in college students with overweight and obesity.

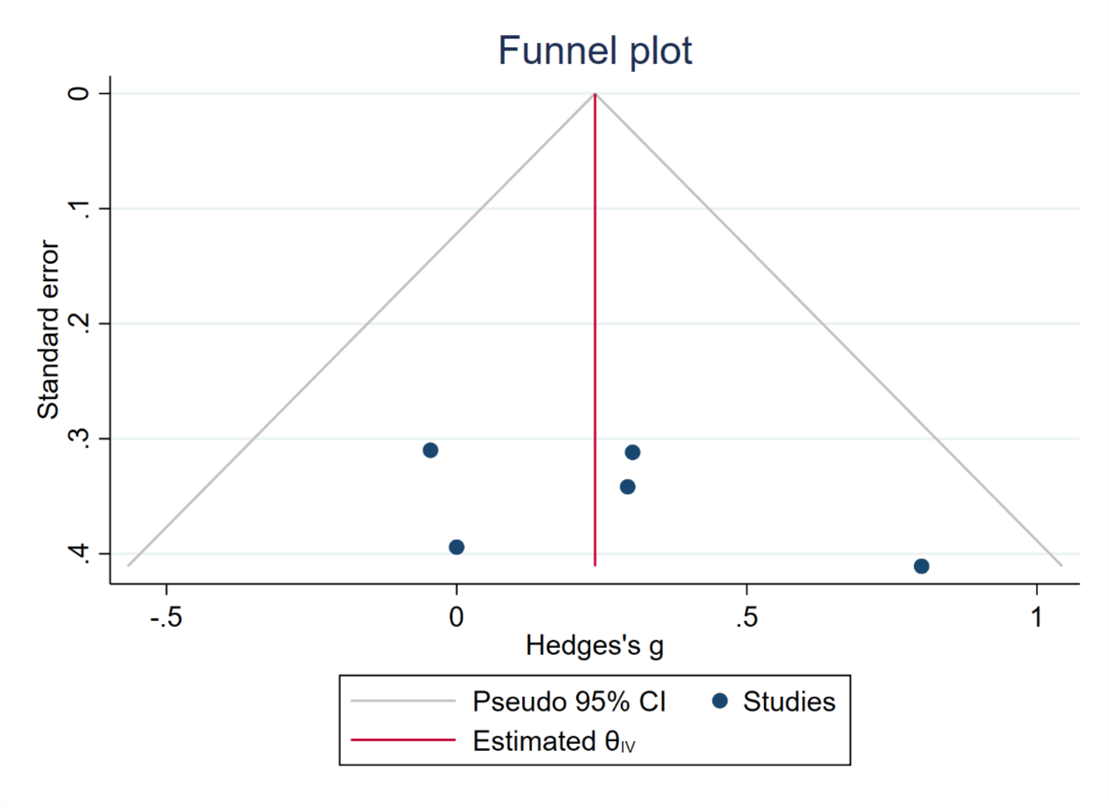

Figure S34. Funnel plot comparing the effects of HIIT and MICT on cardiorespiratory fitness in college students with overweight and obesity.

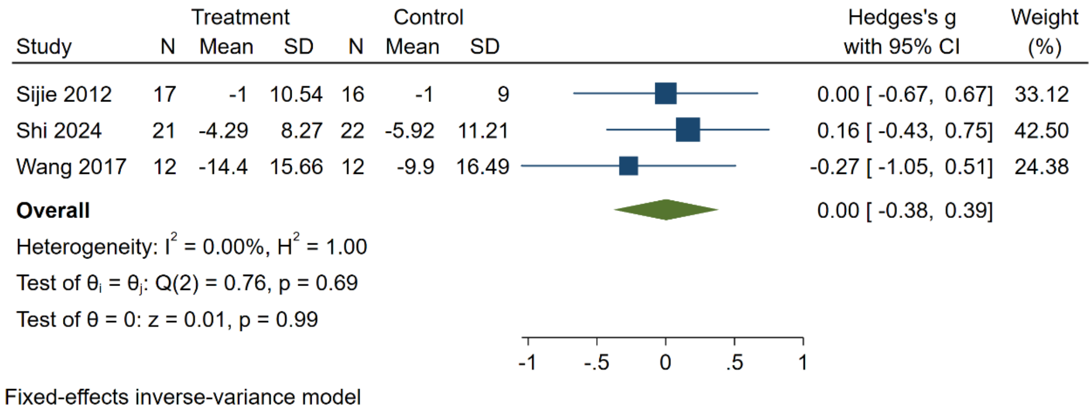

Figure S35. Forest plot comparing the effects of HIIT and MICT on systolic blood pressure in college students with overweight and obesity.

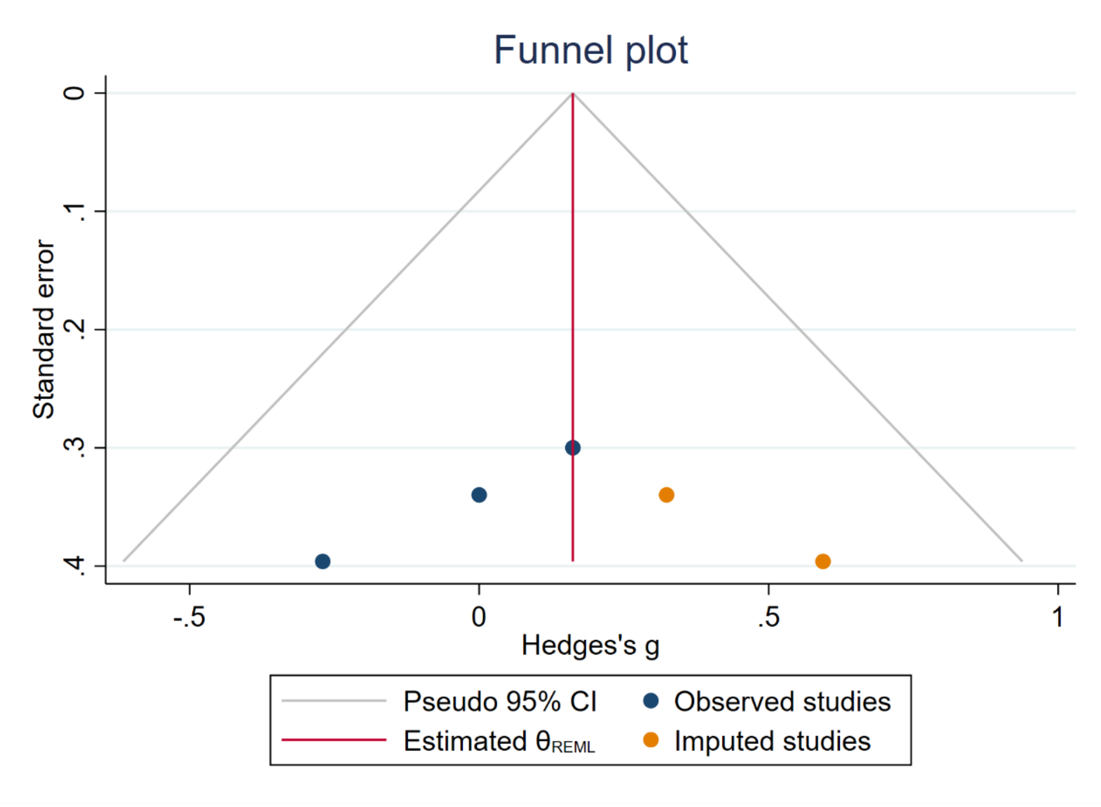

Figure S36. Funnel plot comparing the effects of HIIT and MICT on systolic blood pressure in college students with overweight and obesity.

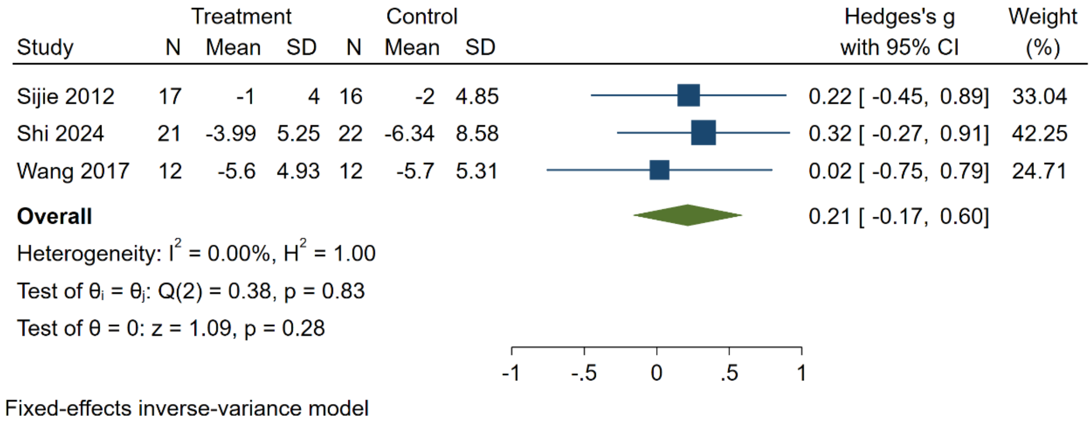

Figure S37. Forest plot comparing the effects of HIIT and MICT on diastolic blood pressure in college students with overweight and obesity.

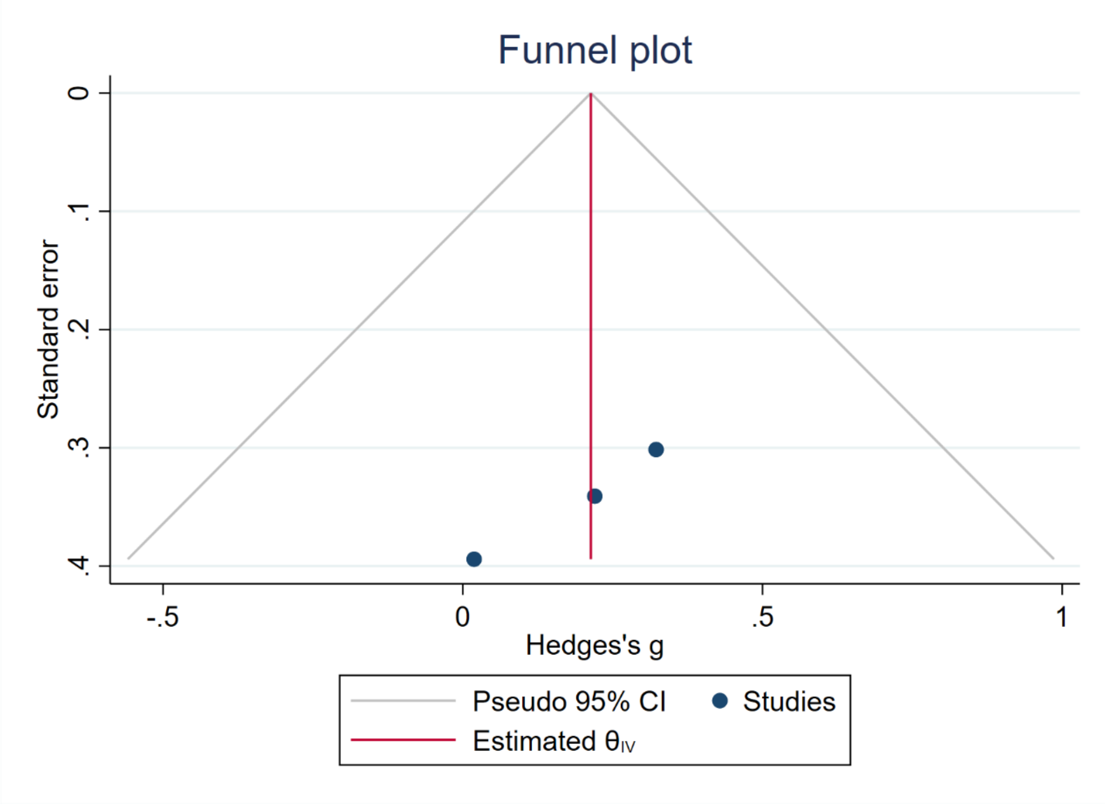

Figure S38. Funnel plot comparing the effects of HIIT and MICT on diastolic blood pressure in college students with overweight and obesity.

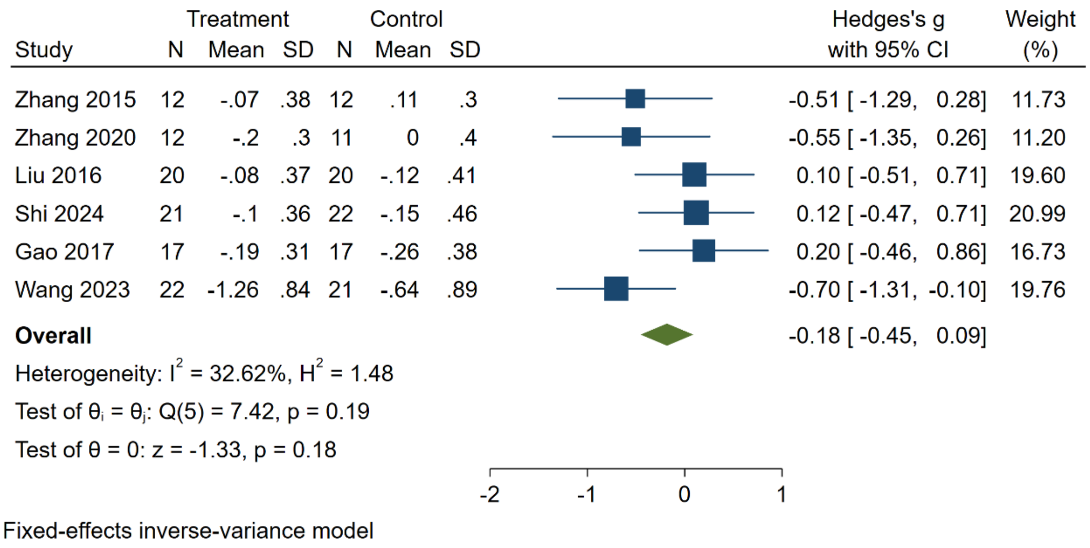

Figure S39. Forest plot comparing the effects of HIIT and MICT on TG in college students with overweight and obesity.

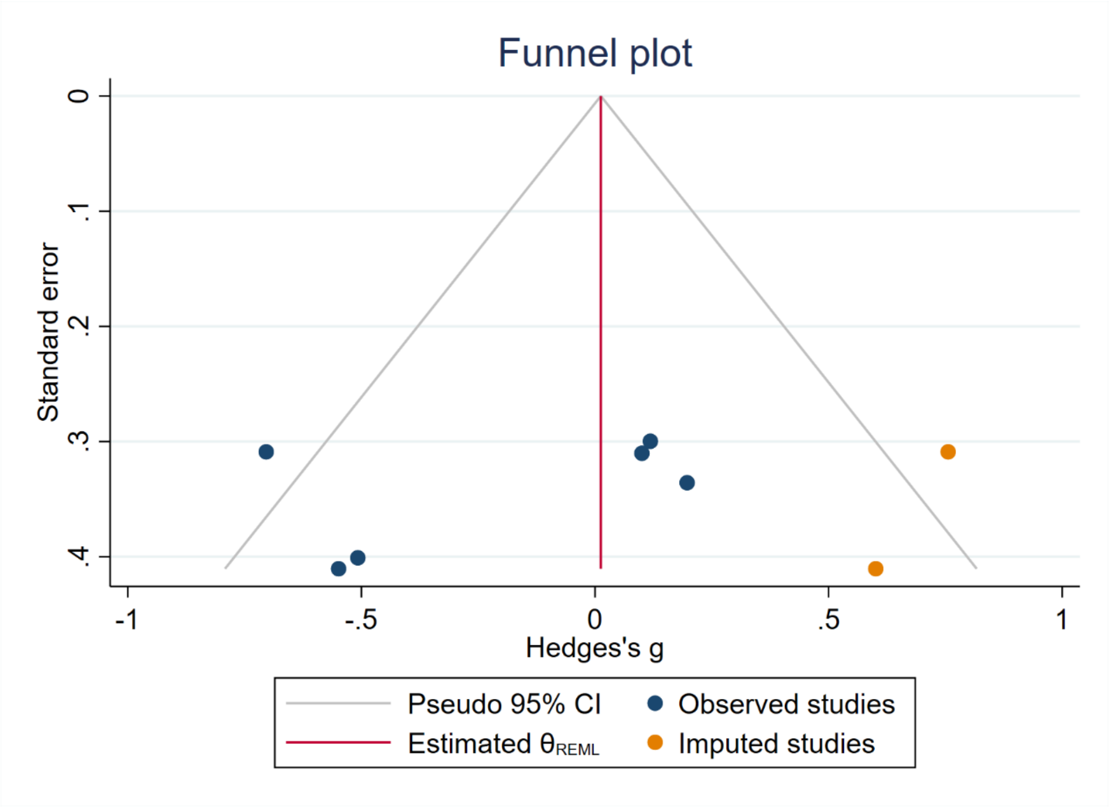

Figure S40. Funnel plot comparing the effects of HIIT and MICT on TG in college students with overweight and obesity.

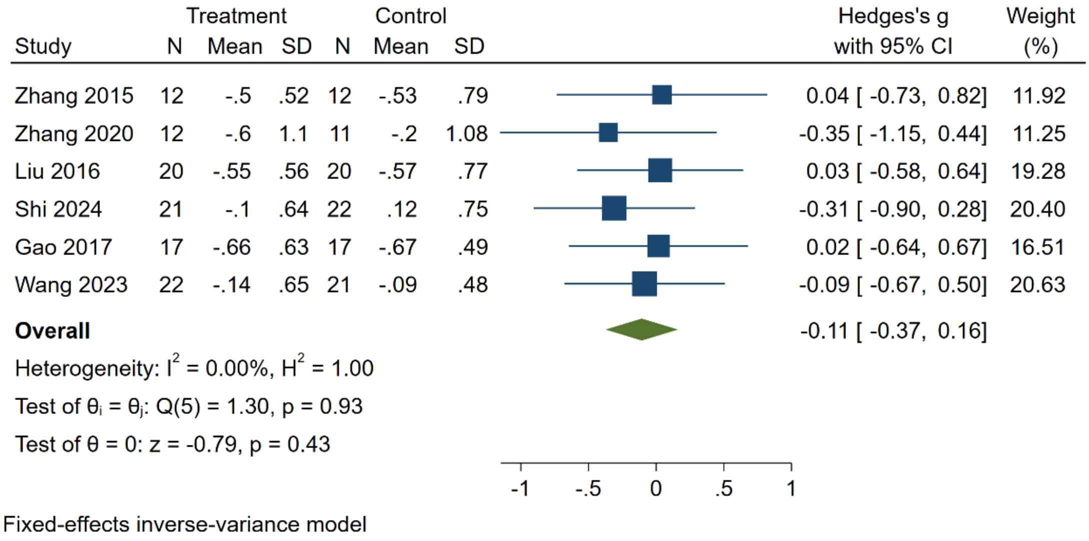

Figure S41. Forest plot comparing the effects of HIIT and MICT on TC in college students with overweight and obesity.

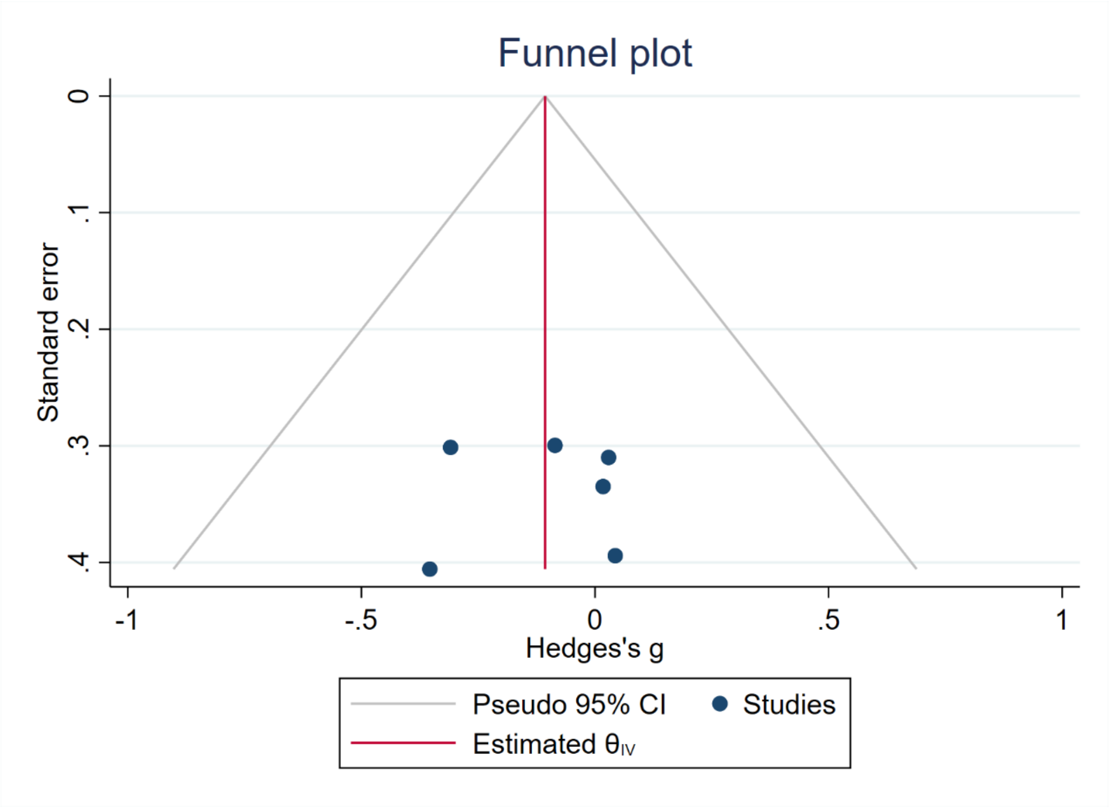

Figure S42. Funnel plot comparing the effects of HIIT and MICT on TC in college students with overweight and obesity.

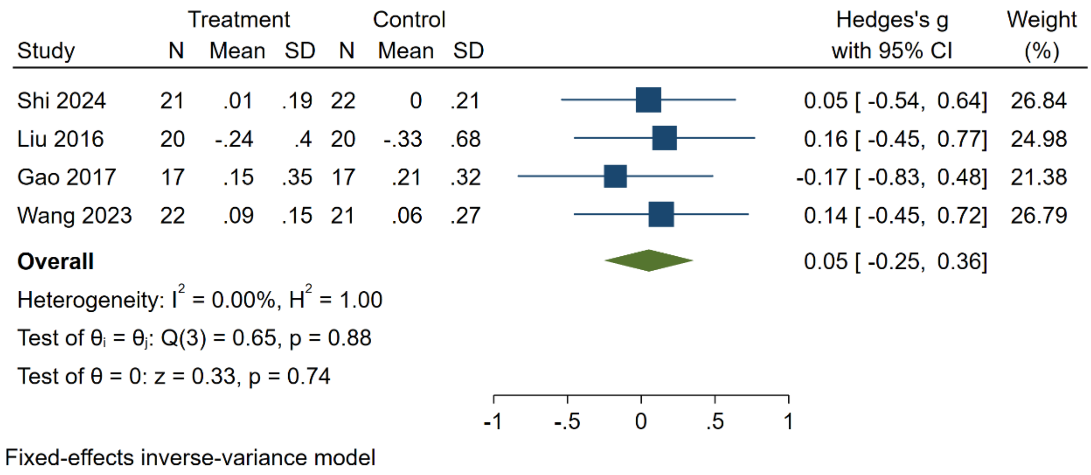

Figure S43. Forest plot comparing the effects of HIIT and MICT on high-density lipoprotein in college students with overweight and obesity.

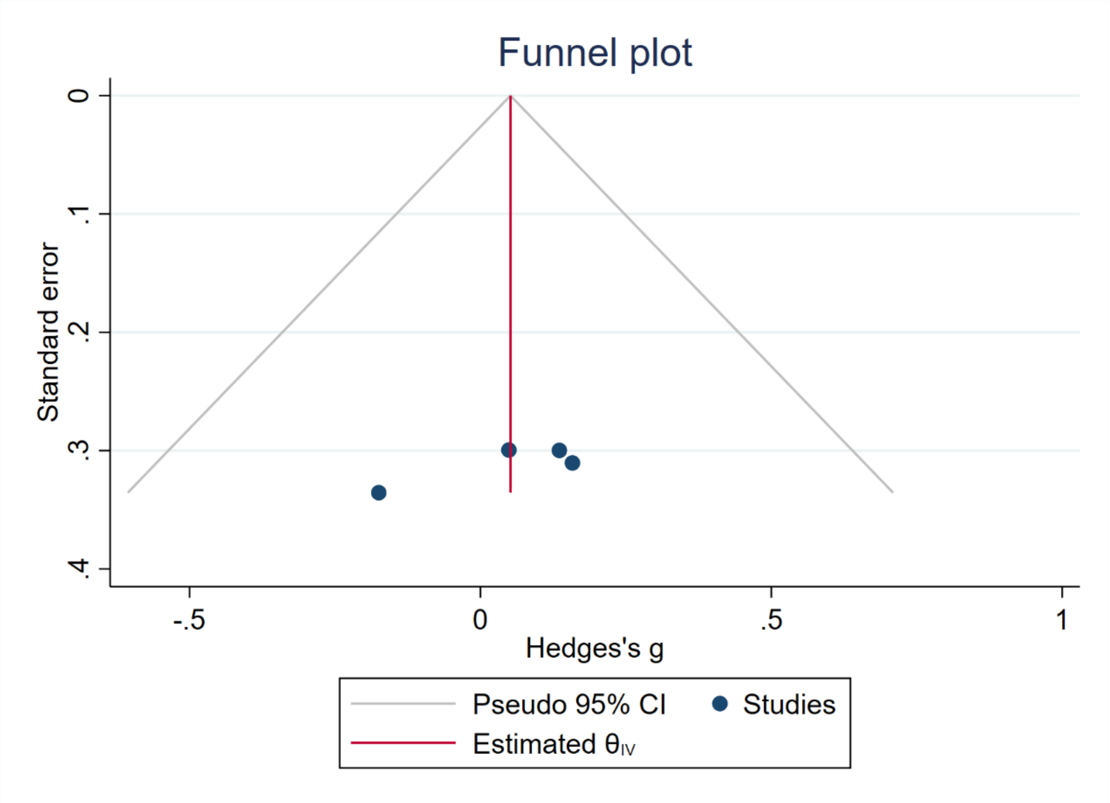

Figure S44. Funnel plot comparing the effects of HIIT and MICT on high-density lipoprotein in college students with overweight and obesity.

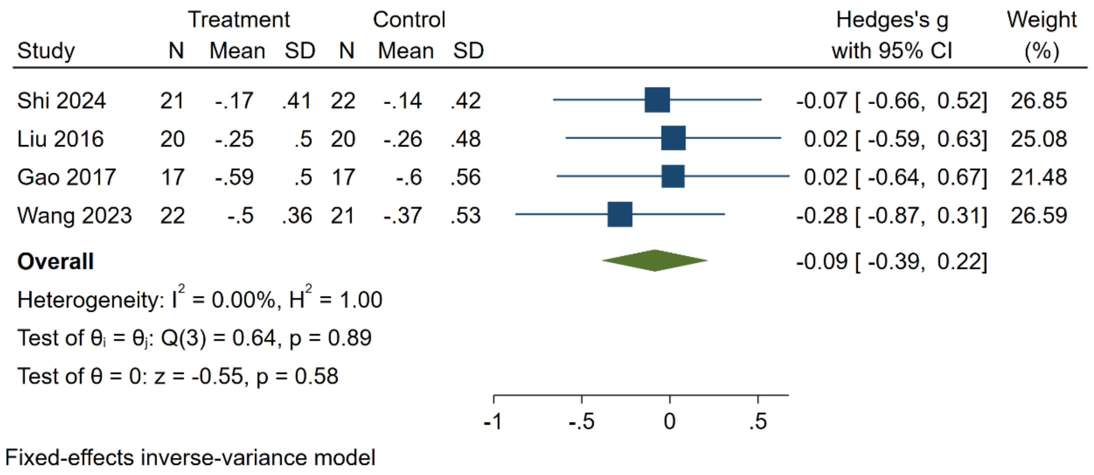

Figure S45. Forest plot comparing the effects of HIIT and MICT on low-density lipoprotein in college students with overweight and obesity.

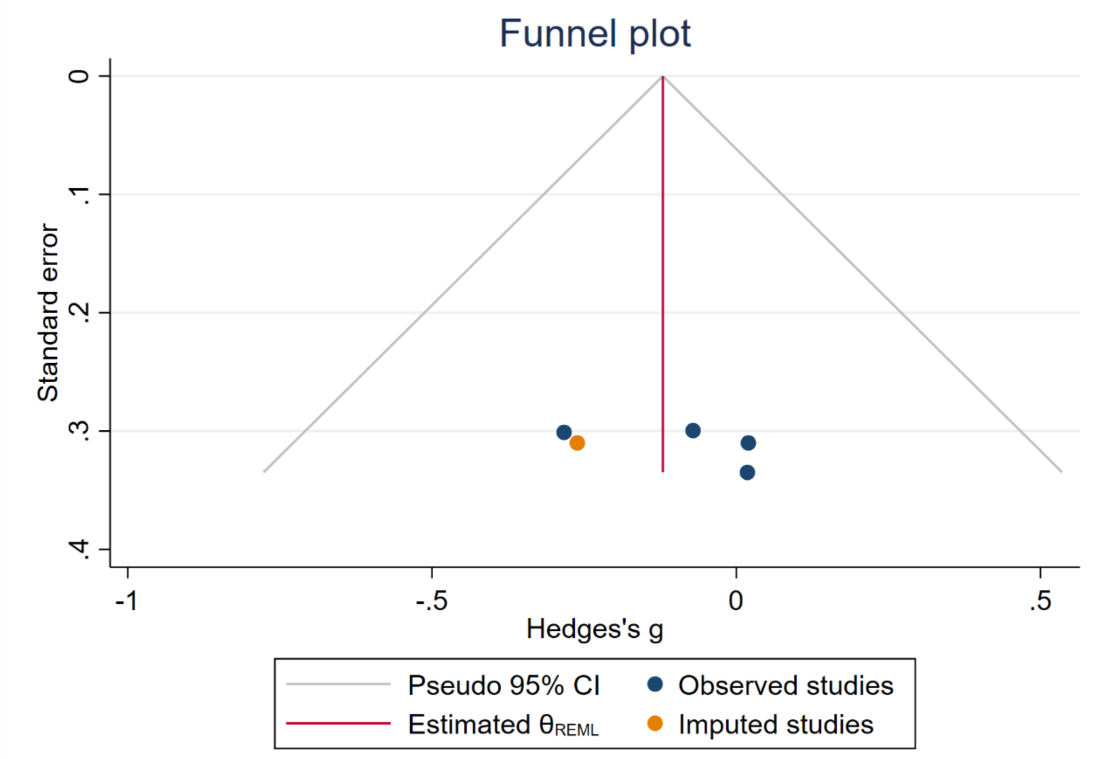

Figure S46. Funnel plot comparing the effects of HIIT and MICT on low-density lipoprotein in college students with overweight and obesity.

Table S1. Literature Search Strategy

Database	Literature Search Strategy
CNKI	#1 高强度间歇训练(High-Intensity Interval Training) + HIIT + 间歇训练(Interval Training)<br>#2 肥胖(Obesity) + 超重(Overweight)<br>#3 大学生(college students) + 学生(students)<br>#4 #1 AND #2 AND #3
Pubmed	#1 "High-Intensity Interval Training"[Mesh] OR "high-intensity interval training"[Title/Abstract] OR "high-intensity intermittent exercise"[Title/Abstract] OR "aerobic interval training"[Title/Abstract] OR "high-intensity intermittent"[Title/Abstract] OR "sprint interval training"[Title/Abstract] OR "sprint interval exercise" or "HIIT"[Title/Abstract] OR "HIT"[Title/Abstract] OR "SIT"[Title/Abstract]<br>#2 "Obesity"[Mesh] OR "Overweight"[Mesh] OR "obesity"[Title/Abstract] OR "obese"[Title/Abstract] OR "fat"[Title/Abstract] OR "corpulence"[Title/Abstract] OR "adiposis"[Title/Abstract] OR "overweight"[Title/Abstract]<br>#3 "college students"[Title/Abstract] OR "youth"[Title/Abstract] OR "undergraduate"[Title/Abstract] OR "University student"[Title/Abstract] OR "adolescents"[Title/Abstract] OR "young adults"[Title/Abstract]<br>#4 randomized controlled trial[Publication Type] OR "randomized controlled trial"[Title/Abstract] OR "randomized"[Title/Abstract] OR "trial"[Title/Abstract] OR "controlled"[Title/Abstract] OR "placebo"[Title/Abstract] OR "RCT"[Title/Abstract] OR "Single-blinded Method"[Title/Abstract] OR "Double-blinded Method"[Title/Abstract]<br>#5 #1 AND #2 AND #3 AND #4
WOS	#1 TS=(“high-intensity interval training” or “high-intensity intermittent exercise” or “aerobic interval training” or “high-intensity intermittent” or “sprint interval training” or ”sprint interval exercise” or ”HIIT” or “HIT” or "SIT”)<br>#2 TS=(“obesity” or “obese” or “fat” or "corpulence” OR “adiposis” OR “overweight”)<br>#3 TS=("college students" OR "youth" OR "undergraduate" OR "University student" OR "adolescents" OR "young adults")<br>#4 TS=(“randomized controlled trial” OR "randomized" OR "trial" OR "controlled" OR "placebo" OR "RCT" OR "Single-blinded Method" OR "Double-blinded Method")<br>#5 #1 and #2 and #3 and #4
Embase	#1 'high intensity interval training'/exp OR 'high-intensity interval training':ab,ti OR 'high-intensity intermittent exercise':ab,ti OR 'aerobic interval training':ab,ti OR 'high-intensity intermittent':ab,ti OR 'sprint interval training':ab,ti OR 'sprint interval exercise':ab,ti OR 'hiit':ab,ti OR 'hit':ab,ti OR 'sit':ab,ti<br>#2 'obesity'/exp OR 'overweight'/exp OR 'obesity':ab,ti OR 'obese':ab,ti OR 'fat':ab,ti OR 'corpulence':ab,ti OR 'adiposis':ab,ti OR 'overweight':ab,ti<br>#3 'college students'/exp OR 'college students':ab,ti OR 'youth':ab,ti OR 'undergraduate':ab,ti OR 'university student':ab,ti OR 'adolescents':ab,ti OR 'young adults':ab,ti<br>#4 'randomized controlled trial'/exp OR 'randomized controlled trial':ab,ti OR 'randomized':ab,ti OR 'trial':ab,ti OR 'controlled':ab,ti OR 'placebo':ab,ti OR 'rct':ab,ti OR 'single-blinded method':ab,ti OR 'double-blinded method':ab,ti<br>#5 #1 AND #2 AND #3 AND #4
the Cochrane Library	#1 MeSH descriptor: [High-Intensity Interval Training] explode all trees<br>#2 (“high-intensity interval training” or “high-intensity intermittent exercise” or “aerobic interval training” or “high-intensity intermittent” or “sprint interval training” or ”sprint interval exercise” or ”HIIT” or “HIT” or ”SIT”):ti,ab,kw<br>#3 #1 or #2<br>#4 MeSH descriptor: [Obesity] explode all trees<br>#5 MeSH descriptor: [Overweight] explode all trees<br>#6 (“obesity” or “obese” or “fat” or ”corpulence” or “adiposis” or “overweight”):ti,ab,kw<br>#7 #4 OR #5 OR #6<br>#8 ("college students" or "youth" or "undergraduate" or "University student" or "adolescents" or "young adults"):ti,ab,kw<br>#9 (“randomized controlled trial” or "randomized" or "trial" or "controlled", "placebo" or "RCT" or "Single-blinded Method" or "Double-blinded Method"):ti,ab,kw<br>#10 #3 AND #7 AND #8 AND #9

Note: CNKI for China National Knowledge Infrastructure. WOS for Web of Science.

Table S2 GRADE Evidence Quality Assessment

	Study Limitations	Inconsistencies	Indirectness	Imprecision	Publication Bias	GRADE
**HIIT vs Control**						
Body weight	Downgrade 1 Level[A]	Not Downgraded	Not Downgraded	Downgrade 1 Level[B]	Not Downgraded	Low
BMI	Downgrade 1 Level[A]	Not Downgraded	Not Downgraded	Downgrade 1 Level[B]	Not Downgraded	Low
Body fat percentage	Downgrade 1 Level[A]	Not Downgraded	Not Downgraded	Downgrade 1 Level[B]	Not Downgraded	Low
Fat mass	Downgrade 1 Level[A]	Not Downgraded	Not Downgraded	Downgrade 1 Level[B]	Not Downgraded	Low
Cardiorespiratory fitness	Downgrade 1 Level[A]	Downgrade 1 Level[C]	Not Downgraded	Downgrade 1 Level[B]	Not Downgraded	Very Low
TG	Downgrade 1 Level[A]	Not Downgraded	Not Downgraded	Downgrade 1 Level[B]	Not Downgraded	Low
TC	Downgrade 1 Level[A]	Not Downgraded	Not Downgraded	Downgrade 1 Level[B]	Not Downgraded	Low
**MICT vs Control**						
Body weight	Downgrade 1 Level[A]	Not Downgraded	Not Downgraded	Downgrade 1 Level[B]	Not Downgraded	Low
BMI	Downgrade 1 Level[A]	Not Downgraded	Not Downgraded	Downgrade 1 Level[B]	Not Downgraded	Low
Body fat percentage	Downgrade 1 Level[A]	Not Downgraded	Not Downgraded	Downgrade 1 Level[B]	Not Downgraded	Low
Fat mass	Downgrade 1 Level[A]	Not Downgraded	Not Downgraded	Downgrade 1 Level[B]	Not Downgraded	Low
Cardiorespiratory fitness	Downgrade 1 Level[A]	Downgrade 2 Level[D]	Not Downgraded	Downgrade 2 Level[E]	Not Downgraded	Very Very Low
TG	Downgrade 1 Level[A]	Not Downgraded	Not Downgraded	Downgrade 2 Level[E]	Not Downgraded	Very Low
TC	Downgrade 1 Level[A]	Not Downgraded	Not Downgraded	Downgrade 2 Level[E]	Not Downgraded	Very Low
**HIIT vs MICT**						
Body weight	Downgrade 1 Level[A]	Not Downgraded	Not Downgraded	Downgrade 2 Level[E]	Not Downgraded	Very low
BMI	Downgrade 1 Level[A]	Not Downgraded	Not Downgraded	Downgrade 2 Level[E]	Not Downgraded	Very low
Body fat percentage	Downgrade 1 Level[A]	Not Downgraded	Not Downgraded	Downgrade 2 Level[E]	Not Downgraded	Very low
Fat mass	Downgrade 1 Level[A]	Not Downgraded	Not Downgraded	Downgrade 2 Level[E]	Not Downgraded	Very low
Cardiorespiratory fitness	Downgrade 1 Level[A]	Not Downgraded	Not Downgraded	Downgrade 2 Level[E]	Not Downgraded	Very low
Systolic blood pressure	Downgrade 1 Level[A]	Not Downgraded	Not Downgraded	Downgrade 2 Level[E]	Not Downgraded	Very low
Diastolic blood pressure	Downgrade 1 Level[A]	Downgrade 2 Level[D]	Not Downgraded	Downgrade 2 Level[E]	Not Downgraded	Very low
TG	Downgrade 1 Level[A]	Downgrade 1 Level[C]	Not Downgraded	Downgrade 2 Level[E]	Not Downgraded	Very low
TC	Downgrade 1 Level[A]	Not Downgraded	Not Downgraded	Downgrade 2 Level[E]	Not Downgraded	Very low
High-density lipoprotein	Downgrade 1 Level[A]	Not Downgraded	Not Downgraded	Downgrade 2 Level[E]	Not Downgraded	Very low
Low-density lipoprotein	Downgrade 1 Level[A]	Not Downgraded	Not Downgraded	Downgrade 2 Level[E]	Not Downgraded	Very low

A for Two-thirds of the included studies were rated as "some concerns" on the RoB 2 tool. B for The included studies generally had small sample sizes. C for Between-study heterogeneity was high(I^2=50%-75%). D for Between-study heterogeneity was high(I^2 > 75%%). E for The 95% confidence interval crossed zero, and the sample size was small.

PRISMA 2020 Checklist

Section and Topic	Item #	Checklist item	Location where item is reported
**TITLE**			
Title	1	Identify the report as a systematic review.	1-3
**ABSTRACT**			
Abstract	2	See the PRISMA 2020 for Abstracts checklist.	5-56
**INTRODUCTION**			
Rationale	3	Describe the rationale for the review in the context of existing knowledge.	61-132
Objectives	4	Provide an explicit statement of the objective(s) or question(s) the review addresses.	133-149
**METHODS**			
Eligibility criteria	5	Specify the inclusion and exclusion criteria for the review and how studies were grouped for the syntheses.	678-703
Information sources	6	Specify all databases, registers, websites, organisations, reference lists and other sources searched or consulted to identify studies. Specify the date when each source was last searched or consulted.	705-716
Search strategy	7	Present the full search strategies for all databases, registers and websites, including any filters and limits used.	supplementary materials
Selection process	8	Specify the methods used to decide whether a study met the inclusion criteria of the review, including how many reviewers screened each record and each report retrieved, whether they worked independently, and if applicable, details of automation tools used in the process.	718-724
Data collection process	9	Specify the methods used to collect data from reports, including how many reviewers collected data from each report, whether they worked independently, any processes for obtaining or confirming data from study investigators, and if applicable, details of automation tools used in the process.	726-737
Data items	10a	List and define all outcomes for which data were sought. Specify whether all results that were compatible with each outcome domain in each study were sought (e.g. for all measures, time points, analyses), and if not, the methods used to decide which results to collect.	726-737
	10b	List and define all other variables for which data were sought (e.g. participant and intervention characteristics, funding sources). Describe any assumptions made about any missing or unclear information.	726-737
Study risk of bias assessment	11	Specify the methods used to assess risk of bias in the included studies, including details of the tool(s) used, how many reviewers assessed each study and whether they worked independently, and if applicable, details of automation tools used in the process.	739-775
Effect measures	12	Specify for each outcome the effect measure(s) (e.g. risk ratio, mean difference) used in the synthesis or presentation of results.	778-787
Synthesis methods	13a	Describe the processes used to decide which studies were eligible for each synthesis (e.g. tabulating the study intervention characteristics and comparing against the planned groups for each synthesis (item #5)).	778-787
	13b	Describe any methods required to prepare the data for presentation or synthesis, such as handling of missing summary statistics, or data conversions.	778-787
	13c	Describe any methods used to tabulate or visually display results of individual studies and syntheses.	778-787
	13d	Describe any methods used to synthesize results and provide a rationale for the choice(s). If meta-analysis was performed, describe the model(s), method(s) to identify the presence and extent of statistical heterogeneity, and software package(s) used.	778-787
	13e	Describe any methods used to explore possible causes of heterogeneity among study results (e.g. subgroup analysis, meta-regression).	778-787
	13f	Describe any sensitivity analyses conducted to assess robustness of the synthesized results.	778-787
Reporting bias assessment	14	Describe any methods used to assess risk of bias due to missing results in a synthesis (arising from reporting biases).	778-787
Certainty	15	Describe any methods used to assess certainty (or confidence) in the body of evidence for an outcome.	778-787

PRISMA 2020 Checklist

Section and Topic	Item #	Checklist item	Location where item is reported
assessment			
**RESULTS**			
Study selection	16a	Describe the results of the search and selection process, from the number of records identified in the search to the number of studies included in the review, ideally using a flow diagram.	Figure 1
	16b	Cite studies that might appear to meet the inclusion criteria, but which were excluded, and explain why they were excluded.	151-161
Study characteristics	17	Cite each included study and present its characteristics.	Table1,2
Risk of bias in studies	18	Present assessments of risk of bias for each included study.	195-203
Results of individual studies	19	For all outcomes, present, for each study: (a) summary statistics for each group (where appropriate) and (b) an effect estimate and its precision (e.g. confidence/credible interval), ideally using structured tables or plots.	207-417
Results of syntheses	20a	For each synthesis, briefly summarise the characteristics and risk of bias among contributing studies.	207-417
	20b	Present results of all statistical syntheses conducted. If meta-analysis was done, present for each the summary estimate and its precision (e.g. confidence/credible interval) and measures of statistical heterogeneity. If comparing groups, describe the direction of the effect.	207-417
	20c	Present results of all investigations of possible causes of heterogeneity among study results.	207-417
	20d	Present results of all sensitivity analyses conducted to assess the robustness of the synthesized results.	207-417
Reporting biases	21	Present assessments of risk of bias due to missing results (arising from reporting biases) for each synthesis assessed.	207-417
Certainty of evidence	22	Present assessments of certainty (or confidence) in the body of evidence for each outcome assessed.	207-417
**DISCUSSION**			
Discussion	23a	Provide a general interpretation of the results in the context of other evidence.	420-593
	23b	Discuss any limitations of the evidence included in the review.	420-593
	23c	Discuss any limitations of the review processes used.	420-593
	23d	Discuss implications of the results for practice, policy, and future research.	595-621
**OTHER INFORMATION**			
Registration and protocol	24a	Provide registration information for the review, including register name and registration number, or state that the review was not registered.	672-676
	24b	Indicate where the review protocol can be accessed, or state that a protocol was not prepared.	672-676
	24c	Describe and explain any amendments to information provided at registration or in the protocol.	672-676
Support	25	Describe sources of financial or non-financial support for the review, and the role of the funders or sponsors in the review.	656-657
Competing interests	26	Declare any competing interests of review authors.	665
Availability of data, code and other materials	27	Report which of the following are publicly available and where they can be found: template data collection forms; data extracted from included studies; data used for all analyses; analytic code; any other materials used in the review.	643-644

From: Page MJ, McKenzie JE, Bossuyt PM, Boutron I, Hoffmann TC, Mulrow CD, et al. The PRISMA 2020 statement: an updated guideline for reporting systematic reviews. BMJ 2021;372:n71. doi: 10.1136/bmj.n71
